# Rapid atom-efficient polyolefin plastics hydrogenolysis mediated by a well-defined single-site electrophilic/cationic organo-zirconium catalyst

**DOI:** 10.1038/s41467-022-34707-6

**Published:** 2022-11-23

**Authors:** Alexander H. Mason, Alessandro Motta, Anusheela Das, Qing Ma, Michael J. Bedzyk, Yosi Kratish, Tobin J. Marks

**Affiliations:** 1 Northwestern University, Evanston, IL 60208 USA; 2 Università di Roma “La Sapienza” and INSTM, UdR Roma, Piazzale Aldo Moro 5, I-00185 Roma, Italy

**Keywords:** Heterogeneous catalysis, Catalytic mechanisms, Chemical engineering

## Abstract

Polyolefins comprise a major fraction of single-use plastics, yet their catalytic deconstruction/recycling has proven challenging due to their inert saturated hydrocarbon connectivities. Here a very electrophilic, formally cationic earth-abundant single-site organozirconium catalyst chemisorbed on a highly Brønsted acidic sulfated alumina support and characterized by a broad array of experimental and theoretical techniques, is shown to mediate the rapid hydrogenolytic cleavage of molecular and macromolecular saturated hydrocarbons under mild conditions, with catalytic onset as low as 90 °C/0.5 atm H_2_ with 0.02 mol% catalyst loading. For polyethylene, quantitative hydrogenolysis to light hydrocarbons proceeds within 48 min with an activity of > 4000 mol(CH_2_ units)·mol(Zr)^−1^·h^−1^ at 200 °C/2 atm H_2_ pressure. Under similar solventless conditions, polyethylene-*co*−1-octene, isotactic polypropylene, and a post-consumer food container cap are rapidly hydrogenolyzed to low molecular mass hydrocarbons. Regarding mechanism, theory and experiment identify a turnover-limiting C-C scission pathway involving *ß*-alkyl transfer rather than the more common σ-bond metathesis.

## Abstract (web summary)

Polyolefins are a major fraction of plastic waste, yet their catalytic recycling is challenging due to their inert hydrocarbon structures. Here a well-defined supported organozirconium catalyst is shown to mediate the rapid hydrogenolytic cleavage diverse polyolefins under very mild conditions.

## Introduction

Synthetic polymers play an essential role in modern society, providing necessary materials for food packaging, infrastructure, transportation, clothing, medical disposables, consumer electronics, etc. In 2018, ca.395 million tons were produced worldwide, with 1.1 billion tons (Gt) projected annually by 2050^1,2^. Since most plastics are single-use, global production has created a corresponding increase in derived waste and environmental impact^3,4^, with a cumulative 5.7 Gt of waste landfilled or incinerated to date^2^. Recycling is an attractive but underutilized means of repurposing plastics, decreasing fossil fuel reliance, and addressing plastics pollution^5–7^. However, most recycling today is mechanical, with polymers shredded and then re-shaped into materials with inferior properties compared to virgin materials^8^. Polyolefins comprise more than half of the production of modern synthetic polymers due to their low cost, enormous versatility, and chemical inertness. Today polyolefins such as polyethylene, polypropylene, and polystyrene are typically recycled pyrolytically at temperatures >400 °C. However, this energy-intensive unselective cracking yields hydrocarbon mixtures and significant residual carbonaceous coke^9^.

Catalytic polyolefin deconstruction to lighter hydrocarbons has proven challenging due to their chemical/thermal inertness. One homogeneous approach uses an Ir catalyst and metathesis with an alkane to lower the polyolefin molecular mass^10^. The conversion of polyethylene to long-chain alkylaromatics was also demonstrated using a Pt catalyst supported on γ-alumina^11^. Recently, catalytic polyolefin hydrogenolysis has attracted greater attention since it employs H_2_ as a cost-effective deconstruction agent, and the overall process is thermodynamically favorable. Currently, the most common approaches involve using heterogeneous precious-metal catalysts such as Ru and Pt to transform polyolefins into wax-range and lighter hydrocarbons. Such processes typically employ high temperatures/pressures and extended reaction times^12–17^.

**Fig. 1:**
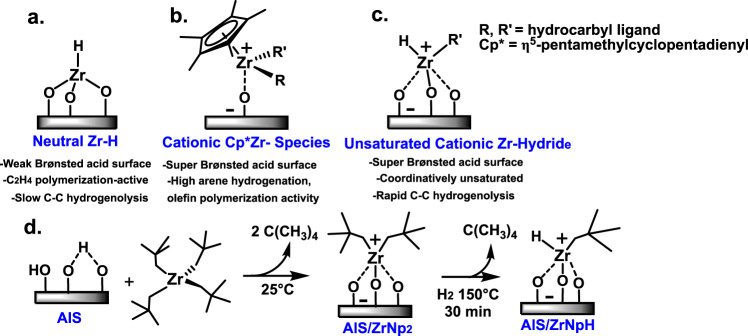
Schematic depiction of zirconium alkyl surface chemistry on various oxide surfaces. **a** Zr alkyl-derived adsorbate on a relatively weak Brønsted acidic surface, providing relatively strong conjugate Brønsted base counteranions, **b** Cp*Zr(CH_3_)_3_-derived adsorbate on a very strong Brønsted acidic surface (AlS = sulfated alumina). **c** ZrNp_4_-derived (Np = neopentyl) adsorbate on a very strong Brønsted acidic surface followed by H_2_ exposure. **d** ZrNp_4_ protonolytic chemisorption on strongly Brønsted acidic sulfated alumina (AlS), and generation of the corresponding electrophilic hydride via Zr–Np σ-bond hydrogenolysis.

Regarding earth-abundant metal polyolefin hydrogenolysis catalysts, the only example to our knowledge employed a formally neutrally charged d^0^ Zr alkyl bound to silica-alumina, which mediates relatively slow hydrogenolysis (Fig. 1a). This formally neutrally charged Zr alkyl was reported by Basset et al. to have ca. 3% of the activity now found for the catalyst reported here (vide infra)^18^. We hypothesized that the protonolytic chemisorption of Zr alkyls on very different, highly Brønsted acidic surfaces would create weak/weakly coordinating conjugate Brønsted base counteranions^19,20^, and would thereby create d^0^ catalysts sufficiently electrophilic/cationic to rapidly cleave polyolefin C–C bonds. Supporting this high activity hypothesis, note that chemisorbing Cp*Zr(CH_3_)_3_ (Cp*=η^5^-Me_5_C_5_)^21–23^ on such Brønsted acidic oxides yields methane via Zr–CH_3_ bond protonolysis and an electrostatically bound, formally cationic organozirconium adsorbate (Fig. 1b). This surface-bound formally cationic species catalyzes very rapidly, facially selective arene hydrogenation and olefin polymerization with nearly 100% of the Zr sites catalytically significant^21–23^. Such catalysts operate via mechanisms differing substantially from later transition metals, raising the intriguing question of whether they might rapidly activate/hydrogenolyze polyolefins via well-defined but unconventional pathways.

Here we report that ZrNp_4_ chemisorption on sulfated alumina (Hammett acidity, *H*_0_ ≈ −14.6)^24^ yields AlS/ZrNp_2_, characterized by solid-state nuclear magnetic resonance (NMR) spectroscopy and diffuse reflectance infrared Fourier transform spectroscopy (DRIFTS), inductively coupled plasma atomic emission spectroscopy (ICP-AES), X-ray absorption near-edge structure (XANES), extended X-ray absorption fine structure (EXAFS), and density-functional theory (DFT) computation (Fig. 1c, d). We report that AlS/ZrNp_2_ catalyzes the rapid, solventless hydrogenolysis of polyethylene (PE), isotactic polypropylene (*i*-PP), polyethylene-*co*−1-octene (PECO) copolymer, and consumer PE under very mild conditions^25^. For polyethylene, hydrogenolysis to light hydrocarbons proceeds with activity as high as 4000 mol(CH_2_ units) mol(Zr)^−1^ h^−1^ at 200 °C/2 atm H_2_ pressure. Here hexadecane (C16) is used as a PE model for kinetic/mechanistic investigations, which support a predominant rate-limiting C–C bond scission step via *ß*-alkyl transfer rather than σ-bond metathesis, which is common in *d*^0^ metal catalysis^26,27^, in accord with the DFT computation.

## Results

### Catalytic polyolefin hydrogenolysis

**Fig. 2:**
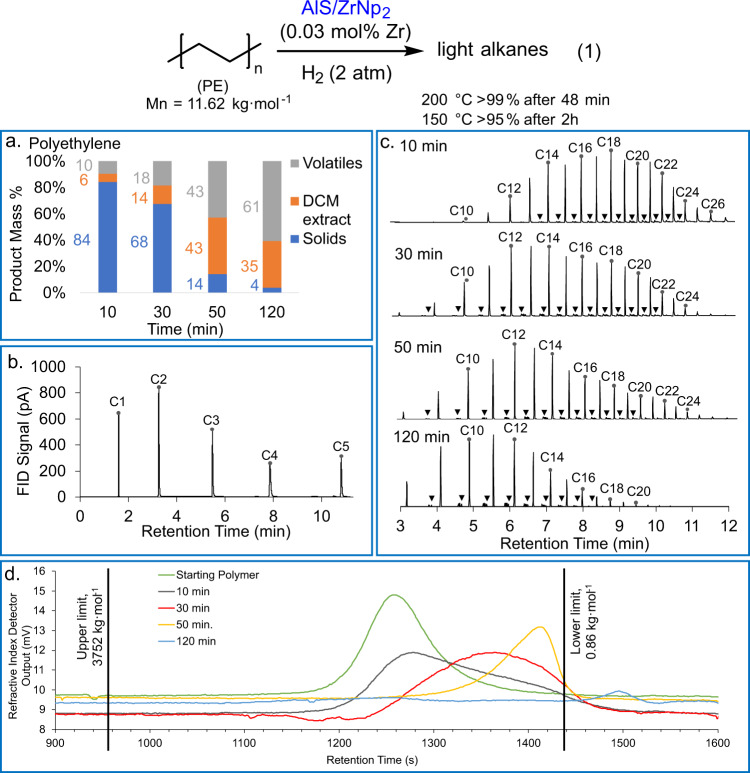
Catalytic hydrogenolysis of linear polyethylene (PE). **a** Temporal product distributions for AlS/ZrNp_2_-catalyzed PE hydrogenolysis (150 °C, 2.0 atm H_2_, 0.03 mol% Zr). **b** Headspace volatiles GC/FID chromatogram for 30 min PE hydrogenolysis. **c** GC/MS chromatograms of DCM extracts from AlS/ZrNp_2_-catalyzed PE hydrogenolysis. Trace chain branching is denoted by ▼. **d** Gel permeation chromatograms (GPC) of solid fractions from the same PE hydrogenolysis experiments. Number-average alkane product chain lengths are shown above the respective traces. The GPC calibration range is denoted by black lines.

Linear polyethylene (PE) homopolymer, isotactic polypropylene (*i*-PP), polyethylene-*co*−1-octene (PECO), and a post-consumer high-density polyethylene (HDPE) blue-dyed food container cap were initially investigated. Thus, 1.5 g of PE was heated with AlS/ZrNp_2_ (0.03 mol%) at 200 °C under 2.0 atm H_2_ (Fig. 2, Eq. (1)). Remarkably, in <48 min, the starting PE is entirely consumed and converted into liquid and volatile products. To our knowledge, this is the most rapid catalytic PE hydrogenolysis process reported to date in the open literature and is surprising considering the low catalyst loading, mild reaction conditions, and simple reactor setup (high-pressure glass flask + magnetic stirring bar). The reaction is extremely fast, with a calculated activity of ~4000 mol(CH_2_ units) mol(Zr)^−1^ h^−1^ (Table 1, Entry 1).

**Table 1:** Polyolefin and *n*-Hexadecane (C16) hydrogenolysis data over AlS/ZrNp_2_^a^

Entry	Substrate	Temperature (°C)	H_2_ pressure (atm)	Reaction time (min)	Catalyst loading^b^ (Zr mol%)	Substrate conversion (%)	Activity (h^−1^)^c^
1	PE	200	2.0	48	0.03	>99(1)^d^	4014(54)^e^
2	PE	150	2.0	30	0.03	32(1)^d^	2088(28)^e^
3	PE	150	2.0	120	0.03	95(1)^d^	1566(22)^e^
4^f^	PE	150	1.0	120	0.29	25^f 18^	43
5	*i*-PP	190	2.0	60	0.04	96(2)^d^	2193(40)^e^
6	PECO	190	2.0	60	0.04	>99(2)^d^	2995(52)^e^
7	HDPE food container cap	200	2.0	80	0.02	45(2)^d^	1520(29)^e^
8	C16	150	2.5	18	0.05	>99	690
9	C16	90	2.0	90	0.02	15(1)	56(7)^e^
10^g^	C16	150	2.0	1440	0.00^e^	0	0
11^h^	C16	150	2.0	30	0.01	Trace^i^	Trace
12	C16	120	0.5	15	0.02	12(1)	267(20)^j^
13	C16	120	4.0	15	0.02	12(1)	261(20)^j^

^a^Reaction in 350 mL heavy-walled glass pressure vessels.

^b^Catalyst loading with respect to CH_2_ units for all substrates. All saturated polyolefins are principally comprised of CH_2_ units, thus all polyolefin hydrogenolysis activities and catalyst loadings are reported with respect to CH_2_ units.

^c^Polyolefin hydrogenolysis activity: mol(CH_2_ units of CH_2_Cl_2_-soluble hydrocarbons + volatiles) mol(Zr)^−1^ h^−1^; C16 activity: mol(C_16_) mol(Zr)^−1^ h^−1^.

^d^Uncertainty in activity estimated from analytical uncertainty in percent conversion.

^e^Polyolefin conversion = yield of combined volatiles + CH_2_Cl_2_-soluble hydrocarbons; uncertainty in polymer hydrogenolysis activity and conversion reflects an estimated maximum of 20 mg solids loss during reaction workup; see SI for details regarding the estimation of uncertainties.

^f^Conversion was defined as the amount of C1–C9 hydrocarbons produced as a percentage of initial polymer mass.

^g^Control experiment without Zr.

^h^Hydrogenolysis reaction catalyzed by Cp*Zr(CH_3_)_3_ supported on AlS^38^.

^i^Trace C13–C15 hydrocarbons not attributable to starting C16 detected in GC–MS.

^j^Uncertainty in activity and conversion is ±3 standard deviations of all activities in varied pressure experiments.

Polyolefin hydrogenolysis products were analyzed as follows. At the end of the reaction, the reactor is cooled to 25 °C in a water bath, and the reactor headspace contents analyzed by GC/FID. Next, the remaining volatiles are vented, the residual liquid + higher molecular mass solid saturated hydrocarbon products are stirred with DCM, and the resulting suspension is filtered. The reactor components are then rinsed several times with DCM to ensure the collection of all solid and DCM-soluble liquid products. The filtrate is collected, the DCM is evaporated under ~1 Torr reduced pressure, and the resulting hydrocarbons are weighed. The mass of this product gives the yield of the DCM extract fraction, which is analyzed by GC/MS (gas chromatography/mass spectrometry). We find that the DCM extraction and evaporation under reduced pressure results in <5 wt% loss of the volatile liquid hydrocarbons. After isolation via filtration, the DCM-insoluble higher molecular mass saturated hydrocarbon fraction is collected, dried, and weighed. The mass of this fraction, corrected for the weight of the catalyst, gives the yield of the solids fraction. The solid product contents are then analyzed by gel permeation chromatography (GPC). The volatiles fraction mass is the mass not accounted for after measuring the solids and DCM extract fractions, and its composition is readily analyzed via headspace gas chromatography/flame ionization detection (GC/FID). The combined mass of the volatiles and DCM extract fractions as a percentage of the initial reactant polyolefin mass is taken as the percent conversion for a given polyolefin hydrogenolysis reaction. These analytical methods for handling polyolefin hydrogenolysis products are similar to those used in the recent polyolefin hydrogenolysis literature^12,14,28^.

To analyze the reaction products in more detail, the reaction was slowed by decreasing the temperature to 150 °C and monitored at varied time intervals, with reactions halted periodically by cooling to 25 °C (Eq. (1)). The time dependence of PE hydrogenolysis with 0.03 mol% Zr is shown in Fig. 2a and Table 1, entries 2 and 3. After only 10 min at 150 °C, a 16% yield of volatile and DCM-soluble products is obtained (Fig. 2a–c), and the compositions were analyzed by GC/MS as noted above. For the starting PE, the number average molecular mass (determined using GPC) of the starting PE (*M*_n_ = 11.62 kg mol^−1^) decreased by 33% to *M*_n_ = 7.8 kg mol^−1^ (Fig. 2d). Next, 30 min heating afforded a 32% yield of volatile and liquid products, and PE with *M*_n_ = 4.4 kg mol^−1^ (Table 1, Entry 2). After 50 min and 2 h (Table 1, Entry 3), the volatile and liquid products were obtained in 86% and 96% yields, respectively. The PE solids comprise only a minor fraction of the products with *M*_n_ = 2.5 kg mol^−1^ (for 50 min) and *M*_n_ < 0.86 kg mol^−1^ (for 2 h) (Fig. 2d). Note also the gradual decline in the most probable chain length of the DCM extracts with conversion, falling from ~18 carbons (10 min) to 14 carbons (30 min), 12 carbons (50 min), and 11 carbons (2 h) (Fig. 2c). We hypothesize that minor alkane branching observed in hydrogenolysis products of linear PE may be caused by the reinsertion of eliminated olefin. (vide infra). Note that PE hydrogenolysis catalyzed by ZrNp_4_ supported on silica-alumina proceeds at ~3% of the rate of the present reactions catalyzed by AlS/ZrNp_2_ (Table 1, Entry 4).

**Fig. 3:**
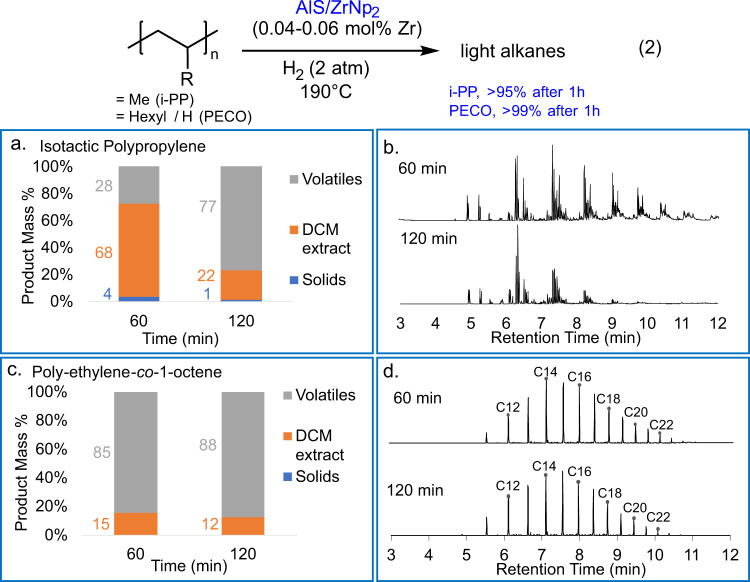
Catalytic hydrogenolysis of branched polyolefins. **a** Temporal product distributions for the AlS/ZrNp_2_-catalyzed i-PP hydrogenolysis (190 °C, 2.0 atm H_2_ 0.04 mol% Zr). **b** GC/MS chromatograms of DCM extracts from i-PP experiments. **c** Temporal product distributions for AlS/ZrNp_2_-catalyzed PECO hydrogenolysis (190 °C, 2.0 atm H_2_, 0.04 mol% Zr). **d** GC/MS chromatograms of DCM extracts from PECO experiments.

While linear PE homopolymers are an informative substrate due to their relatively low melting points and simple structures, *i*-PP and PECO are of greater technological significance^29^. The AlS/ZrNp_2_ catalyst was next applied to these materials at 190 °C to lower the melt viscosity for ease of stirring (Fig. 3, Eq. (2)). Reacting 1.0 g i-PP (*M*_n_ = 36 kg mol^−1^) over 0.04 mol% AlS/ZrNp_2_ under 2 atm H_2_ effects quantitative conversion to 68% low-molecular-weight (*M*_w_ < C30) products and 28% C1–C6 volatiles in only 1 h (Table 1, entry 5, Fig. 3a, b). The i-PP DCM extract GC/MS chromatogram has greater complexity than that of PE since the product alkanes are not only linear and have significant ethyl- and methyl-branching, reflecting the different PP chain cleavage points (Fig. 3c). The DCM extract number average molecular mass (*M*_n_) also falls with increasing conversion, and the distribution narrows as for PE (Fig. 3c). Stirring PECO (*M*_n_ = 7.0 kg mol^−1^, 2.5% 1-octene incorporation) reaction mixtures was challenged by the substantial melt viscosity. Nevertheless, hydrogenolysis proceeds rapidly to afford 85% volatile and 15% DCM soluble/low-M_w_ alkane yields within 1 h (Fig. 3b). The 1 and 2 h GC/MS data are similar, probably reflecting H_2_ starvation and yielding a most probable chain length of ~15 carbons (Fig. 3d). Low-level alkane branching (~1%) is present, probably from the enchained 1-octene comonomer. Comparable hydrogenolysis rates with post-consumer HDPE (*M*_n_ = 9.9 kg mol^−1^) are achieved at 200 °C/2.0 atm H_2_/0.02 mol% Zr loading in 80 min (Table 1, Entry 7). Furthermore, note that the deliberate introduction of ~5 mL of ambient air (1:1 O_2_:Zr ratio) into the reaction headspace prior to beginning the reaction has no measurable effect on catalyst activity (SI p. S5).

### Kinetic studies with a model saturated hydrocarbon

**Fig. 4:**
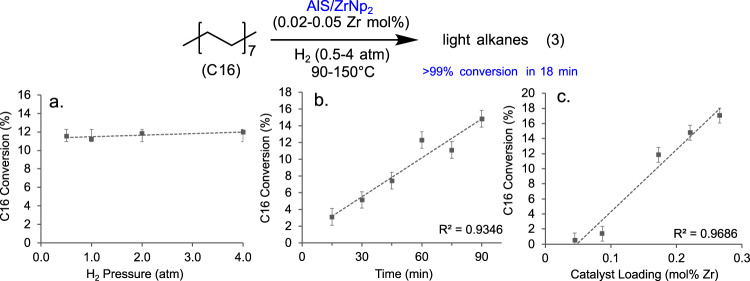
Hexadecane (C16) hydrogenolysis data over AlS/ZrNp_2_ as a function of reaction parameters. Conditions for all reactions: 120 °C, 0.02 mol% Zr, 15 min, 2.0 atm H_2_ (350 mL vessel), unless otherwise stated. **a** Conversion as a function of H_2_ pressure. Error bars represent 3 standard deviations. **b** Conversion as a function of reaction time. The rapidity of the reaction constrained the temperature to 90 °C for accurate measurements. **c** Conversion as a function of catalyst loading. Error bars are ±1% in C16 conversion (absolute) to account for analytical uncertainty in the plots in (**b**) and (**c**).

Liquid *n*-hexadecane (C16; b.p. = 287 °C) served as a realistic PE reactivity/rheology model. The C16 conversion is defined as the mass of C16 consumed over the course of a given reaction. This is quantified via GC/MS. For polyolefins, it is defined as the mass of volatile and DCM-soluble hydrocarbons produced. Measurements as a function of stirring rate showed that stirring at ≥600 RPM minimizes mass transport effects (Fig. S8). For example, 1.93 mL of C16 over AlS/ZrNp_2_ (0.05 mol% Zr) at 150 °C/2.5 atm H_2_ (350 mL vessel) is completely converted to C1–C15 hydrocarbons in as little as 18 min as assessed by GC/MS and GC/FID (Eq. (3), Table 1, Entry 8; Video S1). Similar to the PE hydrogenolyses, the products are linear even and odd carbon number hydrocarbons, with ~1% methyl branched alkanes (Figs. S12–S14). Interestingly, the AlS/ZrNp_2_-catalyzed hydrogenolysis of C16 begins at temperatures as low as 90 °C (Table 1, Entry 9). A 24 h control with only C16 + AlS + H_2_ yields negligible hydrogenolysis products (Table 1, entry 10, Fig. S9). The above results argue that C16 is a valid PE model. Hydrogenolysis of C16 catalyzed by Cp*Zr(CH_3_)_3_ supported on AlS (Fig. 1b) affords only trace conversion to lighter alkanes, suggesting that under these conditions, the choice of Zr ligand has a marked influence on the catalyst activity (Table 1, entry 11). All kinetic studies were carried out at low conversions (<15%) to minimize H_2_ starvation and small hydrocarbon intrusion effects. Varying the H_2_ concentration in the reaction by changing the H_2_ pressure (0.5–4.0 atm) indicates minimal conversion dependence on H_2_ concentration, implying near zero-order kinetics in [H_2_] under these conditions (Table 1, entries 12 and 13, Fig. 4a). Note that the estimated H_2_ pressure drop is negligible, even at 0.5 atm initial H_2_ pressure; at 11% conversion, the estimated partial pressure drop of H_2_ at the end of the reaction is ca. 3%. Monitoring the consumption of C16 over time at low conversions (<15%) reveals a linear dependence also suggestive of effective pseudo-zero order behavior in [C16] (Fig. 4b). Furthermore, increasing the catalyst loading produces a linearly scaled increase in conversion for a fixed time (15 min), implying a near first-order in [Zr] behavior (Fig. 4c). That the plotted trendline does not pass exactly through zero may reflect trace impurity catalyst deactivation at very low loadings, but otherwise yields an empirical rate law for hexadecane hydrogenolysis of *ν* ∼ *k*[Zr]^1^[H_2_]^0^[C16]^0^. To our knowledge, this is the first kinetic/mechanistic study of cationic d^0^ catalyst-mediated liquid alkane hydrogenolysis.

### Catalyst structural and reaction mechanism characterization

**Fig. 5:**
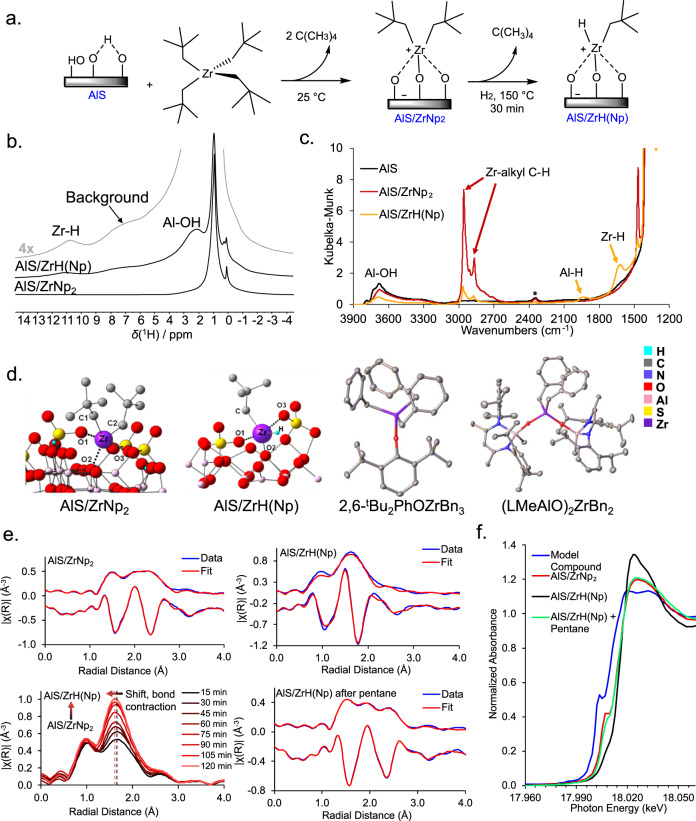
Characterization of the AlS/ZrNp_2_ adsorbate and AlS/ZrH(Np) hydrogenolysis product; atomistic models of Zr dialkyl and alkyl hydride structures on AlS vs. neutrally charged model Zr dialkyls. **a** Zr(neopentyl)_4_ chemisorption on very Brønsted acidic sulfated alumina (AlS), and hydride generation via Zr-neopentyl hydrogenolysis. **b** Solid-state ^1^H MAS-NMR spectra of AlS/ZrNp_2_ and AlS/ZrH(Np). **c** DRIFTS vibrational spectra of AlS/ZrNp_2_, AlS/ZrH(Np), and the AlS support. *Atmospheric CO_2_ background artifact. **d** DFT-computed structures of AlS/ZrNp_2_, AlS/ZrH(Np), and single-crystal diffraction structures of two 4-coordinate Zr models. **e** EXAFS spectra of AlS/ZrNp_2_, AlS/ZrH(Np), and AlS/ZrH(Np) after pentane exposure (AlS/Zr(alkyl)_2_), and stacked *operando* EXAFS temporal plot for AlS/ZrNp_2_ hydrogenolysis at 25 °C. The real component of EXAFS spectra is offset by −0.3 Å^−3^. **f** Zr K-edge XANES of AlS/ZrH(Np), AlS/ZrNp_2_, AlS/Zr(alkyl)_2_, and 2,6-^t^Bu_2_PhOZr(benzyl)_3_.

Using the aforementioned procedures, AlS/ZrNp_2_ with a Zr loading of 1.40 wt% (~0.5Zr/nm^2^) was prepared by rigorously anaerobic Zr(neopentyl)_4_ chemisorption on AlS (Fig. 5a). Solid-state ^1^H magic angle spinning (MAS) NMR spectroscopy reveals a δ 0.90 ppm signal assignable to Zr–Np CH_3_ and CH_2_ moieties (Fig. 5b). Interestingly, upon H_2_ exposure at 150 °C/30 min, one or more neopentyl ligands are hydrogenolyzed, yielding HNp and a proposed Zr–H species, AlS/ZrH(Np) (Fig. 5a, b) with a characteristic δ11 ppm ^1^H Zr–H NMR signal^30–32^, not present in D_2_ reactions (Fig. S4). The rapidity of this hydrogenolysis was also verified by gas-phase ^1^H NMR monitoring of the AlS/ZrNp_2_ + H_2_ reaction (SI p. S16 and Fig. S11). Besides weakened C–H alkyl signals, resonances at δ2.0 and δ7.5 ppm are also visible and tentatively assigned to Al–OH and Al–H groups, respectively^33,34^. DRIFTS vibrational spectra reveal υZr–H and υAl–H stretching modes at 1620 and 1930 cm^−1^, respectively (Fig. 5c)^18,30^, not present in AlS nor in AlS/ZrNp_2_ (Fig. 5c). Furthermore, exposing AlS/ZrNp_2_ to D_2_ significantly weakens these features, supporting the υZr–H and υAl–H assignments (Fig. S5). Additionally, the AlS/ZrNp_2_ 3000–2800 cm^−1^ alkyl υC–H modes diminish in intensity upon H_2_ exposure, supporting Zr-neopentyl → AlS/ZrH(Np) conversion (Fig. 5c). Interestingly, on exposing AlS/ZrH(Np) to pentane vapor, the Zr–H δ11 ppm NMR signal and the 1620 cm^−1^ vibration vanish, however, the signals at δ7.5 ppm and 1930 cm^−1^ remain (Fig. S6), arguing the latter represent less reactive Al–H species.

Zr EXAFS and DFT computation provide additional chemical and electronic structural information. The former indicates that AlS/ZrNp_2_ is an oxide-bound ZrNp_2_ species having three rather long Zr–O bonds (~2.26 Å average) and two Zr–C bonds (Fig. 5d, f; Table 2, entry 1). The DFT-derived model for AlS/ZrNp_2_ (Fig. 5d) reveals three 2.18–2.24 Å Zr–O bonds in excellent agreement with the EXAFS data (Table 2, entry 1). *Operando* EXAFS monitoring of AlS/ZrNp_2_ hydrogenolysis reveals gradual conversion to AlS/ZrH(Np), with the Zr–C bond number falling from 2.0 (AlS/ZrNp_2_) to 1.0 (AlS/ZrH(Np)) and a slight average Zr–O bond length contraction to ~2.19 Å (Fig. 5d, e; Table 1, entry 2), consistent with the less encumbered hydride ligand replacing Np. Further H_2_ treatment at 150 °C affects minimal change, demonstrating that AlS/ZrH(Np) is stable under catalytic conditions. From the DFT analysis, the three AlS/ZrH(Np) Zr–O bonds are slightly contracted to 2.10–2.20 Å, in accordance with the EXAFS data. Additionally, the DFT-computed 1704 cm^−1^ AlS/ZrH(Np) υZr-H frequency compares favorably with the 1620 cm^−1^ DRIFTS value, further supporting the structure in Fig. 5d (Table 2, entry 2). AlS/ZrH(Np) pentane exposure affords an AlS/Zr(alkyl)_2_ species having three Zr–O bonds and two Zr–C bonds (Fig. 5d, see SI), in agreement with the NMR and DRIFTS data (Fig. S6). Note that the AlS/ZrNp_2_ and AlS/ZrH(Np) Zr–O bonds are significantly elongated vs. those in neutrally charged 2,6-^t^Bu_2_PhOZr(benzyl)_3_ (Zr–O = 1.94 Å)^35^ and (L(Me)AlO)_2_Zr(benzyl)_2_ (L = (2,6-^i^Pr_2_C_6_H_3_NC(Me))_2_CH) (Zr–O = 1.91 Å; Fig. 5d, Table 2, entries 3, 4)^36^, and for formally neutrally charged SiO_2_/Zr–H (1.95 Å; Table 2, entry 5)^37^, with shorter Zr–O bonds suggesting more covalent σ-bonding, and longer Zr–O bonds greater electrostatic character between the electron-deficient Zr centers and weakly basic AlS oxide ligands^23,38,39^. Also, the XANES Zr K-edge energies for AlS/ZrNp_2_ (18.005 keV), AlS/ZrH(Np) (18.008 keV), and AlS/Zr(alkyl)_2_ (18.006 keV) lie in the range of cationic complexes vs. neutrally charged ^t^Bu_2_PhOZr(benzyl)_3_ (17.998 keV) and Zr(benzyl)_4_ (17.999 keV) (Fig. 5f)^22^. Finally, AlS/ZrH(Np) charge partition analysis computation^40^ reveals that the Zr atom in model (EtO)_2_Zr(neopentyl)_2_ bears a +1.66 charge vs. +1.99 in AlS/ZrH(Np). Calculated Zr–O distances are, (EtO)_2_Zr(neopentyl)_2_, 1.93 Å, and AlS/ZrH(Np), 2.10–2.20 Å.

**Table 2:** Experimental EXAFS and DRIFTS structural data for AlS/ZrNp_2_ and AlS/ZrH(Np), and their DFT computed metrical parameters

Entry	Species	Bond	Experimental bond length (Å)	DFT bond length (Å)	DRIFTS stretching freq. *υ* (cm^−1^)	DFT stretching freq. *υ* (cm^−1^)
**1**	**AlS/ZrNp**_**2**_	Zr–O1	2.26(2)^a^	2.24	**Alkyl C–H**	**Alkyl C–H**
		Zr–O2	2.26(2)^a^	2.20	2958	3041
		Zr–O3	2.26(2)^a^	2.18	2868	2679
		Zr–C1	2.42(3)^a^	2.12		
		Zr–C2	2.42(3)^a^	2.22		
**2**	**AlS/ZrH(Np)**	Zr–O1	2.29(3)^a^	2.20	**Alkyl C–H**	**Alkyl C–H**
		Zr–O2	2.14(3)^a^	2.10	2963	3040
		Zr–O3	2.14(3)^a^	2.15	2870	2966
		Zr–C	2.38(3)^a^	2.20	**Zr–H**	**Zr–H**
		Zr–H	N.D.	1.83	1620	1704
**3**	**2,6-Bu**_**2**_**PhOZrBn**_**3**_	Zr–O	1.947(1)^b^	N.D.	N.D.	N.D.
		Zr–C	2.279(2)^b,c^			
		Zr–O	1.95(3)^a^			
		Zr–C	2.26(3)^a,c^			
**4**^d^	**(LMeAlO)**_**2**_**ZrBn**_**2**_	Zr–O	1.9132(14)^b^	N.D.	N.D.	N.D.
		Zr–O	1.9143(14)^b^			
		Zr–C	2.265(2)^b^			
		Zr–C	2.289(2)^b^			
**5**^e^	**SiO**_**2**_**/ZrH**	Zr–O	1.94^c^	N.D.	**Zr–H** 1638	**Zr–H** N.A.

^a^Bond lengths from EXAFS.

^b^Bond lengths from single-crystal X-ray diffraction.

^c^Average of 3 bond lengths.

^d^Reference 35.

^e^Reference 37.

## Discussion

### Reaction mechanism

**Fig. 6:**
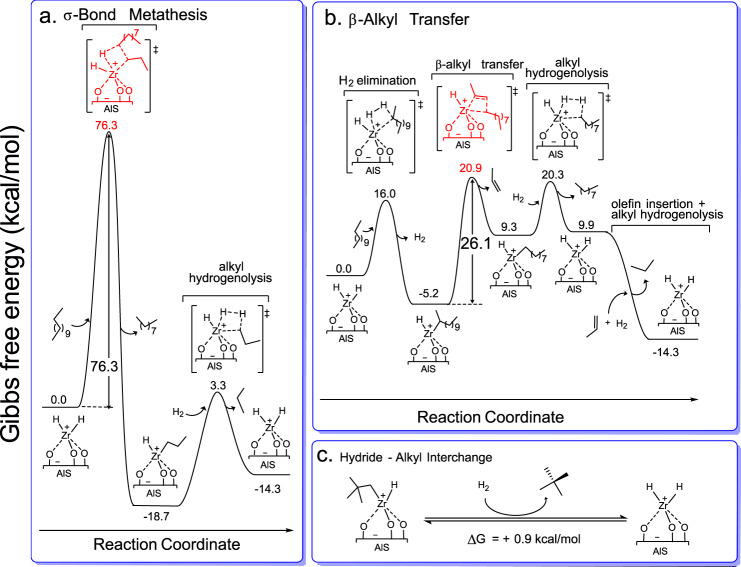
Computed reaction coordinates for AlS/ZrH_2_-catalyzed *n*-dodecane hydrogenolysis via plausible turnover-limiting pathways. **a** C–C scission via four-center σ-bond metathesis. **b** C–C scission via intramolecular *ß*-alkyl transfer. **c** Representative computed energetics for Zr alkyl/hydride ligand interchange.

Early transition metal d^0^ reaction pathways differ distinctly from those of most later metal systems and frequently involve combinations of four-center σ-bond metathesis and/or C = C/X = X insertion/extrusion. For the challenging cleavage of polyolefin C–C bonds, as mediated by the present very unusual surface catalysts, two turnover-limiting scenarios were examined: (1) σ-bond metathesis^26,27^ and (2) *ß*-alkyl transfer^18,41,42^. From the present empirical rate law, *ν* = *k*[Zr]^1^[H_2_]^0^[C16]^0^, with [C16] in large excess, we infer that the turnover-limiting step or any preceding steps in rapid equilibrium do not involve direct H_2_ attack at the catalytic center. From the adsorbate structures, kinetic data, control experiments, and literature precedent, DFT reaction coordinates were computed for scenarios (1) and (2) using *n*-dodecane as a model, in Fig. 6a, b, respectively^19,20^. As for catalyst choice, note that a multitude of AlS/ZrH(R) species (R = alkyl or H) of similar energies are doubtless rapidly equilibrating via C–H σ-bond metathesis processes (Fig. 6c). AlS/ZrH_2_ was selected for simplicity; however, similar pathways are conceivable for other AlS/ZrH(R) species. For C–C scission via σ-bond metathesis (Fig. 6a), this pathway has a remarkably prohibitive 76 kcal/mol computed barrier. Indeed, an experiment with a 1:1 ratio of ethane and H_2_ (1 atm total pressure) over a relatively high AlS/ZrH(Np) loading (0.9% mol Zr) reveals negligible hydrogenolysis at 150 °C/1 h (Eq. (4)).

In contrast, a Zr-*sec*-dodecyl complex is readily accessed via C–H σ-bond activation/metathesis of *n*-dodecane (H_2_ elimination step in Fig. 6b) and is the lowest energy intermediate found on the reaction coordinate (catalyst resting state), in agreement with the experimental zero-order rate law dependence on alkane concentration. Primary or secondary C–H activation is comparably exergonic with Δ*G* ≈ −5 kcal/mol. Secondary activation on a polyolefin chain seems statistically more probable, with the intermediate then undergoing intramolecular *ß*-alkyl transfer, yielding a Zr-alkyl and olefin (Fig. 6b; Δ*G* = +14.5 kcal/mol). Here Δ*G*^‡^ = 26.1 kcal/mol barrier and is rate-limiting for chain shortening. In principle, this process is reversible, and the olefin can reinsert, explaining the product composition of ~1% methyl branches. The next step in this sequence is Zr–C bond hydrogenolysis with a computed 11.0 kcal/mol barrier to yield a Zr dihydride. This step is slightly endergonic (Δ*G* = 0.6 kcal/mol) and yields shorter alkanes. Note that the barrier for Zr–C bond hydrogenolysis is slightly lower than the *ß*-alkyl transfer step and therefore is not expected to be rate-limiting, in agreement with the experimental zero-order rate law dependence on H_2_ concentration. Experimentally, alkenes are not detected at any stage in the reaction, and the DFT modeling shows that any alkene produced from *ß*-alkyl transfer rapidly inserts into a Zr–H bond in a barrierless, strongly exergonic step (Δ*G* = −24.2 kcal/mol), and the product is then hydrogenolyzed. Therefore, overall alkane hydrogenolysis is computed to be exergonic by Δ*G* = −14.3 kcal/mol. While this reverse of single-site polymerization producing smaller alkenes from a longer polyalkane chain is endergonic, the coupled olefin hydrogenation renders the overall alkane, and by inference, polyethylene deconstruction, decidedly exergonic. Note that identifying AlS/ZrH(Np)-catalyzed *β*-alkyl transfer as the key turnover-limiting step is consistent with the experiment and has implications for other polymers and other electrophilic transition metal catalysts.

In conclusion, an earth-abundant single-site formally cationic Zr-alkyl/hydride catalyst was synthesized on highly Brønsted acidic sulfated alumina and characterized by solid-state ^1^H MAS and ^13^C CPMAS-NMR, DRIFTS, ICP/AES, XANES, EXAFS, DFT, and evaluated for polyolefin and hexadecane (C16) hydrogenolysis. This catalyst mediates very rapidly (690 mol C16 mol Zr^−1^ h^−1^) hexadecane hydrogenolysis under relatively mild conditions (18 min, 150 °C/2.5 atm H_2_). Under similar solventless conditions, polyethylene, polyethylene-*co*−1-octene, isotactic polypropylene, and commercial post-consumer polyethylene are rapidly hydrogenolyzed to low molecular mass hydrocarbons under mild conditions (150–200 °C/2 atm H_2_) at low catalyst loadings (0.02–0.04 mol% Zr). Experimental results combined with DFT computation reveal that the turnover-limiting step in alkane/polyolefin C–C scission/chain shortening is intramolecular *ß*-alkyl transfer, in contrast to the σ-bond metathesis process common in much early transition metal catalytic chemistry. The catalytic species, a supported electrophilic Zr-hydride, is readily formed during the hydrogenolysis process. These results convey implications for deconstructing other polymers and inventing new catalysts to achieve this.

## Methods

All procedures for air- and moisture-sensitive compounds were carried out with rigorous exclusion of O_2_ and moisture in flame- or oven-dried Schlenk-type glassware interfaced to a high-vacuum (10^−5^–10^−6^ Torr) line or in an argon-filled M-Braun glovebox with a high capacity recirculator (<1 ppm O_2_). Argon used on high-vacuum lines (Airgas, UHP-grade) was purified by passage through MnO/vermiculite and activated Davidson 4 Å molecular sieve columns. All were solvents dispensed from activated alumina/CuO columns prior to use. *n-*pentane (Sigma) was further purified by drying over Na/K alloy followed by passage through a fiberglass filter in an argon glovebox. Aluminum oxide was purchased from Nanostructured and Amorphous Materials (gamma, nanopowder 20–30 nm). Sulfuric acid (98%) was purchased from Fisher. *n*-hexadecane (C16) was purchased from Sigma and was purified by heating at 120 °C over Na for 48 h, followed by degassing at room temperature, and was further purified by passage through a 0.22 µm PTFE syringe filter immediately prior to use. All components containing plastic directly contacting C16 prior to hydrogenolysis experiments (i.e., syringes, syringe filters, needles, Teflon reactor caps) were pumped in the glove box inlet chamber overnight prior to use. Oxygen (UHP grade) used for calcination was purchased from Airgas and used without further purification. Deuterium (Sigma) and hydrogen (Airgas, UHP) were purified by passage through an oxygen/moisture trap (Matheson, model MTRP-0042-XX). Zirconium(IV) chloride and neopentylmagnesium chloride (1.0 M solution in Et_2_O) were purchased from Sigma and used without further purification. Tetrakis(neopentyl)zirconium (ZrNp_4_) was synthesized according to a modification of a literature procedure^43^. In a typical synthesis, neopentlymagnesium chloride (4.4 mmol) was added dropwise to a suspension of ZrCl_4_ (1.0 mmol) in ether at −78 °C. The mixture was allowed to stir for 2 h, slowly warmed to room temperature, then stirred for an additional 2 h at room temperature. The solvent was removed in vacuo, and the resulting solids were extracted with pentane. The pentane filtrate was isolated, and the solvent was removed in vacuo to yield a colorless solid. The crude product was purified by sublimation at 70 °C and ~10^−6^ Torr, yielding a colorless microcrystalline solid with 83% yield. Polymers were dried in the melt (130–165 °C) under a high vacuum for 48 h before use in the hydrogenolysis reactions.

### Physical and analytical measurements

Inductively coupled plasma (ICP) analysis was performed by Galbraith Laboratories Inc., Knoxville, TN, USA. ^1^H (500 MHz) and ^13^C (125 MHz) NMR spectra of hydrogenolysis products were obtained with a Bruker Avance III system equipped with a DCH Cryoprobe. ^1^H MAS (400 MHz) and ^13^C CP-MAS (100 MHz) Solid State NMR measurements were obtained with a Bruker Avance III system equipped with a 4 mm Bruker HX probe. The rotor was charged with the sample in an Ar glove box. The rotor speed was set to 14 kHz for all spectra. Gas chromatography/mass spectrometry (GC/MS) analysis of hydrogenolysis product mixtures was carried out on an Agilent GCMSD equipped with a DB5 column (oven program: 1. 2 min. 50 °C hold 2. 30 °C/min ramp 3. 2 min hold at 300 °C). Split mode injection at 2 µL/injection and a 100:1 split ratio was used. For GC–MS quantification of *n*-hexadecane, a four-point calibration (0.1, 0.2, 0.3, 0.4 mg/mL) was carried out for each group of samples analyzed by GC/MS with a target sample concentration of ~0.2 mg/mL. Calibration standards were prepared in 1,1,2,2-tetrachloroethane (TCE) and stored in airtight Teflon-valved glassware. Diffuse reflectance infrared spectroscopy (DRIFTS) measurements were taken under Ar on Thermo 6700 infrared spectrometer equipped with a Harrick Praying Mantis DRIFTS attachment. ZnS windows were used for the DRIFTS cell. Anhydrous KBr with an Ar glovebox atmosphere in the cell was used as a background. BET surface area measurements were carried out with a Micromeritics 3Flex Surface Characterization Analyzer.

### X-ray absorption spectroscopy

X-ray absorption near edge structure (XANES) and extended X-ray absorption fine structure (EXAFS) measurements at Zr K-edge (17,998 eV) were performed at the 5 BM-D beamline of the DND-CAT at the Advanced Photon Source. A double Si (111) monochromator was used for energy selection with an energy resolution of Δ*E*/*E* = 1.4 × 10^−4^. The X-ray energy was calibrated using a metallic Zr foil. The incident X-ray intensity was measured by a spectroscopy-grade ionization chamber (FMB-Oxford) filled with 600 He/100 N_2_ (Torr) and was detuned to 60% of its maximum for harmonic rejection. EXAFS spectra were collected in fluorescence mode using a passivated implanted planar silicon (PIPS) detector (Canberra). The sample and the detector were positioned 45° and 90°, respectively, to the X-ray beam direction. Energy scans were executed from 250 eV below to 550 eV above the Zr K edge, which produces the EXAFS spectra. The catalyst AlS/ZrNp_2_ with a Zr loading of 1.40 wt% was pressed into a sample holder for the EXAFS measurements. The samples were sealed airtight in a THMS600 Linkam cell inside the glove box, which was pressured with ultrapure Ar gas. Positive Ar pressure was maintained throughout the measurement. After measuring the AlS/ZrNp_2_ sample, in-situ catalyst hydrogenolysis was carried out by flowing H_2_ gas with a flow rate of 50 sccm at room temperature with EXAFS data collected every 15 min for 2 h until no further changes were observed. This was followed by heating the sample to 150 °C with continued H_2_ flow. Data were collected every 15 min for up to 2 h. No changes were observed after this step of the treatment. There was a change in the structure during the hydrogenolysis reaction at room temperature, and these data are shown in the manuscript (Fig. 5e). XANES data extraction, normalization, and background subtraction was performed using Demeter:Athena. EXAFS data analysis was carried out using the software Demeter:Artemis. The bond lengths (*R*) and coordination number (*N*) were obtained by a least-square fit in the *R*-space of the nearest neighbors using *k*_2_-weighted Fourier transform fitting parameter. For data analyses, a standard reference model compound was used: powder 2,6-^t^Bu_2_PhOZrBn_3_, which was measured in fluorescence mode.

### Computational details

DFT-based simulations were performed with the/Quickstep package, using a hybrid Gaussian and plane wave method^44^. A double-quality DZVP Gaussian basis set was employed for the Al and Zr atoms, and a triple-quality TZVP Gaussian basis set was employed for all the other atoms^45^. The Goedecker–Teter–Hutter pseudopotentials^46^ together with a 400 Ry plane wave cutoff were used to expand the densities obtained with the Perdew–Burke–Ernzerhof (PBE)^47^ exchange-correlation density functional, and vdW forces are taken in account with the Grimme D3 Method^48^. Only the gamma point was considered in a supercell approach. Periodic boundary conditions are applied in all directions of space. Molecular graphics were produced by the CHEMCRAFT graphical package^49^. Enthalpic and entropic contributions along the reaction pathway were evaluated by performing the frequency calculation of the molecular species at 298.15 K and 1 atm as implemented in the G16 code^50^. In this context, adsorbed catalysts were modeled by simple molecular species, and only the entropic contribution related to vibrational motion is considered. G16 calculations were performed at the level of the B3LYP hybrid functional. The standard all-electron 6–311G** basis set was used for all atoms. The enthalpic and entropic contributions were then “appended” to the SCF energy profile to obtain the Gibbs free energy profile.

### General hexadecane hydrogenolysis procedure

In the glovebox, C16 (typically 0.50 g) was passed through a 0.22 µm PTFE syringe filter directly into a dry heavy-walled glass pressure reactor (350 mL volume) containing a 10 mm ovoid stir bar. The desired amount of AlS/ZrNp_2_ (typically 25 mg, 0.02 mol% Zr) was added to the reactor and was sealed with a threaded Teflon cap with an NPT valve installed. The vessel was carefully removed from the glovebox and interfaced with a high-pressure/high-vacuum line. The reactor was degassed at room temperature (30 s), then charged to the desired pressure of H_2_. The reactor was placed in an oil bath set to the desired reaction temperature. Once the oil bath thermocouple reached reaction temperature, the time interval was started. At the end of the time interval, the reactor was removed from the oil bath, then cooled via a water bath to room temperature. Headspace samples were taken, if needed, at this point. Note, for determining reaction conversion as a function of time, the reaction temperature was decreased to 90 °C to keep reaction conversions below 15% and preserve the accuracy of the apparent reaction rate. The reactor was vented through the NPT valve and opened to air. Approx. 5 mL dichloromethane (DCM) or TCE was used to wash the Teflon cap and the interior of the NPT valve. These washings were added to the reactor. The washings were transferred to a syringe equipped with a 0.22 µm PTFE filter. The reactor was washed 4× with ~10 mL portions of clean solvent, with each washing being added to the syringe. The washings were passed through the filter directly into a 100 mL volumetric flask. The filter was washed 4× with ~5 mL portions of clean solvent, with washings being added to the volumetric flask. Pipettes used to transfer solvent solutions were also washed with clean solvent and added to the volumetric flask. The solution was diluted to the calibration mark, then diluted further using standard analytical techniques to ~0.2 mg/mL. A four-point external calibration (~0.1, 0.2, 0.3, and 0.4 mg/mL) of hexadecane in TCE was used to determine the concentration of the sample via GC/MS. The series of external calibration standards were run for each round of reaction sample measurements. Uncertainty in kinetic measurements was taken to be ±1% (absolute) to account for analytical uncertainty, except in the case of varied H_2_ pressure experiments, where uncertainty was taken to be 3 standard deviations of the data set.

### General polyolefin hydrogenolysis procedure

In the glovebox, AlS/ZrNp_2_ and the desired amount of polymer were loaded into a dry heavy-walled glass pressure reactor (350 mL volume) containing a 10 mm ovoid stir bar, typically with ~15% mass loading of catalyst (0.02–0.04 mol% Zr). The easily implemented and versatile hydrogenolysis reactor and analytical procedures serve four functions: (i) for constant polymer and catalyst, to screen products as a function of reaction conditions such as temperature, H_2_ pressure, reaction time, and stirring rate. (ii) For constant polymer and reaction conditions, to screen different catalysts in terms of relative activity and product mix selectivity. (iii) For constant catalyst and reaction conditions, to screen different polymers in terms of relative activity and product mix selectivity. (iv) The optically transparent heavy-wall glass reactor can be filled in the glove box, is high-vacuum line compatible, and allows ready visualization of the reaction progress (solid → liquid → gas) as well as stirrer malfunction or possible coke formation (never observed). The polymers were prepared by shaving from a larger puck of pre-melted stock. The reactor was sealed with a threaded Teflon cap having an installed NPT valve. The vessel was carefully removed from the glovebox and interfaced with a high-pressure/high-vacuum line. The reactor was degassed at room temperature (30 s), then charged to 2 atm H_2_. The reactor was then placed in an oil bath set to the desired temperature (polyethylene 150 °C, polypropylene, polyethylene-*co*−1-octene, HDPE food container cap 190 or 200 °C). Once the polymer melted and contacted the catalyst in the melt, the time interval was started. Stirring was typically set to 300 rpm initially, then increased to ~800 rpm after a sufficient decrease in polymer viscosity. At the end of the time interval, the reactor was removed from the oil bath, then air-cooled to room temperature. Headspace samples were taken at this point. Headspace samples were collected by the expansion of the reactor contents into an evacuated 500 mL Teflon-valved glass bulb. Individual samples for analysis were taken via septum and gastight headspace syringe (1 mL). The reactor was vented through the NPT valve and opened to air. Approx. 5 mL DCM was used to wash the Teflon cap and the interior of the NPT valve. These washings were added to the reactor. Solids were suspended in the DCM washings and then filtered to isolate the solids. The reactor was washed enough times to remove all residue. The solids were washed ~3× with 5 mL portions of DCM, then dried at ~1 Torr overnight. This product was assigned the “solids fraction”. GPC analysis was carried out for this product fraction. The DCM washings were collected, and the DCM was removed under reduced pressure overnight (~1 Torr). These DCM-soluble hydrocarbons are assigned as “DCM extract”. Note that some volatile liquid hydrocarbons (C5–C7) are lost during DCM removal, however, we find that <5 wt% of the total product mass is lost. Approximately 10 mg of the DCM extract fraction was added to ~1 mL DCM for GC/MS analysis. Unaccounted-for mass is assigned to the “volatiles” fraction, and the presence of light hydrocarbons is confirmed via GC-FID. The percent conversion of a polyolefin hydrogenolysis reaction is defined as the mass of “volatiles” and “DCM extract” produced as a percentage of the initial polyolefin mass. Uncertainty in polymer hydrogenolysis products mass was estimated to be 20 mg (1–2% of initial polymer mass) and was carried over into calculating polymer hydrogenolysis activities.

### Synthesis of AlS/ZrH(Np), AlS/ZrD(Np) and AlS/Zr(alkyl)_2_ (Pentane-treated AlS/ZrH(Np))

In the glovebox, AlS/ZrNp_2_ (200 mg) was added to a dry 75 mL heavy-walled glass pressure reactor. The reactor was sealed, interfaced to a high pressure/high vacuum line, evacuated, then charged with 1 atm H_2_. The reactor was heated to 150 °C for 5 min, then evacuated. A color change from pale yellow to colorless was observed. This cycle was repeated once more. The solid was then used as needed for further reactions or measurements. AlS/ZrD(Np) was synthesized in an analogous fashion as AlS/ZrH(Np), using D_2_ in place of H_2_. To synthesize AlS/Zr(alkyl)_2_, pentane (~0.5 mL) was vacuum transferred into a 75 mL pressure reactor containing AlS/ZrH(Np) (200 mg) on a high-pressure/high vacuum line. The reactor was heated at 150 °C for 30 min. In the first 30 s of heating, a color change from colorless to pale yellow was observed. The AlS/ZrNp_2_ + pentane sample has the following bond configuration, as determined by EXAFS: three Zr–O bonds with lengths 1.97(1), 2.16(1), 2.16(1) Å, and two Zr–C bonds with lengths 2.37(1), 2.45(1) Å.

## Supplementary information

Supplementary Information

Description of Additional Supplementary Files

Supplementary Movie 1

## Data Availability

Computational data and code used in this study have been deposited in the iochem-BD database under the following 10.19061/iochem-bd-6-182. All additional information is available in the supplemetary materials.
