# Supplementary material for: Rapid atom-efficient polyolefin plastics hydrogenolysis mediated by a well-defined single-site electrophilic/cationic organo-zirconium catalyst
Source: Nat Commun. 2022 Nov 23;13:7187. doi: 10.1038/s41467-022-34707-6 (PMC9684440; doi:10.1038/s41467-022-34707-6)
Supplement: Supplementary file 1 — Supplementary Information [file 41467_2022_34707_MOESM1_ESM.pdf]

Supporting Information

Rapid Atom-Efficient Polyolefin Plastics Hydrogenolysis Mediated by a Well-Defined Single-Site Electrophilic/Cationic Organo-Zirconium Catalyst

Alexander H. Mason,[1] Alessandro Motta,[2] Anusheela Das,[1] Qing Ma,[1] Michael J. Bedzyk,*[1] Yosi Kratish,*[1] Tobin J. Marks*[1]

Affiliations:

[1] Northwestern University, Evanston IL 60208, United States

[2] Università di Roma "La Sapienza" and INSTM, UdR Roma, Piazzale Aldo Moro 5, I-00185 Roma, Italy

*Corresponding author emails: bedzyk@northwestern.edu, yosi.kratish@northwestern.edu, t-marks@northwestern.edu

Table of Contents

Supplementary Methods S1
General Hexadecane Hydrogenolysis Procedure........ S3
General Polyolefin Hydrogenolysis Procedure........ S4
PE Hydrogenolysis with Air Deliberately Introduced........ S5
Sulfated Alumina (AlS) Synthesis........ S5
Chemisorption of $ZrNp_4$ on AlS........ S5
Synthesis of AlS/ZrH(Np), AlS/ZrD(Np) and AlS/Zr(alkyl)$_2$ (Pentane-treated AlS/ZrH(Np))... S6
Gas-phase NMR Spectroscopic Monitoring of AlS/$ZrNp_2$ Ligand Hydrogenolysis........ S6
Ethane Hydrogenoylsis Control Reaction........ S6
Supplementary Figures S7
Supplementary Tables........ S20
Supplementary Note 1........ S22
DFT Cartesian Coordinates (Å) of All Species Involved in the Depolymerization Process S23
Supplementary References........ S82

Supplementary Methods

All procedures for air- and moisture-sensitive compounds were carried out with rigorous exclusion of O_2 and moisture in flame- or oven-dried Schlenk-type glassware interfaced to a high-vacuum (10^{-5} - 10^{-6} Torr) line or in an argon-filled M-Braun glovebox with a high capacity recirculator (<1 ppm O_2). Argon used on high-vacuum lines (Airgas, UHP-grade) was purified by passage through MnO/vermiculite and activated Davidson 4Å molecular sieve columns. All were solvents dispensed from activated alumina/CuO columns prior to use. *n*-pentane (Sigma) was further purified by drying over Na/K alloy followed by passage through a fiberglass filter in an argon glovebox. Aluminum oxide was purchased from Nanostructured and Amorphous Materials (gamma, nanopowder 20-30 nm). Sulfuric acid (98%) was purchased from Fisher. *n*-hexadecane (**C16**) was purchased from Sigma and was purified by heating at 120 °C over Na for 48h, followed by degassing at room temperature, and was further purified by passage through a 0.22μm PTFE

syringe filter immediately prior to use. All components containing plastic directly contacting **C16** prior to hydrogenolysis experiments (i.e., syringes, syringe filters, needles, Teflon reactor caps) were pumped in the glove box inlet chamber overnight prior to use. Oxygen (UHP grade) used for calcination was purchased from Airgas and used without further purification. Deuterium (Sigma) and hydrogen (Airgas, UHP) were purified by passage through an oxygen/moisture trap (Matheson, model MTRP-0042-XX). Zirconium(IV) chloride and neopentylmagnesium chloride (1.0M solution in Et_2O) were purchased from Sigma and used without further purification. Tetra(neopentyl)zirconium (**$ZrNp_4$**) was synthesized according to literature procedures and purified by sublimation at 70°C and ~10^{-6} Torr. Polymers were dried in the melt (130-165 °C) under high vacuum for 48h before use in the hydrogenolysis reactions.

Physical and Analytical Measurements.
Inductively coupled plasma (ICP) analysis was performed by Galbraith Laboratories Inc., Knoxville, Tennessee. ^{1}H (500 MHz) and ^{13}C (125 MHz) NMR spectra of hydrogenolysis products were obtained with a Bruker Avance III system equipped with a DCH Cryoprobe. ^{1}H MAS (400 MHz) and ^{13}C CP-MAS (100 MHz) Solid State NMR measurements were obtained with a Bruker Avance III system equipped with a 4 mm Bruker HX probe. The rotor was charged with the sample in an Ar glove box. The rotor speed was set to 14kHz for all spectra. Gas chromatography/mass spectrometry (GC/MS) analysis of hydrogenolysis product mixtures was carried out on an Agilent GCMSD equipped with a DB5 column (oven program: **1.** 2 min. 50°C hold **2.** 30°C / min ramp **3.** 2 min hold at 300°C). Split mode injection at 2µL / injection and a 100:1 split ratio was used. For GC-MS quantification of *n*-hexadecane, a four-point calibration (0.1, 0.2, 0.3, 0.4 mg/mL) was carried out for each group of samples analyzed by GC/MS with a target sample concentration of ~0.2 mg/mL. Calibration standards were prepared in 1,1,2,2-tetrachloroethane (TCE), and stored in airtight Teflon-valved glassware. Diffuse reflectance infrared spectroscopy (DRIFTS) measurements were taken under Ar on Thermo 6700 infrared spectrometer equipped with a Harick Praying Mantis DRIFTS attachment. ZnS windows were used for the DRIFTS cell. Anhydrous KBr with Ar glovebox atmosphere in the cell was used as a background. BET surface area measurements were carried out with a Micromeritics 3Flex Surface Characterization Analyzer.

X-ray absorption spectroscopy.
X-ray absorption near edge structure (XANES) and extended X-ray absorption fine structure (EXAFS) measurements at Zr K-edge (17998 eV) were performed at the 5 BM-D beamline of the DND-CAT at the Advanced Photon Source. A double Si (111) monochromator was used for energy selection with an energy resolution of $\Delta E/E = 1.4\times10^{-4}$. The X-ray energy was calibrated using a metallic Zr foil. The incident X-ray intensity was measured by a spectroscopy-grade ionization chamber (FMB-Oxford) filled with 600 He/100 N_2 (Torr) and was detuned to 60 % of its maximum for harmonic rejection. EXAFS spectra were collected in fluorescence mode using a passivated implanted planar silicon (PIPS) detector (Canberra). The sample and the detector were positioned 45 deg and 90 deg, respectively, to the X-ray beam direction. Energy scans were executed from 250 eV below to 550 eV above the Zr K edge which produces the EXAFS spectra.

Catalyst samples of **AlS/$ZrNp_2$** with a Zr loading of 1.40 wt% were pressed into a sample holder for the EXAFS measurements. The samples were sealed airtight in a THMS600 Linkam cell inside the glove box, which was over pressured with ultrapure Ar gas. The positive Ar pressure was maintained throughout the measurement. After measuring the **AlS/$ZrNp_2$** sample, *in-situ* catalyst hydrogenolysis was carried out by flowing H_2 gas with a flow rate of 50 sccm at room temperature

with EXAFS data collected every 15 min for 2 h until no further changes were observed. This was followed by heating the sample to 150 °C with continued H_2 flow. Data were collected every 15 min for up to 2 h. No changes were observed after this step of the treatment. There was a change in the structure during the hydrogenolysis reaction at room temperature and these data are shown in manuscript (**Fig. 5E**). XANES data extraction, normalization, and background subtraction were performed using Demeter:Athena. EXAFS data analysis was carried out using the software Demeter:Artemis. The bond lengths (R) and coordination number (N) were obtained by a least-square fit in the R-space of the nearest neighbors using k_2-weighted Fourier transform fitting parameter. For data analyses, a standard reference model compound was used: powder 2,6-$^{t}Bu_2PhOZrBn_3$, which was measured in fluorescence mode.

Gel permeation chromatography.

GPC analysis was performed using a Polymer Laboratories PL-GPC 220 equipped with three PLgel 10um MIXED-B LS 300 x 7.5 mm columns and using 1,2,4-trichlorobenzene stabilized with 0.0125 wt% BHT at 150 °C. Calibration was performed using polystyrene standards (860-3,752,000 g/mol). Samples were prepared by dissolving the polymer in stabilized TCB at 150 °C with gentle shaking overnight to yield a solution concentration of ~1.0 mg/mL, and the sample was filtered through a 0.5 μm porous stainless-steel filter prior to measurement.

Computational details.

DFT-based simulations were performed with the /Quickstep package, using a hybrid Gaussian and plane wave method.[1] A double quality DZVP Gaussian basis set was employed for the Al and Zr atoms, and a triple quality TZVP Gaussian basis set was employed for all the other atoms.[2] The Goedecker-Teter-Hutter pseudopotentials[3] together with a 400 Ry plane wave cutoff were used to expand the densities obtained with the Perdew-Burke-Ernzerhof (PBE)[4] exchange-correlation density functional, and vdW forces are taken in account with the Grimme D3 Method.[5] Only the gamma point was considered in a supercell approach. Periodic boundary conditions are applied in all directions of space. Molecular graphics were produced by the CHEMCRAFT graphical package.[6] *Gibbs free energy profile*: enthalpic and entropic contributions along the reaction pathway were evaluated by performing the frequency calculation of the molecular species at 298.15 K and 1 atm as implemented in the G16 code.[7] In this context, adsorbed catalysts were modeled by simple molecular species, and only the entropic contribution related to vibrational motion is considered. G16 calculations were performed at the level of the B3LYP hybrid functional. The standard all-electron 6-311G** basis set was used for all atoms. The enthalpic and entropic contributions were then "appended" to the SCF energy profile to obtain the Gibbs free energy profile.

General Hexadecane Hydrogenolysis Procedure.

In the glovebox, **C16** (typically 0.50 g) was passed through a 0.22 μm PTFE syringe filter directly into a dry heavy-walled glass pressure reactor (350 mL volume) containing a 10mm ovoid stir bar. The desired amount of **AlS/ZrNp$_2$** (typically 25 mg, 0.02 mol% Zr) was added to the reactor, and was sealed with a threaded Teflon cap with an NPT valve installed. The vessel was carefully removed from the glovebox and interfaced with a high-pressure/high-vacuum line. The reactor was degassed at room temperature (30 s), then charged to the desired pressure of H_2. The reactor was placed in an oil bath set to the desired reaction temperature. Once the oil bath thermocouple reached reaction temperature, the time interval was started. At the end of the time interval, the

reactor was removed from the oil bath, then cooled via a water bath to room temperature. Headspace samples were taken, if needed, at this point. Note, for determining reaction conversion as a function of time, the reaction temperature was decreased to 90 °C to keep reaction conversions below 15% and preserve the accuracy of the apparent reaction rate. The reactor was vented through the NPT valve and opened to air. Approx. 5 mL dichloromethane (DCM) or TCE was used to wash the Teflon cap and the interior of the NPT valve. These washings were added to the reactor. The washings were transferred to a syringe equipped with a 0.22 µm PTFE filter. The reactor was washed 4x with ~10 mL portions of clean solvent, with each washing being added to the syringe. The washings were passed through the filter directly into a 100 mL volumetric flask. The filter was washed 4x with ~5 mL portions of clean solvent with washings being added to the volumetric flask. Pipettes used to transfer solvent solutions were also washed with clean solvent and added to the volumetric flask. The solution was diluted to the calibration mark, then diluted further using standard analytical techniques to ~0.2 mg/mL. A four-point external calibration (~ 0.1, 0.2, 0.3, and 0.4 mg/mL) of hexadecane in TCE was used to determine the concentration of the sample *via* GC/MS. The series of external calibration standards were run for each round of reaction sample measurements. Uncertainty in kinetic measurements was taken to be ±1% (absolute) to account for analytical uncertainty, except in the case of varied H_2 pressure experiments, where uncertainty was taken to be 3 standard deviations of the data set.

General Polyolefin Hydrogenolysis Procedure.

In the glovebox, **AlS/ZrNp$_2$** and the desired amount of polymer were loaded into a dry heavy-walled glass pressure reactor (350 mL volume) containing a 10mm ovoid stir bar, typically with ~15% mass loading of catalyst (0.02-0.04 mol% Zr). The easily implemented and versatile hydrogenolysis reactor and analytical procedures serve four functions: i) For constant polymer and catalyst, to screen products as a function of reaction conditions such as temperature, H_2 pressure, reaction time, and stirring rate. ii) For constant polymer and reaction conditions, to screen different catalysts in terms of activity and product mix selectivity. iii) For constant catalyst and reaction conditions, to screen different polymers in terms of activity and product mix selectivity. iv) The optically transparent heavy-wall glass reactor can be filled in the glove box, is high-vacuum line compatible, and allows ready visualization of the reaction progress (solid→liquid→gas) as well as stirrer malfunction or possible coke formation (never observed). The polymers were prepared by shaving from a larger puck of pre-melted stock. The reactor was sealed with a threaded Teflon cap having an installed NPT valve. The vessel was carefully removed from the glovebox and interfaced to a high-pressure/high-vacuum line. The reactor was degassed at room temperature (30 s), then charged to 2 atm H_2. The reactor was then placed in an oil bath set to the desired temperature (polyethylene 150°C, polypropylene, polyethylene-*co*-1-octene, HDPE food container cap 190 or 200°C). Once the polymer melted and contacted the catalyst in the melt, the time interval was started. Stirring was typically set to 300 rpm initially, then increased to ~800 rpm after a sufficient decrease in polymer viscosity. At the end of the time interval, the reactor was removed from the oil bath, then air-cooled to room temperature. Headspace samples were taken at this point. Headspace samples were taken by expansion of the reactor contents into an evacuated 500 mL Teflon-valved glass bulb. Individual samples for analysis were taken via septum and gastight headspace syringe (1 mL). The reactor was vented through the NPT valve and opened to air. Approx. 5 mL DCM was used to wash the Teflon cap and the interior of the NPT valve. These washings were added to the reactor. Solids were suspended in the DCM washings and then filtered to isolate the solids. The reactor was washed enough times to remove all residue. The solids were

washed ~3x with 5 mL portions of DCM, then dried at ~1 Torr overnight. This product was assigned the "solids fraction". GPC analysis was carried out for this product fraction. The DCM washings were collected and the DCM was removed under reduced pressure overnight (~1 Torr), these DCM-soluble hydrocarbons are assigned as "DCM extract". Note that some volatile liquid hydrocarbons (C5-C7) are lost during DCM removal, however, we find that <5 wt% of the total product mass is lost. Approximately 10 mg of the DCM extract fraction was added to ~1 mL DCM for GC/MS analysis. Mass not accounted for is assigned to the "volatiles" fraction, and the presence of light hydrocarbons is confirmed *via* GC-FID. The percent conversion of a polyolefin hydrogenolysis reaction is defined as the mass of "volatiles" and "DCM extract" produced as a percentage of the initial polyolefin mass. Uncertainty in polymer hydrogenolysis products mass was estimated to be 20 mg (1-2% of initial polymer mass) and was carried over into calculation of polymer hydrogenolysis activities.

PE Hydrogenolysis with Air Deliberately Introduced.

For the control, 1g of commercially available polyethylene (Dow Engage 8402) and 0.3g **AlS/ZrNp$_2$** (0.07 mol% Zr) were combined in a glass reactor. The reaction was carried out as described in "General Polyolefin Hydrogenolysis Procedure" (200°C, 2 atm H_2, 25 min, Activity: 3350 mol CH_2 units•(mol Zr)$^{-1}$•(h)$^{-1}$). The air-exposed reaction was charged with catalyst, PE, and H_2. After charging the vessel with H_2, the reactor was connected to an evacuated manifold (<10 mL total volume). The reactor was opened to the manifold and 5 mL of ambient air (~1 mL O_2, ~1:1 molar ratio of O_2 : Zr) was injected *via* septum and gastight syringe. The reactor remained open to the manifold for 5 minutes to allow for diffusion of the injected air and for any potential reaction to occur. Note, the O_2 : H_2 ratio is well below the explosive limits of H_2 in pure oxygen.[8] The reaction was then carried out in an analogous fashion (200°C, 2 atm H_2, 25 min) to the control (Activity: 3432 mol CH_2 units•(mol Zr)$^{-1}$•(h)$^{-1}$). Remarkably, there was no measurable effect on reaction rate after injecting of 5 mL of ambient air into the reactor.

Sulfated Alumina (AlS) Synthesis.

Under atmospheric conditions, sulfuric acid (2.0 M aqueous solution prepared from deionized (DI) water, 288 mL) was added to 7.0 g of aluminum oxide (gamma, nanopowder 20-30 nm) with stirring. The suspension was stirred for 30 min, then centrifuged (4000 rpm, 5 min). The supernatant was then discarded, and the alumina was re-suspended in DI water and centrifuged; this step was repeated until a pH of ~6 was achieved (typically 7 washings total). The resulting solids were then dried at 120°C and ~10^{-6} Torr for 18h. The solids were then crushed *via* mortar and pestle and sieved to 180 mesh (80 μm). The powder was loaded into a quartz boat in a reaction tube, and the tube was placed in a tube furnace for calcining at 550°C under flowing O_2 (~2L / min) for 3h. The tube was then interfaced with the high vacuum line and pumped down to ~10^{-6} Torr for 1h at 450°C. The furnace was finally cooled under vacuum and brought into an argon glovebox. The **AlS** (white powder, 4.83g) was collected and stored under argon.

Chemisorption of ZrNp$_4$ on AlS.

In a dry two-sided fritted dried reaction vessel, 25 mL of pentane was condensed onto well-mixed quantities of **ZrNp$_4$** (66.67 mg, 0.177 mmol) and **AlS** (1.000 g). The resulting slurry was stirred at 25 °C for 1 h, and then filtered. After chemisorption, the solid attains a pale-yellow color. The impregnated support was collected on the frit and washed five times with ~10 mL portions of

pentane and then dried in vacuo for 1 h. ^{1}H NMR was used to check for the presence of remaining physisorbed **$ZrNp_4$** by addition of ~10 mg of solids directly to an NMR tube with benzene-d_6 or toluene-d_8 used as the solvent. If residual organometallic was present, the catalyst was additionally washed with pentane as described above until **$ZrNp_4$** was not visible in the ^{1}H NMR. The catalyst was stored at -40°C in an argon glovebox until needed. The catalyst (**AlS/$ZrNp_2$**) loading was 1.40 wt. % Zr (average of two batches at 1.38 and 1.42 wt. % Zr) by ICP-AES. The catalyst BET surface area of 184 m^2/g was determined by N_2 physisorption (0.50 Zr/nm^2).

Synthesis of AlS/ZrH(Np), AlS/ZrD(Np) and AlS/Zr(alkyl)$_2$ (Pentane-treated AlS/ZrH(Np)).

In the glovebox, **AlS/$ZrNp_2$** (200 mg) was added to a dry 75 mL heavy-walled glass pressure reactor. The reactor was sealed, interfaced to a high pressure / high vacuum line, evacuated, then charged with 1 atm H_2. The reactor was heated to 150 °C for 5 min, then evacuated. A color change from pale yellow to colorless was observed. This cycle was repeated once more. The solid was then used as needed for further reactions or measurements. **AlS/ZrD(Np)** was synthesized in an analogous fashion as **AlS/ZrH(Np)**, using D_2 in place of H_2. To synthesize **AlS/Zr(alkyl)$_2$**, pentane (~0.5 mL) was vacuum transferred into a 75 mL pressure reactor containing **AlS/ZrH(Np)** (200 mg) on a high pressure / high vacuum line. The reactor was heated at 150°C for 30 min. In the first 30s of heating a color change from colorless to pale yellow was observed. The **AlS/$ZrNp_2$** + pentane sample has the following bond configuration, as determined by EXAFS: three Zr-O bonds with lengths 1.97(1) Å, 2.16(1) Å, 2.16(1) Å and two Zr-C bonds with lengths 2.37(1) Å, 2.45(1) Å.

Gas-phase NMR Spectroscopic Monitoring of AlS/$ZrNp_2$ Ligand Hydrogenolysis

AlS/$ZrNp_2$ (41.50 mg) was added to a J-young NMR tube. The tube was interfaced with a high vacuum line, evacuated, and charged with 1 atm H_2. 5 minutes later, a ^{1}H NMR spectrum was taken of the gas phase, and HNp was found to be the major product. The tube was removed and shaken, and the following spectrum was collected at 14 min. This process was repeated for 20-90 minute time points with 10-minute intervals. Interestingly, the HNp is converted at r.t. to mainly ethane and methane. The tube was then heated to 150°C for 30 min to facilitate full hydrogenolysis of alkanes present to ethane and methane as found ^{1}H NMR and headspace GC-FID.

Ethane Hydrogenoylsis Control Reaction

A heavy-walled glass pressure reactor containing **AlS/ZrH(Np) (**0.20 g) was interfaced with a high vacuum line and held under a high vacuum for 30 min. The vessel was charged with a 1:1 molar ratio of ethane:H_2 at 1 atm. The reactor was heated to 120°C for 1h with no visual change occurring in the catalyst. The reactor was then cooled down, and 1mL samples of headspace were analyzed for methane content. Note that a trace amount of methane is observed due to some neopentyl ligand hydrogenolysis from the **AlS/ZrH(Np)** catalyst. This is supported by a control experiment with **AlS/ZrH(Np)** and only H_2 in which similar amounts of methane (and trace ethane) are observed.

Supplementary Figures

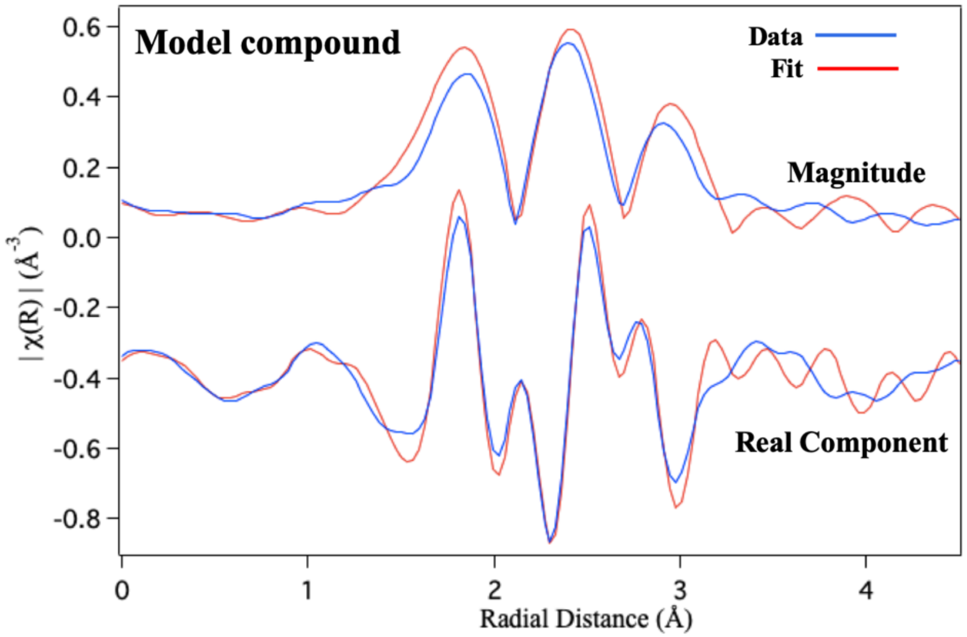

Fig. S1. Zr EXAFS spectrum of model compound 2,6-^{t}Bu$_2$PhOZr(benzyl)$_3$. Real component of spectrum offset by -0.3 Å^{-3}.

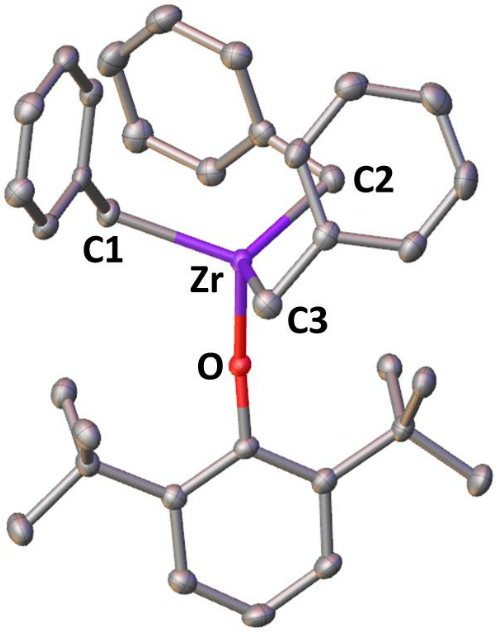

Fig. S2. X-ray Crystallographic Molecular Structure of 2,6-^{t}Bu$_2$PhOZr(benzyl)$_3$

Olex2 drawing of X-ray crystallographic molecular structure of 2,6-$^{t}Bu_2$PhO-Zr(benzyl)$_3$ (CCDC deposition number 2125175). Hydrogen atoms are omitted for clarity. Key bond lengths: Zr-O 1.947(1) Å, Zr-C1 2.269(2), Zr-C2 2.278(2), Zr-C3 2.290(2).

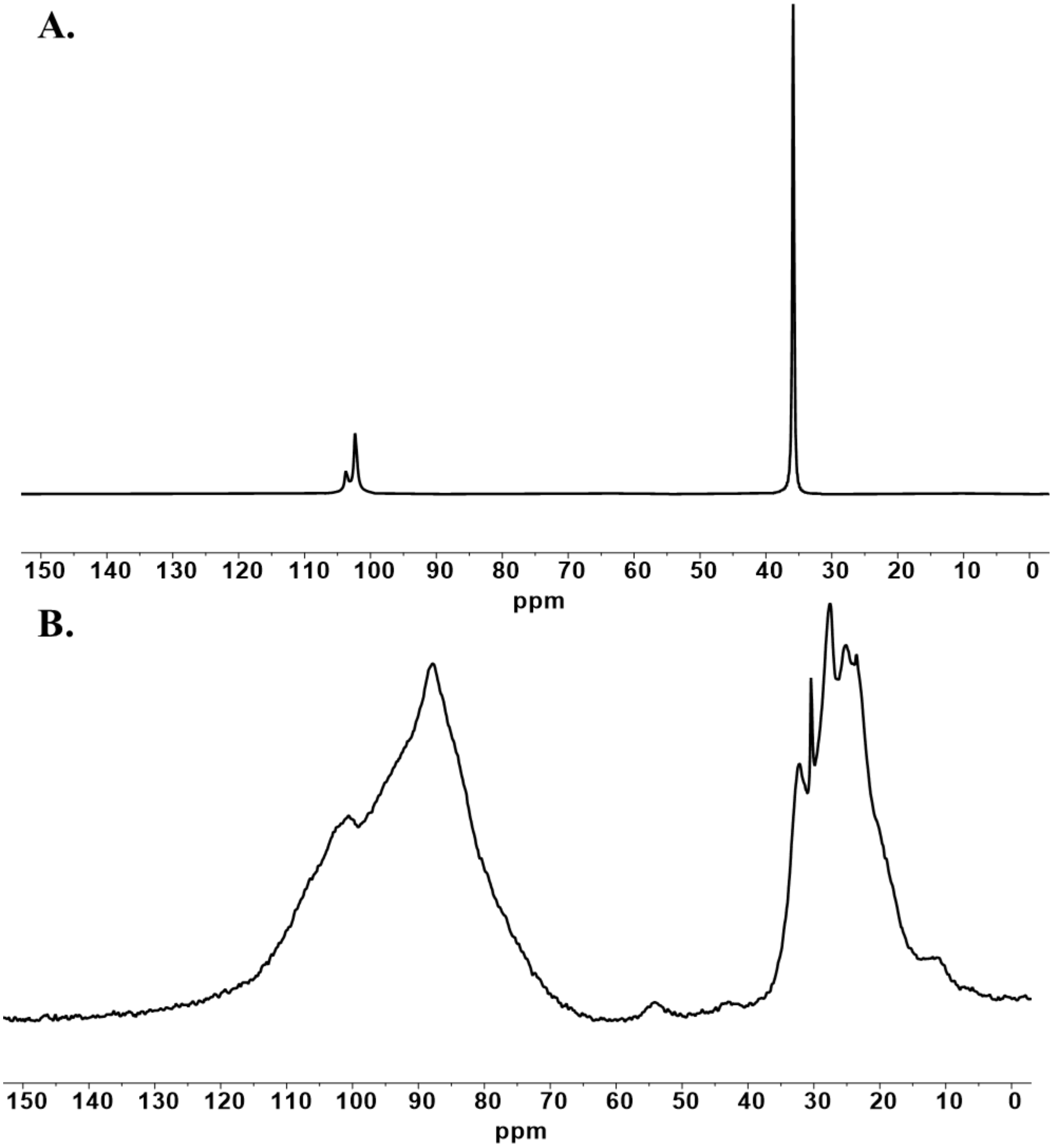

Fig. S3. ^{13}C CP-MAS NMR Spectra of ZrNp$_4$ and AlS/ZrNp$_2$ ^{13}C CP-MAS NMR. 100 MHz, 14 kHz rotor speed, 2ms contact time, 5s recycle delay. (A) ZrNp4. (B) AlS/ZrNp2, 99% ^{13}C-enriched at the methylene (CH_2) carbon.

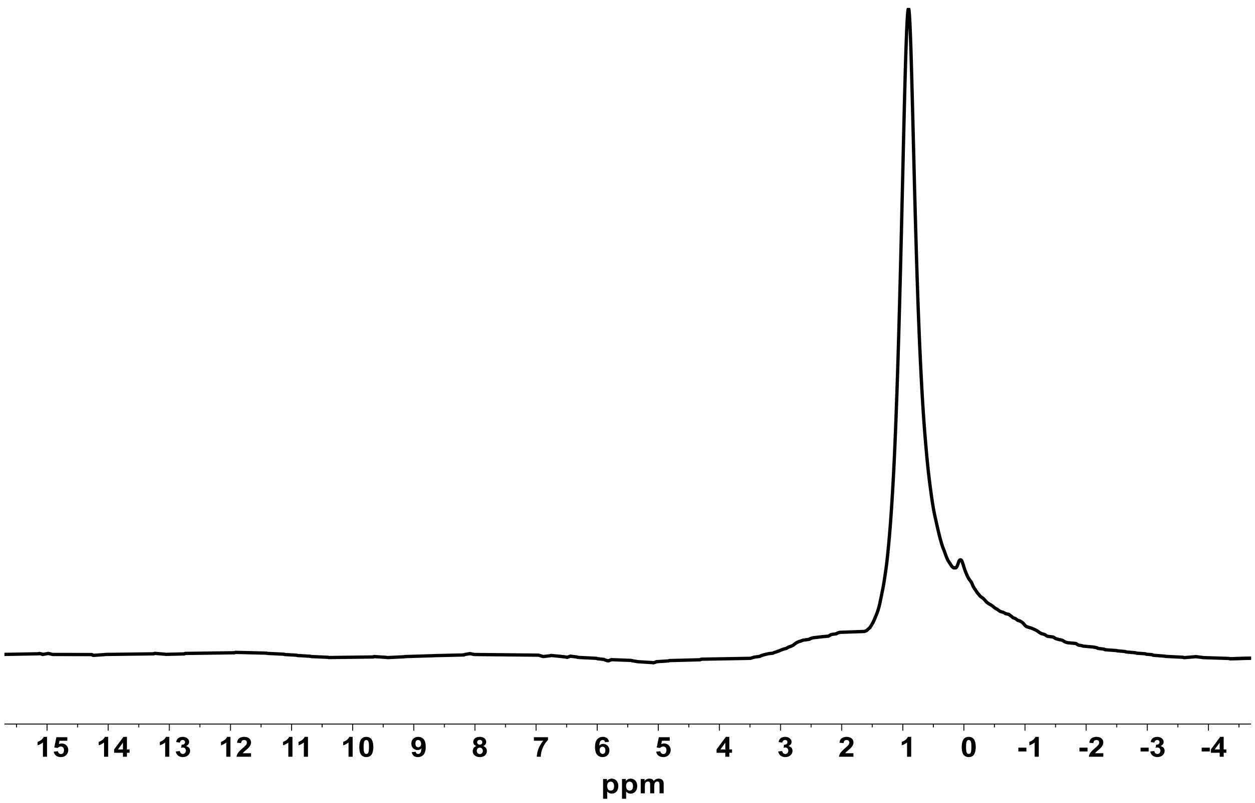

Fig. S4. ^{1}H MAS NMR Spectrum of AlS/ZrD(Np) 400 MHz, 14 kHz rotor speed.

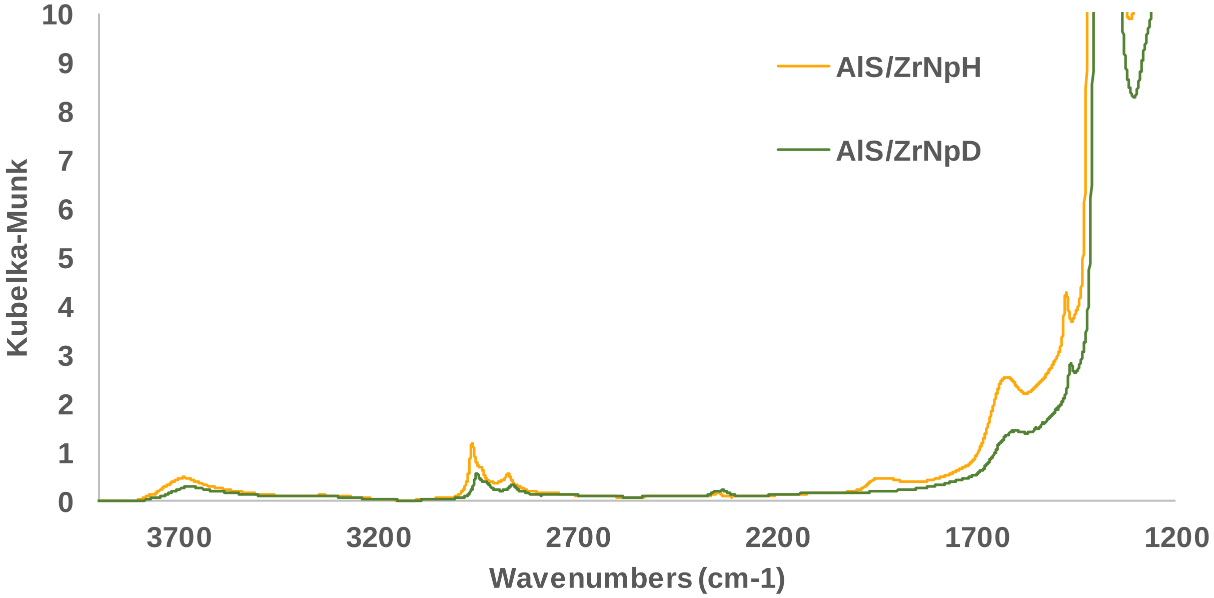

Fig. S5. DRIFT spectra of AlS/ZrH(Np) and the deuterated analog AlS/ZrD(Np).

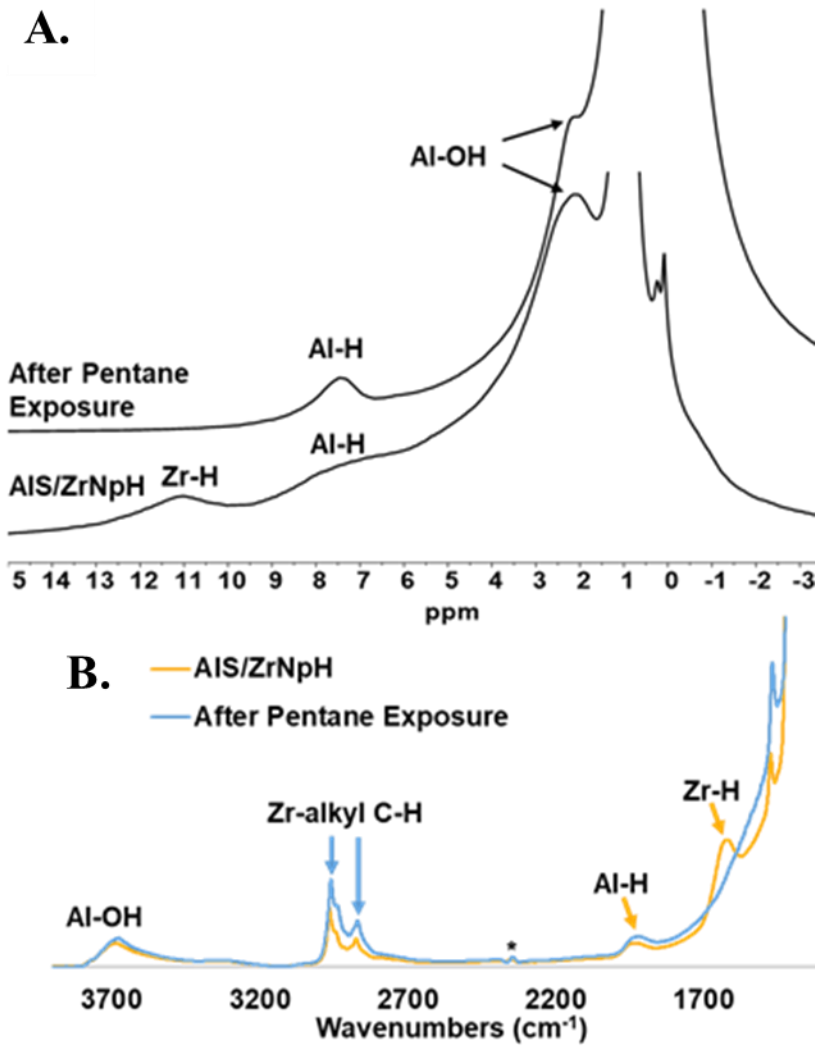

Fig. S6. ^{1}H MAS-NMR and DRIFTS Spectra of Pentane-Exposed AlS/ZrH(Np).
A. ^{1}H MAS-NMR spectrum of AlS/ZrH(Np) pentane exposure (30 min, 150°C) B. DRIFTS spectra of AlS/ZrH(Np) before and after pentane vapor exposure. *Atmospheric CO_2 background artifact.

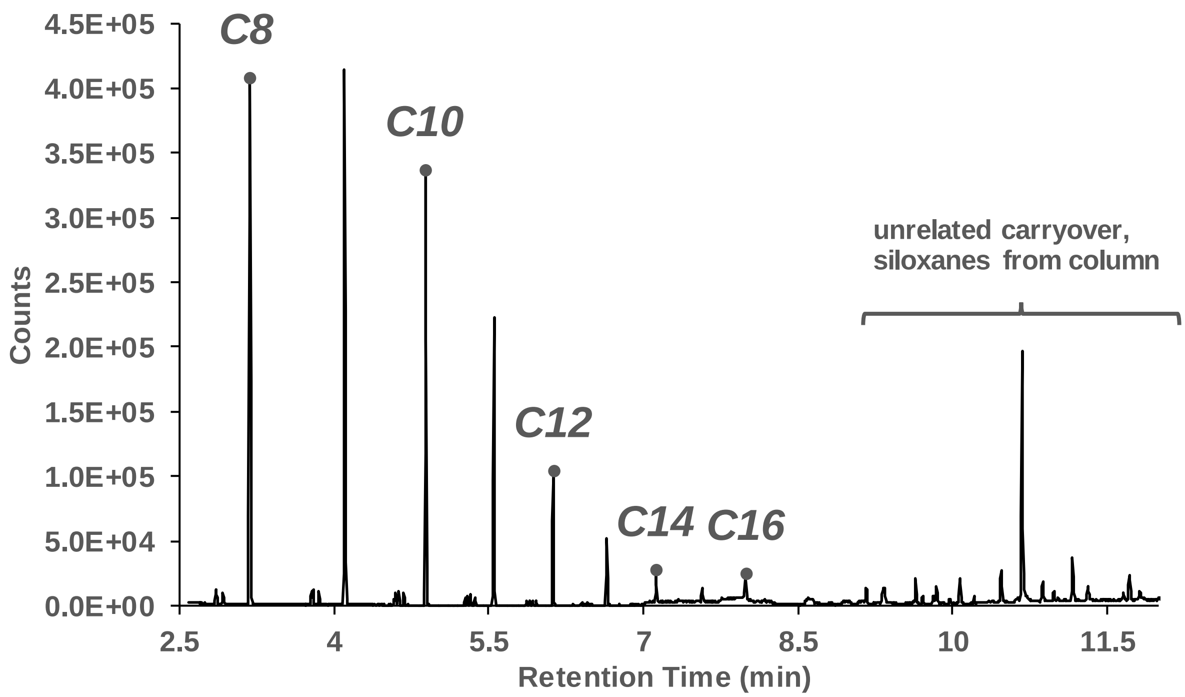

Fig. S7. GC/MS Chromatogram of Liquid C16 Hydrogenolysis Reaction Products. GC/MS chromatogram of liquid C16 hydrogenolysis reaction products (Table 1, entry 8, Video S1). Residual C16 is present from imperfect contact between substrate and catalyst.

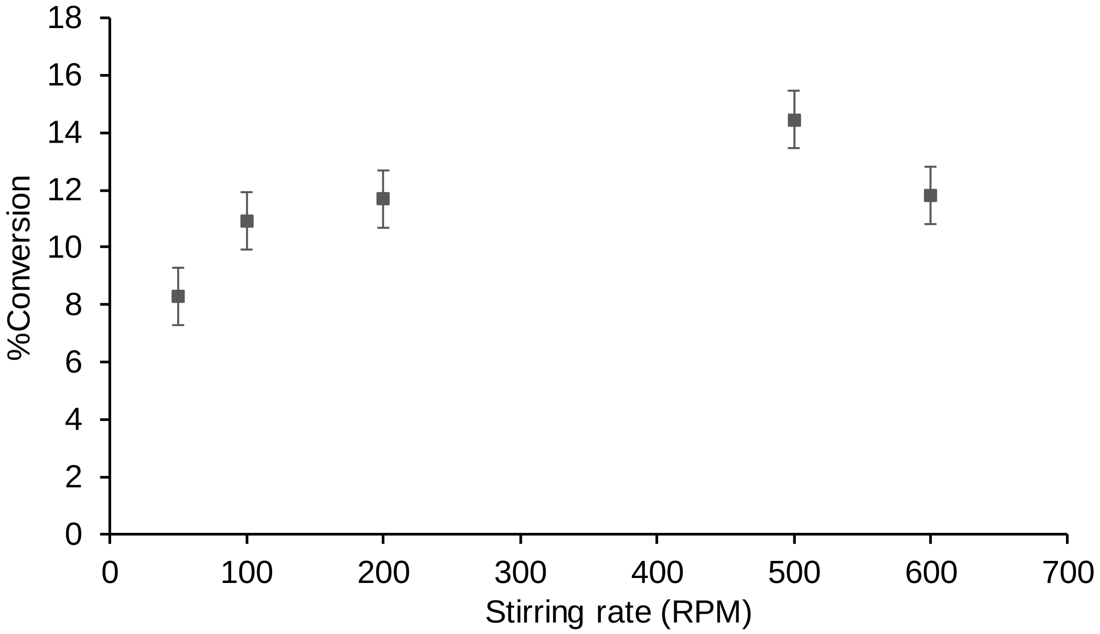

Fig S8. C16 Consumption During Hydrogenolysis as a Function of Stirring Rate. Plot of C16 hydrogenolytic conversion as a function of reactor stirring rate, 50-600 RPM. (0.02 mol% Zr, 2 atm H_2, 15 min, 120°C, varied stir rate). Note: stirring significantly above 750 RPM causes C16 and catalyst to splatter on the reactor walls, negatively impacting reaction rates. Error bars represent ±1 % conversion in C16 (absolute) uncertainty to account for analytical uncertainty and variations between GC-MS injections.

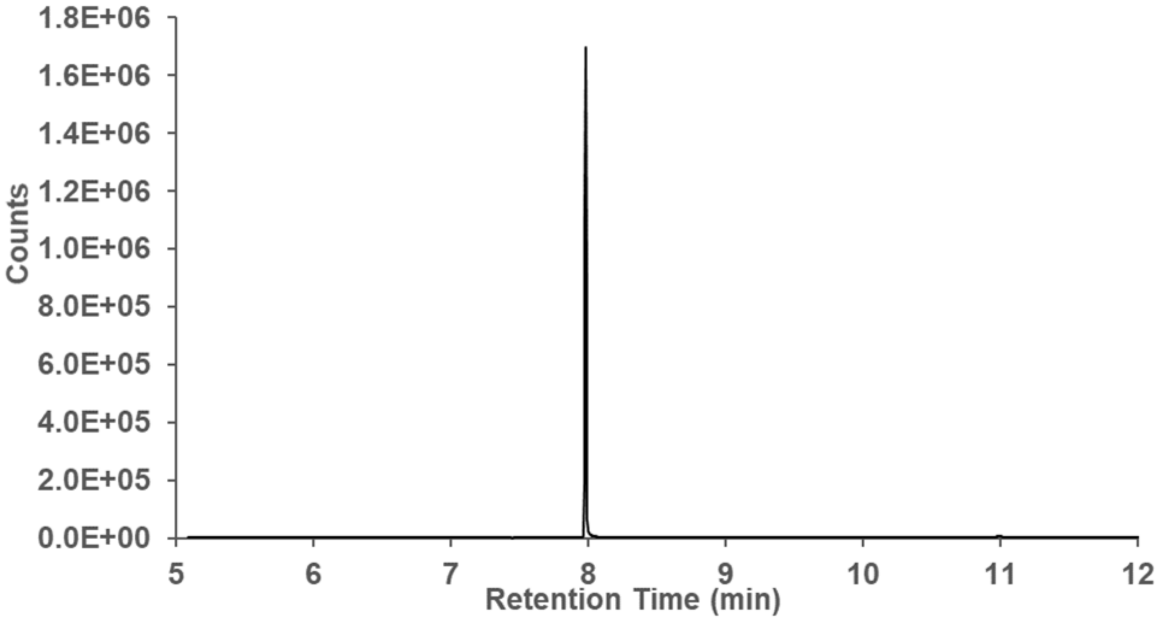

Fig. S9. GC/MS chromatogram of hexadecane from a hydrogenolysis control experiment with only C16 and AlS C16 retention time 8.0 min. (0.097g AlS, 0.500g C16, 2 atm H_2, 150°C, 24h), (Table 1, entry 10).

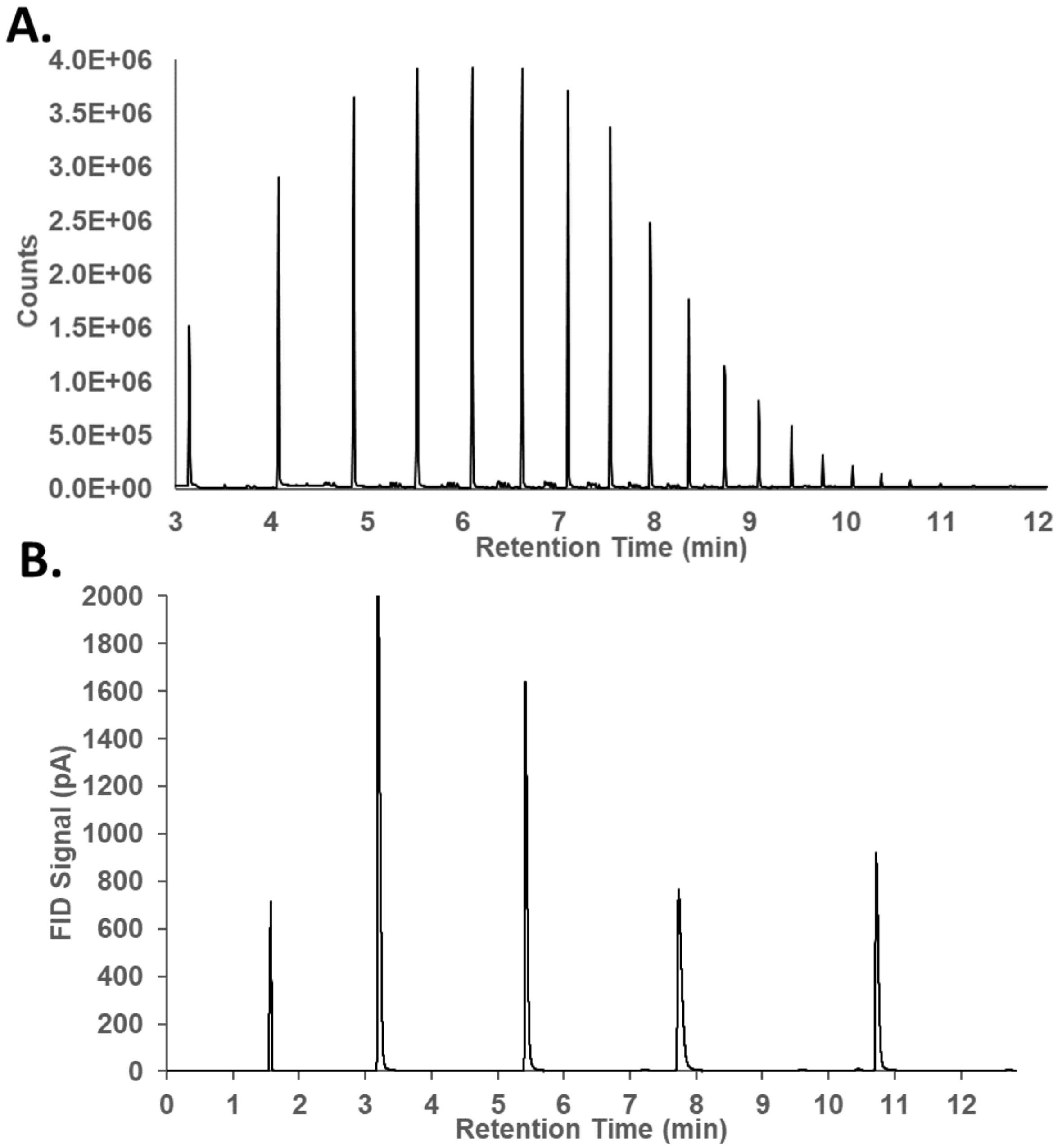

Fig. S10. PE Hydrogenolysis Reaction Products. DCM extract from PE hydrogenolysis (0.03 mol% Zr, 2 atm H_2, 200 °C, 48 min). (Table 1, Entry 1). A. GC/MS chromatogram of DCM extract. B. Headspace GC/FID chromatogram of the volatile fraction.

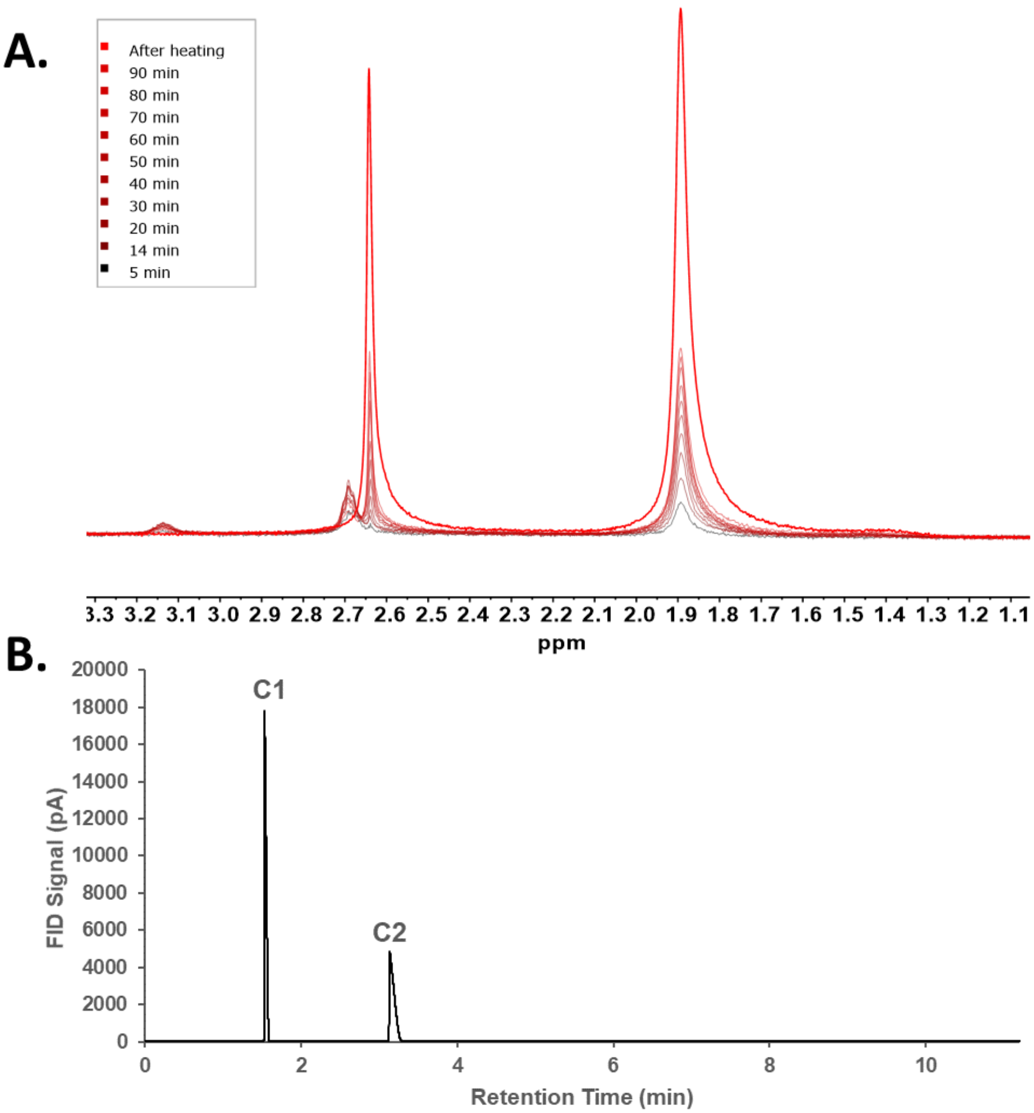

Fig. S11. Gas-phase NMR Spectroscopic Monitoring of AlS/ZrNp$_2$ Hydrogenolysis
A. Stacked ^{1}H NMR spectra of neopentane hydrogenolysis at room temperature (~20 mol% Zr with respect to CH_2 units) and after heating to 150°C. (600 MHz, propane (CH_2) δ3.15 ppm; neopentane + CH_3 resonances from propane and isobutane δ2.70 ppm; ethane δ2.64 ppm; methane δ1.89 ppm). B. GC-FID Chromatogram of NMR tube headspace after heating to 150 °C for 30 min.

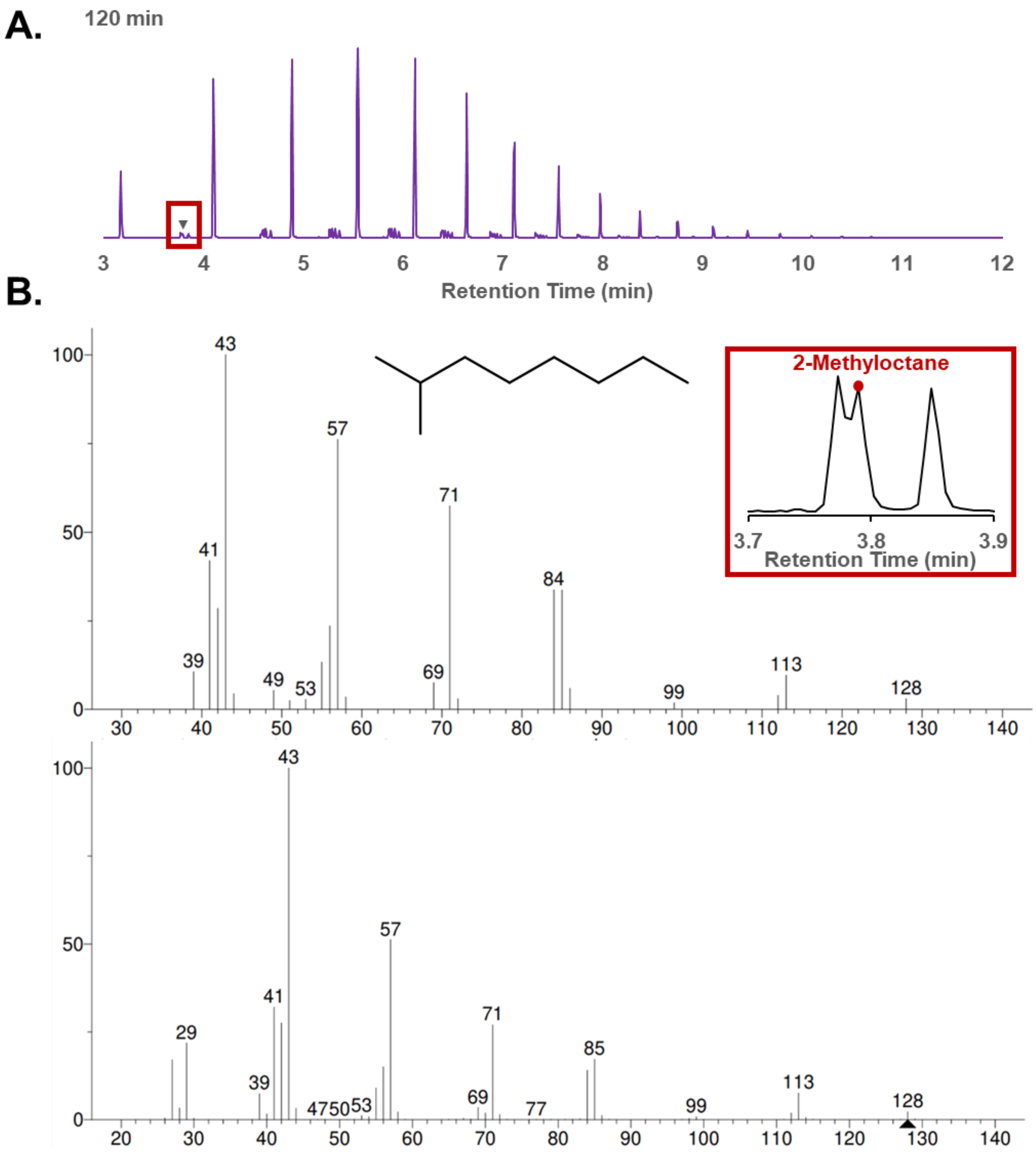

Fig. S12. GC/MS Analysis of the Minor Alkane Branching Products in Catalytic PE Hydrogenolysis. A. GC/MS chromatogram of DCM extract from **AlS/ZrNp$_2$**-catalyzed **PE** hydrogenolysis after 120 min (150° C, 2.0 atm H_2, 0.03 mol% Zr). Trace chain branching denoted with "▼". B. MS of 2-methylocatne produced by PE hydrogenolysis (top) and library-indexed MS of 2-methyloctane (bottom). Insert: Close-up of GC/MS chromatogram showing elution of 2-methyloctane.

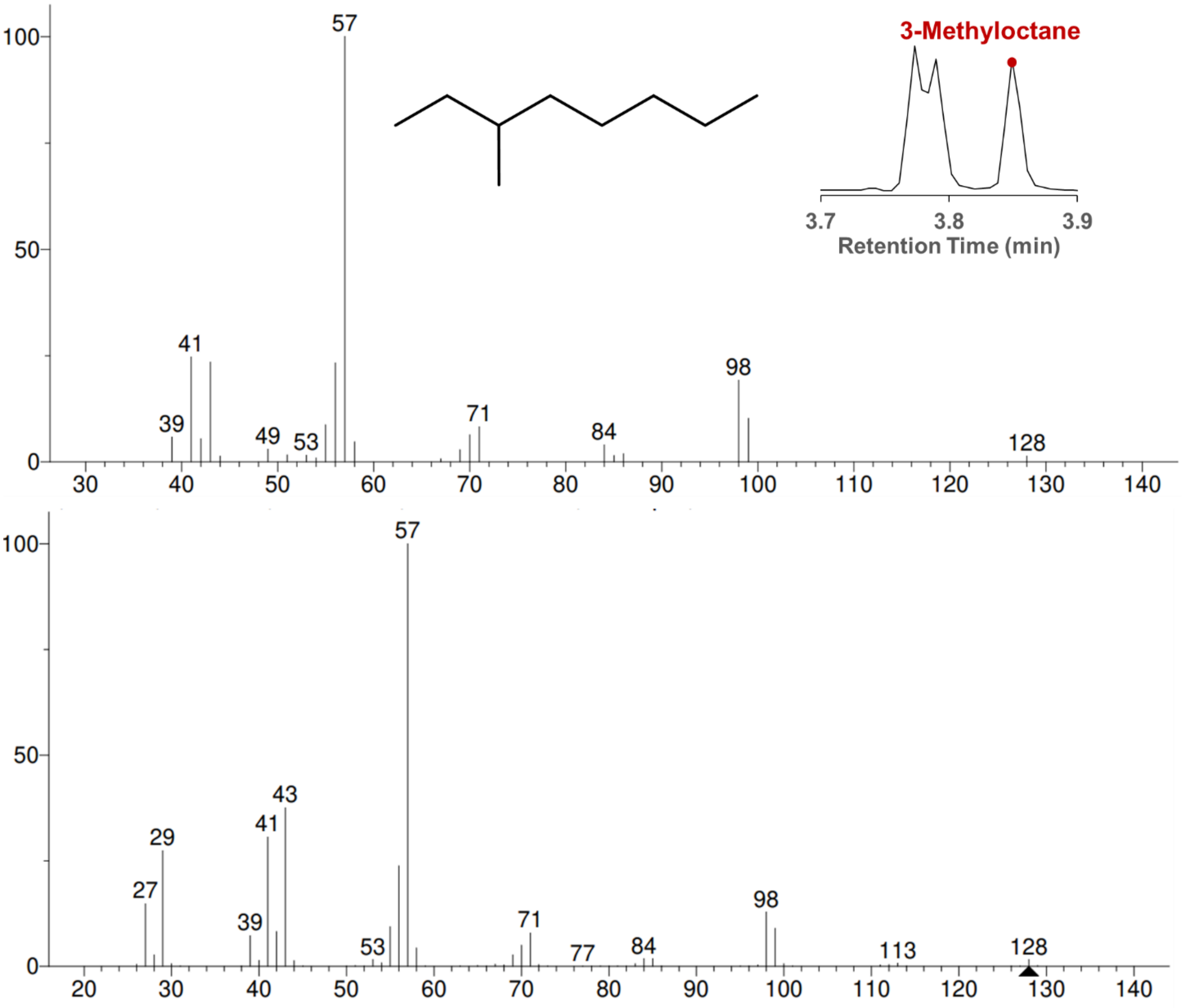

Fig. S13. GC/MS Analysis of the Minor Alkane Branching Products in Catalytic PE Hydrogenolysis. MS of 3-methylocatane produced by PE hydrogenolysis (0.03 mol% Zr, 150°C, 120 min, 2 atm H_2) (top) and library-indexed MS of 3-methyloctane (bottom). Insert: Detail of GC/MS chromatogram showing elution of 3-methyloctane.

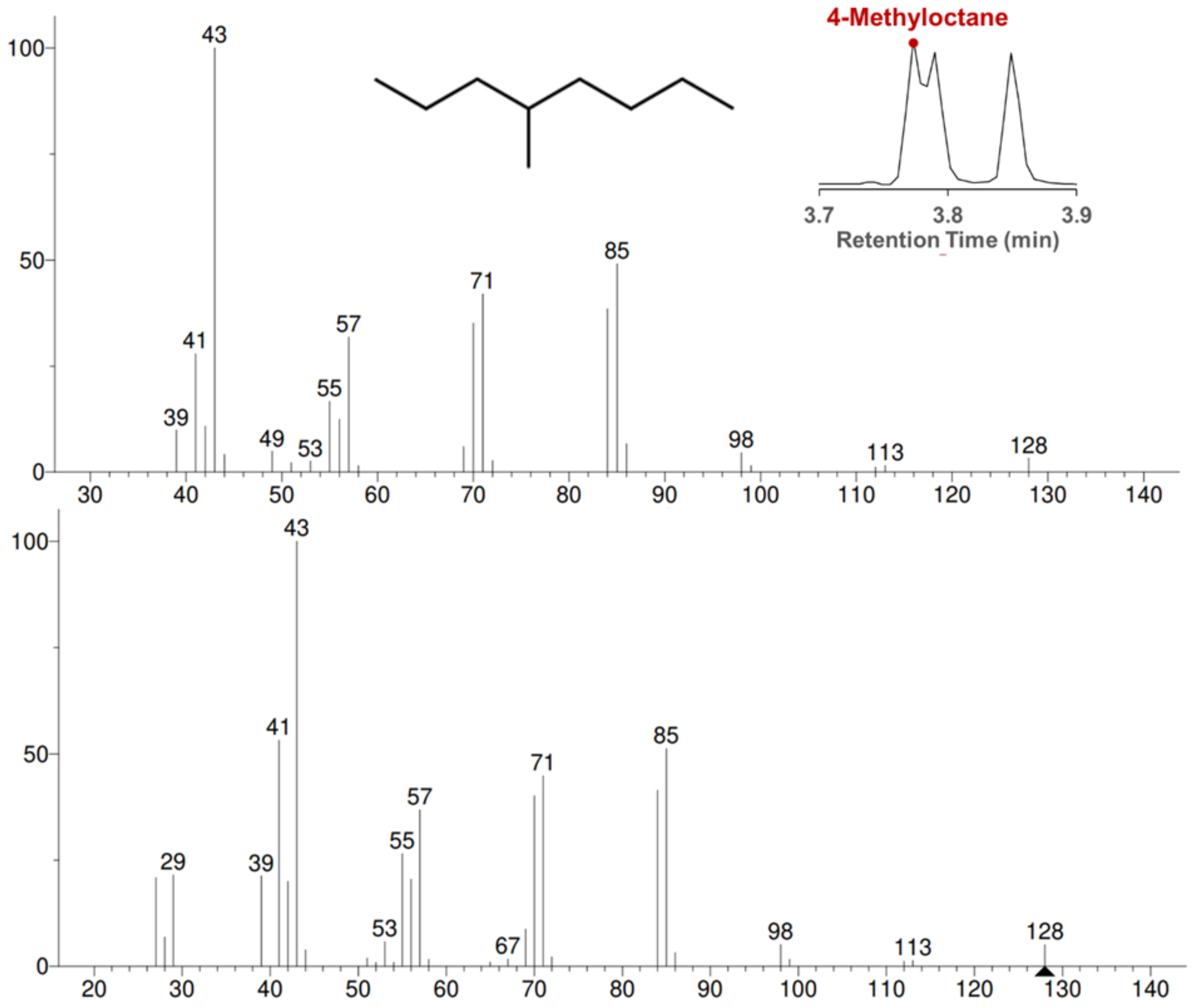

Fig. S14. GC/MS Analysis of the Minor Alkane Branching Products in Catalytic PE Hydrogenolysis. MS of 4-methylocatne produced by PE hydrogenolysis (0.03 mol% Zr, 150°C, 2h 2 atm H_2) (top) and library-indexed MS of 4-methyloctane (bottom). Insert: Detail of GC/MS chromatogram showing elution of 4-methyloctane.

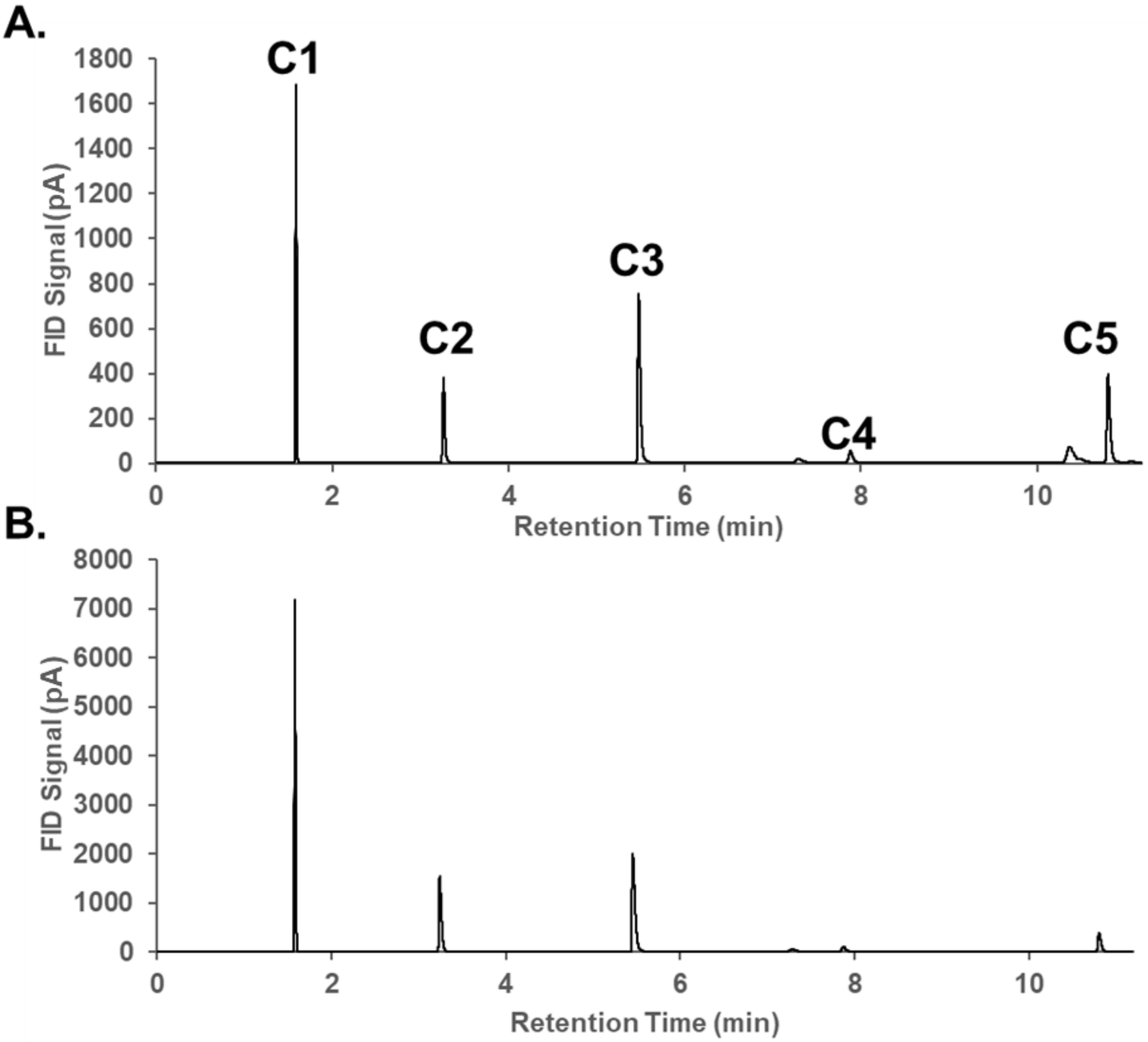

Fig. S15. GC/FID chromatograms of the volatile fraction from i-PP hydrogenolysis experiments. A. 60-minute reaction time (Table 1, entry 4) B. 120-minute reaction time.

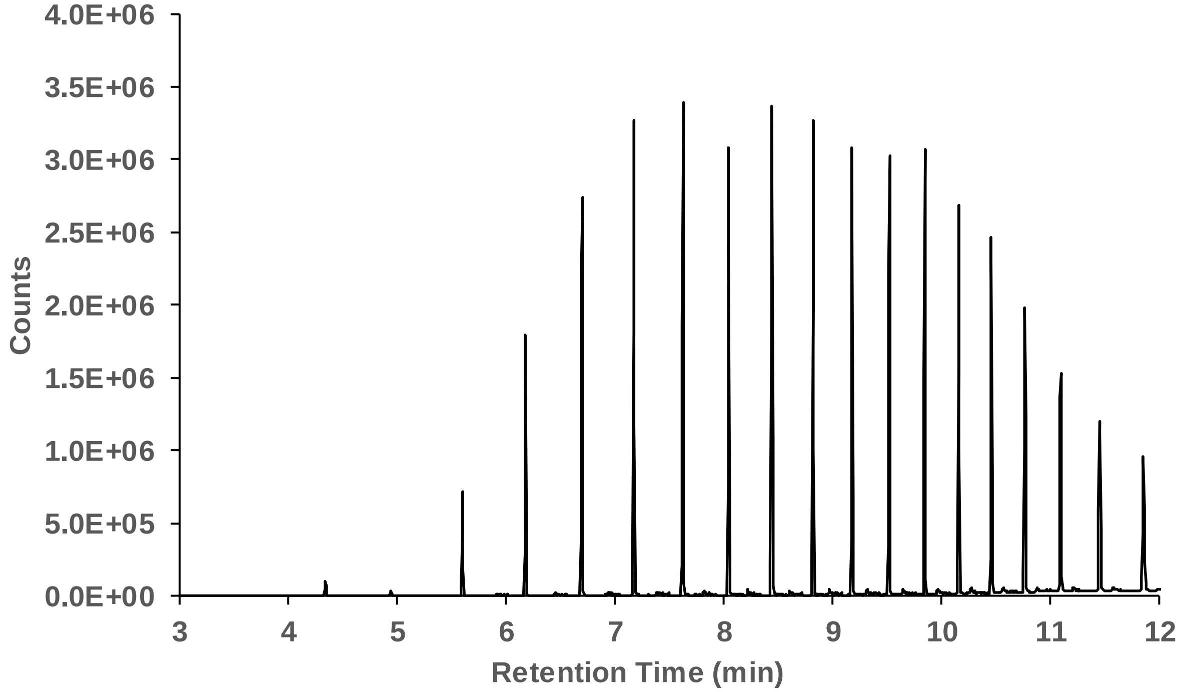

Fig. S16. GC/MS chromatogram of DCM extract from blue HDPE food container cap hydrogenolysis. Reaction details shown in Table 1, Entry 6.

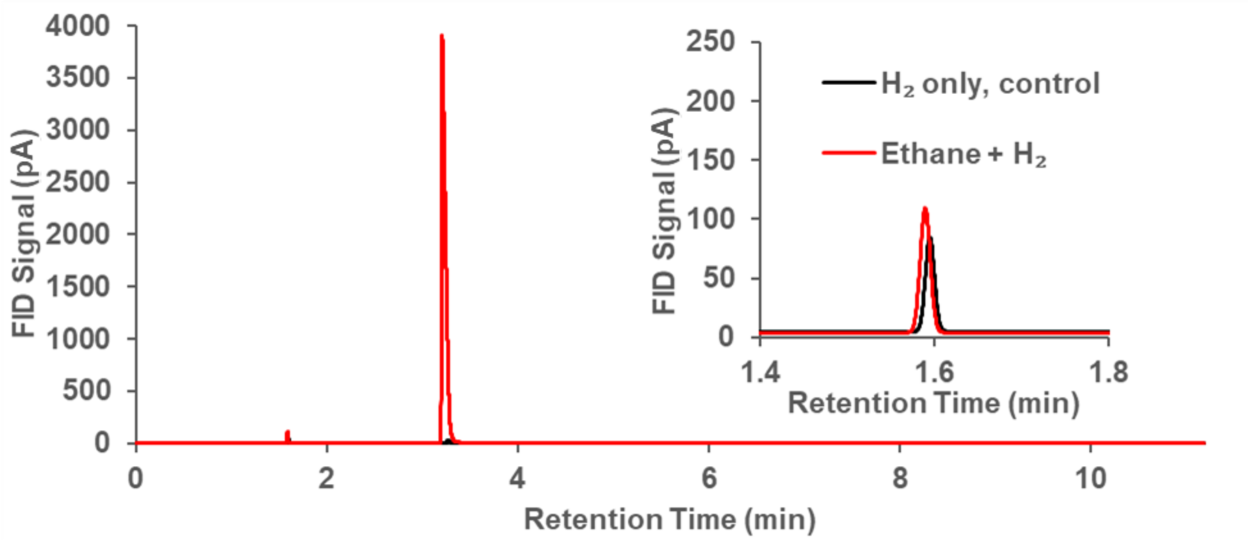

Fig. S17. GC/FID chromatogram of reactor headspace after attempted ethane hydrogenolysis Retention times: CH_4 – 1.6 min, C_2H_6 – 3.2 min. Insert: detail showing the methane peak in the control and experiment chromatograms.

Supplementary Tables

Table S1. **Quantum Chemically Computed Energy Values for ß-Alkyl Transfer and σ-Bond Metathesis Mechanisms.** Computed energy values (kcal/mol) associated with the de-polymerization process catalyzed by **AlS/ZrH$_2$** adsorbed species following two different pathways: *ß*-alkyl transfer and σ-bond metathesis mechanisms.

	**Δ*E***	**Δ*H***	**Δ*G***
**β-alkyl transfer mechanism**			
**AlS/ZrH$_2$**	0.0	0.0	0.0
**H$_2$ elimination TS**	1.5	-0.2	16.0
**AlS/ZrH-*sec*-decyl**	-9.0	-12.1	-5.2
***ß*-alkyl transfer TS**	16.2	11.4	20.9
**AlS/ZrH-nonyl**	21.0	14.8	9.3
**nonyl hydrogenolysis TS**	22.4	17.3	20.3
**AlS/ZrH$_2$ + nonane**	24.9	21.9	9.9
**propylene insertion + propyl hydrogenolysis**	-14.7	-10.9	-14.3
**σ-bond metathesis mechanism**			
**AlS/ZrH$_2$**	0.0	0.0	0.0
**σ-bond metathesis TS**	59.8	59.8	76.3
**AlS/ZrH-propyl + nonane**	-20.7	-20.0	-18.7
**propyl hydrogenolysis TS**	-9.0	-7.5	3.3
**Final products**	-14.7	-10.9	-14.3

Table S2. GPC Data for Solid Fractions of PE Hydrogenolysis Reactions. Number-average molecular weights of solids fractions for polyethylene hydrogenolysis experiments as measured by gel permeation chromatography (GPC) (Figure 2D).

**Sample**	**M_n (kg·mol^{-1})**	**M_n (-CH_2- Units)**
**Starting PE**	11.62	830
**10 min**	7.79	556
**30 min**	4.43	316
**50 min**	2.52	180
**120 min**	<0.86	<60

Supplementary Note 1

Video S1. Video of Complete Hexadecane Hydrogenolysis in 18 min. Catalytic hydrogenolysis of 1.93 mL of hexadecane over 0.178 mg of **AlS/ZrNp$_2$** (0.05 mol% Zr) at 150°C/2.5 atm H_2 (350 mL vessel) to volatile alkanes in 18 min (Table 1 Entry 7). Timer is in minutes.

DFT Cartesian Coordinates (Å) of All Species Involved in the Depolymerization Process

AlS/ZrNp$_2$

Al	0.735904649	4.995301190	8.406894477
Al	4.846794525	7.125368770	7.061935135
O	3.425897795	7.344699658	11.054700091
O	7.715735622	4.993303660	9.603111618
O	3.362482335	6.953627558	8.310522829
O	7.711821509	5.080357688	6.957550611
Al	4.835264551	3.234310028	9.845151464
Al	0.669099191	1.217100479	11.322928461
O	7.636372421	0.960542217	7.200991817
O	3.580402398	3.161604134	8.397209058
O	7.571435530	1.224414581	10.025521388
O	3.431696587	3.054806887	10.968574898
Al	7.782140965	3.158941887	9.880466367
Al	3.497392495	1.165850395	11.049371308
O	4.911588642	0.863861408	7.299544162
O	0.703677984	3.155999265	8.549807531
O	5.007719338	1.305054440	9.995214696
O	0.640657991	3.050163825	11.202416305
Al	3.497522158	4.997675146	8.264351265
Al	7.702989239	7.163673724	7.085568908
O	0.853248605	7.268504848	11.350018802
O	4.837789405	5.064063200	9.680609522
O	0.706748306	6.938172035	8.326734343
O	4.902439880	5.239304213	7.040588736
Al	6.274433199	1.056930287	8.583075728
Al	6.240422371	0.880683651	11.398041503
Al	6.309229099	6.028401235	9.772419543
O	6.300795295	7.108032572	11.199163170
O	6.316269932	7.195077132	8.402321377
Al	2.157459777	7.081447956	9.764344494
Al	2.068914760	7.049738205	6.946143335
Al	2.146274574	2.122650440	8.420743077
O	1.939872639	0.968990811	7.046571553
O	2.007177023	0.971361131	9.902200433
Al	2.055431045	4.088997538	11.173524233
O	2.086366148	5.230358641	9.756700710
O	2.035619119	5.159041351	7.121401906
Al	6.155362196	4.023479441	7.107814406
O	6.308437397	2.980044009	8.558026289
O	6.306216338	3.147190244	11.125368904
H	0.963336739	1.098796193	6.921637832
H	4.971163153	1.445240942	6.521237864
H	7.255276810	1.424436978	6.420573716
H	6.300308265	6.495993085	5.162386857
H	3.514091838	6.361506021	5.227976459
H	0.609047304	7.709229193	5.056940124
H	5.824361971	3.114826734	4.906025852
H	8.263833294	4.870630844	6.175163287
O	6.307358586	7.189595056	5.835637697
O	3.493518687	7.182580121	5.747048550
O	0.621418691	7.033376724	5.751039170
O	6.234422634	2.904009563	5.755244519

Al	9.143056880	4.987690173	8.408733199
Al	13.251429011	7.117100959	7.061845316
O	11.849647216	7.340974083	11.036649697
O	16.121788419	5.000837730	9.604190545
O	11.773545416	6.947906010	8.313552790
O	16.125254020	5.081219032	6.957867493
Al	13.237246008	3.240619568	9.889630144
Al	9.017019451	1.167658101	11.304822335
O	16.046156200	0.962531585	7.204420192
O	11.983878102	3.155265875	8.476761755
O	15.979786491	1.275218746	10.064495292
O	11.907465040	3.055921714	11.116320925
Al	16.203797806	3.176246618	9.873262372
Al	11.922280064	1.150586319	11.106054782
O	13.321576143	0.869119870	7.351142055
O	9.113574181	3.150945060	8.553266852
O	13.453109991	1.299378298	10.039938204
O	9.100014335	2.985758366	11.196742729
Al	11.901944547	4.998751987	8.281878225
Al	16.116498954	7.166779158	7.083873203
O	9.265783577	7.254882795	11.353484415
O	13.255687901	5.056215758	9.690844066
O	9.122174697	6.934396925	8.328380071
O	13.310789509	5.231305257	7.046209412
Al	14.706683761	1.077278316	8.598087288
Al	14.682677446	1.064430676	11.447971531
Al	14.713329637	6.036957855	9.771975521
O	14.644374174	7.115808366	11.214179142
O	14.724192930	7.194611540	8.401859633
Al	10.550554204	7.078347454	9.782399499
Al	10.478937646	7.048106513	6.953292189
Al	10.546592462	2.119038967	8.456294259
O	10.346765102	0.961550544	7.104215878
O	10.433404417	0.957895101	9.933109707
Al	10.482384763	4.066697289	11.189485519
O	10.503588920	5.203527298	9.760809417
O	10.451308391	5.152460335	7.125042807
Al	14.573723162	4.028263538	7.111885751
O	14.719063936	2.984408692	8.568113024
O	14.717653023	3.140749967	11.193240189
H	9.368033193	1.093492800	6.971395066
H	13.351708139	1.464416084	6.581390741
H	15.658143170	1.431321132	6.429179671
H	14.714440220	6.500857181	5.157845422
H	11.916960090	6.361722624	5.226132992
H	9.022165022	7.709966304	5.061209252
H	14.226739654	3.106778252	4.915237873
H	16.680806012	4.870788589	6.177879364
O	14.718458808	7.188592580	5.837136020
O	11.904367378	7.181359581	5.747674217
O	9.035356975	7.031647817	5.752907375
O	14.646833205	2.903592123	5.761350524
Al	0.753936884	13.056224340	8.424406887
Al	4.856513884	15.155098631	7.079237953
O	3.432809482	15.472454217	11.049122010
O	7.708373193	13.066067655	9.681166180

O	3.369496828	15.007632560	8.326380468
O	7.715528748	13.143209372	7.026999893
Al	4.882814714	11.274982863	9.997649089
Al	0.552078694	9.165293455	11.173700038
O	7.632189077	9.031435976	7.206415117
O	3.582092849	11.209424451	8.564232402
O	7.632019836	9.285344454	10.078438735
O	3.501694521	11.049863743	11.197181637
Al	7.770083435	11.243400826	9.994187883
Al	3.569444392	9.160309383	11.202651723
O	4.910214527	8.953268431	7.359384399
O	0.698552328	11.212907652	8.562713174
O	5.061474015	9.294567567	10.087741679
O	0.700858914	10.988407019	11.202450873
Al	3.531071117	13.048288839	8.363548415
Al	7.698182145	15.229663941	7.108226955
O	0.791065390	15.330063147	11.283190687
O	4.894360406	13.072257621	9.757298662
O	0.718057736	14.991835022	8.321954933
O	4.900825379	13.257847093	7.110735007
Al	6.299648111	9.131386305	8.614343161
Al	6.374678812	9.052818298	11.426720440
Al	6.305607186	14.164082646	9.910227410
O	6.069684905	15.259591883	11.213398868
O	6.315908167	15.249910112	8.404641540
Al	2.161931221	15.160133552	9.792249819
Al	2.088125523	15.074791911	6.957213661
Al	2.154524894	10.191770688	8.477815111
O	1.884497008	9.041854976	7.123202489
O	2.037848793	9.024231915	9.995200777
Al	2.080753690	12.104486045	11.209481713
O	2.098393863	13.252879591	9.791655586
O	2.081869828	13.168632101	7.177032645
Al	6.175229036	12.086640647	7.211974995
O	6.316462673	11.019798079	8.649991225
O	6.310991398	11.139801567	11.351381491
H	0.899764292	9.178953470	7.026475735
H	4.971761516	9.560589944	6.598934654
H	7.243612333	9.518675225	6.440842234
H	6.320916273	14.562954842	5.151689007
H	3.524390481	14.310499028	5.288770855
H	0.647624588	15.746430042	5.048775007
H	5.821481493	11.213060210	5.003031889
H	8.259531871	12.911313020	6.244842279
O	6.309682343	15.243732060	5.838222942
O	3.501111711	15.165950546	5.750292852
O	0.626828920	15.087997114	5.758702910
O	6.236375373	10.971505150	5.841946847
Al	9.127967750	13.045072162	8.466658442
Al	13.259163684	15.162240045	7.076503523
O	11.824855040	15.427503279	11.035823560
O	16.122142200	13.062330704	9.608491538
O	11.781592086	15.001478702	8.317667980
O	16.128451570	13.142480336	6.959568273
Al	13.241662518	11.283135518	9.943506022
Al	9.099076933	9.207323900	11.334449256

O	16.043460845	9.030368704	7.205426863
O	11.978496899	11.209083093	8.552398819
O	15.971968736	9.277573163	10.013724887
O	11.919462089	11.075048432	11.192337315
Al	16.204308592	11.240472386	9.884908161
Al	11.957733574	9.184037566	11.163968906
O	13.317825928	8.950146131	7.353850742
O	9.106491176	11.214482421	8.637749400
O	13.461409616	9.346295106	10.072128183
O	9.117045035	11.046482329	11.283126128
Al	11.904805686	13.043224524	8.332533027
Al	16.125764025	15.236969531	7.090913786
O	9.179855117	15.254714795	11.297806501
O	13.267656158	13.091609595	9.763630188
O	9.136390971	14.994377271	8.340024794
O	13.316665978	13.250429626	7.112631071
Al	14.689107927	9.133996225	8.592884881
Al	14.680223687	9.050444445	11.458080072
Al	14.713187100	14.115104933	9.803290007
O	14.573546207	15.252164726	11.211475684
O	14.728321819	15.257261357	8.408990955
Al	10.552986017	15.151574317	9.759400941
Al	10.483599140	15.067824812	6.956427054
Al	10.535371590	10.183177078	8.493404287
O	10.294740585	9.043884450	7.122814442
O	10.453882171	9.030545452	9.986407156
Al	10.511849403	12.116930023	11.206542334
O	10.514093223	13.257155572	9.785315313
O	10.448458864	13.163901144	7.182816094
Al	14.597447125	12.075340234	7.154911349
O	14.720101456	11.041167513	8.621060275
O	14.740117227	11.140829190	11.209488745
H	9.309945031	9.175688519	7.017970144
H	13.376595208	9.548646628	6.585969207
H	15.661679272	9.514640923	6.433627098
H	14.728337853	14.578300819	5.147345840
H	11.933321831	14.332770504	5.260041295
H	9.031738942	15.759182097	5.067388132
H	14.314539947	11.125976064	4.953050615
H	16.685830686	12.932418988	6.181426478
O	14.718399017	15.248961507	5.843298930
O	11.910457354	15.169720220	5.753051832
O	9.036256386	15.087289436	5.764792188
O	14.645649690	10.902392183	5.833435042
O	7.778560972	9.123708921	12.629960379
H	7.946641652	8.554121704	13.407284718
O	12.159175305	10.249259712	14.561705907
H	14.862012413	11.713446071	11.990345325
O	13.235327527	9.115829207	12.615276772
O	10.884151534	9.192666326	12.718509593
S	12.074639015	9.053326749	13.772924747
O	12.142878317	7.758008018	14.388437510
O	-0.694022407	9.118365441	12.556576367
H	-0.512536689	8.601221982	13.364625568
O	0.707851236	15.172649767	13.759061774
O	-0.629520931	13.631687289	12.403560309

O	1.797048055	13.379082419	12.481961410
S	0.636795463	14.363387766	12.568597255
O	9.113656461	15.159669906	13.762416691
O	7.868850066	13.478101647	12.482962917
O	10.285722009	13.395705417	12.486826482
S	9.097529023	14.332323020	12.587810253
H	15.052937177	14.769789750	11.941528130
H	4.356300442	15.100964105	11.134299723
H	12.615014383	14.888086299	11.196899592
O	0.867772872	7.112377902	13.836586568
O	-0.554123974	5.578954951	12.551913170
O	1.874900602	5.325765699	12.488583786
S	0.724955658	6.293194096	12.660222633
O	9.274309104	7.098696112	13.838335835
O	7.865061582	5.568145993	12.553079622
O	10.294805130	5.312012153	12.491761911
S	9.143189029	6.285913897	12.656363879
H	6.790600291	6.614150781	11.919025910
H	4.197126542	6.782379664	11.234365047
H	12.606940067	6.759407421	11.201473998
H	15.148681141	6.635883744	11.932941906
O	7.646824228	1.121752914	12.561854277
H	7.768388680	0.548301959	13.341092666
O	3.503385429	0.877854009	14.731840336
O	11.991755303	-0.085251337	14.404291835
O	-0.707373530	1.124097760	12.574154036
H	-0.547418416	0.592778386	13.377933480
O	3.502815771	3.149221103	13.757918778
H	6.391514407	3.685456028	11.932084083
O	4.675206180	1.283307919	12.565836399
O	2.318885759	1.288669195	12.559396943
S	3.496386964	1.718766383	13.576407420
O	11.998297334	2.420220620	14.398816570
H	14.827900057	3.714171651	11.973652728
O	13.145482229	1.129161305	12.569584263
O	10.760435032	1.201368029	12.625570756
S	11.946316342	1.164624972	13.704007012
O	4.605500119	10.887340678	13.837493264
O	2.789990773	9.363508341	13.040834554
O	4.989319238	8.802781350	12.637485449
O	4.200493384	8.787053187	15.027595833
S	4.119150178	9.473747066	13.775405840
Zr	6.429842826	12.079745278	13.331076972
C	7.531461960	11.611014280	15.082802172
H	7.219457290	12.666592905	15.344189261
H	6.642852918	11.011269337	15.410400986
C	8.726010919	11.231375208	15.960038179
C	8.352348715	11.415530159	17.447563065
H	7.502845616	10.775845585	17.729378719
H	9.206504169	11.148329487	18.087548614
H	8.080973741	12.459443055	17.663934052
C	9.914759454	12.144185567	15.629966484
H	9.652426307	13.202653471	15.771113422
H	10.771733766	11.912716867	16.276556653
H	10.244146802	12.006862726	14.595652953
C	9.078259581	9.759103530	15.715888880

H	9.377647440	9.606160839	14.676070503
H	9.915986911	9.440917680	16.350876999
H	8.221426386	9.103470628	15.936005169
C	5.044538498	13.608397022	14.150320043
H	5.421752974	14.587863241	13.801259953
H	4.163612889	13.421155972	13.505917553
C	4.566219135	13.755060092	15.647305807
C	5.402471011	14.867132046	16.306035073
H	5.112259242	15.002035270	17.358823700
H	6.475300964	14.613511044	16.289336891
H	5.267176627	15.826745327	15.792804929
C	3.070528243	14.121969164	15.632209723
H	2.857429970	14.960847667	14.970966594
H	2.478697694	13.258514145	15.293899870
H	2.725842276	14.388667324	16.643583885
C	4.666159251	12.504788763	16.551163338
H	4.189903484	11.625755702	16.102288011
H	5.695640476	12.249075891	16.821360715
H	4.138154933	12.717698079	17.492592590

AlS/Zr(H)Np

Al	0.736442433	4.992266522	8.413455944
Al	4.846590379	7.121663483	7.064619700
O	3.418800984	7.353843049	11.119766946
O	7.715989519	4.999169396	9.604465031
O	3.363985889	6.952578839	8.320092751
O	7.712025899	5.079950375	6.958984164
Al	4.850946359	3.257532563	9.922616211
Al	0.698354066	1.166756912	11.377554582
O	7.638511881	0.959941080	7.274426176
O	3.573555623	3.157329854	8.479988499
O	7.576028917	1.230923934	10.094499241
O	3.495901687	3.068797258	11.118505705
Al	7.781184783	3.166837687	9.912089583
Al	3.526437536	1.173725585	11.104614808
O	4.909838919	0.873511430	7.366893686
O	0.703821881	3.150886881	8.553231491
O	5.054342622	1.355775509	10.077714366
O	0.692193437	2.993573749	11.201846009
Al	3.500800615	4.989097335	8.278779620
Al	7.704057385	7.162166263	7.085412804
O	0.853987952	7.266516976	11.350530362
O	4.836375551	5.073003842	9.685247572
O	0.707562341	6.935533969	8.328883977
O	4.900971440	5.232680259	7.044875587
Al	6.277687691	1.065768843	8.652381011
Al	6.263442633	0.848390905	11.490571197
Al	6.307982237	6.031783883	9.775735195
O	6.238207358	7.110569532	11.214140929
O	6.317321977	7.195436979	8.404282071
Al	2.175575124	7.076753306	9.787383305
Al	2.068339292	7.047007374	6.955206559
Al	2.145415717	2.107050733	8.441494575
O	1.941669208	0.970062520	7.050963135
O	2.027570107	0.966588164	9.920911633
Al	2.078637984	4.071524864	11.187597631

O	2.090853947	5.221974796	9.767117215
O	2.041107966	5.154405530	7.128032292
Al	6.159496065	4.018273871	7.123282530
O	6.311095390	2.982657983	8.579562093
O	6.319286958	3.218695778	11.186126352
H	0.966528918	1.105009601	6.920773321
H	4.978687324	1.458858472	6.591445479
H	7.260575927	1.414525243	6.487672224
H	6.299922850	6.491760314	5.166309104
H	3.511017165	6.358710296	5.235068407
H	0.614109750	7.705686338	5.058048761
H	5.792927254	3.101245716	4.928459601
H	8.259131501	4.867470409	6.174031549
O	6.306863210	7.187972240	5.837078448
O	3.493106157	7.181251989	5.752171484
O	0.622848053	7.031241766	5.753509725
O	6.234855793	2.902685405	5.764437342
Al	9.143287527	4.982069776	8.410154656
Al	13.252950804	7.115225117	7.064004803
O	11.845035096	7.337857330	11.035093770
O	16.124260449	4.993511148	9.612045540
O	11.773920534	6.946730740	8.314131581
O	16.125213863	5.080068486	6.960068643
Al	13.245371993	3.239605312	9.897940748
Al	9.036501131	1.181364059	11.348785661
O	16.046131302	0.961164086	7.208566005
O	11.987379963	3.153317237	8.482695872
O	15.986734155	1.227201699	10.084238099
O	11.912063890	3.059009740	11.123250551
Al	16.223461630	3.150444351	9.889814064
Al	11.936180901	1.153921715	11.146558859
O	13.324672842	0.866633281	7.358994118
O	9.112011349	3.148439067	8.560546950
O	13.462113061	1.295415294	10.076056777
O	9.110361789	2.995225328	11.205618605
Al	11.904528321	4.996856753	8.285299314
Al	16.117851436	7.164387952	7.085111831
O	9.265252047	7.248482482	11.355478611
O	13.264008828	5.059559120	9.698623691
O	9.122854512	6.933622017	8.328528204
O	13.311802834	5.228059170	7.049049473
Al	14.709549670	1.066845041	8.615452034
Al	14.695951023	1.049405974	11.480078046
Al	14.723356961	6.044169662	9.783157892
O	14.643783974	7.161734020	11.213051785
O	14.725548513	7.194033436	8.403996923
Al	10.542457935	7.077998217	9.777135403
Al	10.480284129	7.045623187	6.952747039
Al	10.547175589	2.116522898	8.485875632
O	10.354852634	0.963943165	7.119638642
O	10.436836233	0.958332781	9.984281433
Al	10.491085691	4.078401619	11.208422177
O	10.504017520	5.203281488	9.767603234
O	10.452571817	5.150687317	7.127929011
Al	14.575745224	4.023162905	7.121137196
O	14.721340756	2.976807334	8.579816598

O	14.727783201	3.137874527	11.199257622
H	9.375665265	1.092506495	6.995555178
H	13.360084089	1.468044527	6.594127253
H	15.653236865	1.428501813	6.435188612
H	14.715167383	6.497842658	5.161147506
H	11.920000064	6.362965370	5.224298032
H	9.023374595	7.708214042	5.060606292
H	14.211562977	3.106994999	4.925935586
H	16.679137481	4.866859503	6.179848211
O	14.718754387	7.187922338	5.838186691
O	11.905722344	7.180762007	5.748696455
O	9.035720145	7.030906249	5.753302966
O	14.647030851	2.903765480	5.764288615
Al	0.754705823	13.068808107	8.458223032
Al	4.852561566	15.171336079	7.114005320
O	3.491582001	15.474092604	11.044046413
O	7.709191251	13.063052733	9.690070546
O	3.366362770	15.007094348	8.334102524
O	7.718296454	13.149327816	7.041396388
Al	4.873194995	11.272208491	9.971135690
Al	0.548723122	9.162059776	11.175353888
O	7.632195811	9.031432557	7.206743011
O	3.580765882	11.211220819	8.560473842
O	7.631782265	9.286056575	10.081054155
O	3.508142387	11.057872338	11.188480266
Al	7.785876728	11.234743078	9.979108308
Al	3.562913845	9.183219091	11.203085122
O	4.910482940	8.952481284	7.359365725
O	0.696425314	11.218325906	8.566130714
O	5.061382955	9.296984987	10.082662286
O	0.701876709	10.982980815	11.203894467
Al	3.531060809	13.053757418	8.368674020
Al	7.702851775	15.232871872	7.177210786
O	0.854661880	15.318649133	11.357164744
O	4.898287624	13.070619713	9.759208712
O	0.715805307	14.990543830	8.328181574
O	4.903754862	13.282107077	7.117151951
Al	6.301209463	9.132132027	8.612052954
Al	6.371336208	9.057747576	11.426745383
Al	6.307629231	14.161802877	9.949801804
O	6.084069222	15.241225965	11.273146049
O	6.318840527	15.255694381	8.470773893
Al	2.200840007	15.160046598	9.829375889
Al	2.084721970	15.073482198	6.964868772
Al	2.149124632	10.194619355	8.477678935
O	1.884451488	9.042488195	7.124342210
O	2.040883426	9.023190083	9.996653291
Al	2.080495990	12.102897534	11.239711288
O	2.101543788	13.259067577	9.828985930
O	2.078796115	13.166886970	7.193440620
Al	6.174851636	12.100345677	7.210747624
O	6.316718313	11.026000327	8.648252256
O	6.311804283	11.137359679	11.346553049
H	0.899604800	9.177009772	7.025065275
H	4.970468910	9.558053087	6.597411309
H	7.244958912	9.521368660	6.442125891

H	6.321884628	14.577305161	5.213549191
H	3.543220990	14.310648420	5.307849637
H	0.650818472	15.744861760	5.050900487
H	5.834492455	11.215117750	4.999313659
H	8.269791093	12.939481902	6.258732900
O	6.313876765	15.250865252	5.906746313
O	3.503755245	15.170675570	5.759421053
O	0.627567399	15.087136523	5.761305390
O	6.235590328	10.974686871	5.845324625
Al	9.144573067	13.047005136	8.493648427
Al	13.268710997	15.160191666	7.079373814
O	11.838298918	15.427922831	11.047366006
O	16.131738013	13.065221436	9.674431499
O	11.816191535	14.999498720	8.330424847
O	16.127149982	13.142374393	6.970004497
Al	13.243522631	11.286462941	9.945801810
Al	9.123028541	9.216350579	11.364962269
O	16.043933867	9.029953919	7.205889448
O	11.983261467	11.212470031	8.553566251
O	15.970721005	9.282079403	10.016928597
O	11.919611633	11.076967046	11.188891085
Al	16.207768976	11.239107537	9.893250286
Al	11.948013750	9.176305009	11.135462646
O	13.318745507	8.950192093	7.355980722
O	9.110011328	11.215633557	8.636346887
O	13.461029523	9.350771896	10.073424184
O	9.114857344	11.052597996	11.283537838
Al	11.921861576	13.047243116	8.343559112
Al	16.128896080	15.236228588	7.096488518
O	9.186042467	15.249872833	11.365073552
O	13.302320134	13.088443630	9.764211366
O	9.175784331	14.997433097	8.397835542
O	13.318647633	13.248419184	7.110634869
Al	14.693700981	9.134019744	8.594935149
Al	14.666402094	9.088686397	11.473929682
Al	14.727517693	14.131345436	9.838075934
O	14.578952312	15.244941685	11.274328682
O	14.732772819	15.255063130	8.423480309
Al	10.566026886	15.150700809	9.786056403
Al	10.501925031	15.074276311	6.984283560
Al	10.540047753	10.185994674	8.489408212
O	10.295982895	9.044465950	7.121304823
O	10.451654160	9.028813541	9.970922582
Al	10.515111476	12.123115025	11.252077287
O	10.520722082	13.258895357	9.831805380
O	10.454765697	13.168474765	7.200558864
Al	14.598921327	12.074310381	7.157061027
O	14.723320402	11.043979210	8.627365484
O	14.737960016	11.136708540	11.229400816
H	9.311425374	9.175500125	7.017156395
H	13.380064084	9.547115789	6.586839635
H	15.661652227	9.513818401	6.433918848
H	14.732410322	14.568018004	5.159981182
H	11.928753422	14.328106425	5.273415198
H	9.018544728	15.752704301	5.125314463
H	14.318693286	11.128121128	4.954307160

H	16.707675223	12.917861503	6.213006141
O	14.720073101	15.248934388	5.846094187
O	11.910674036	15.170217739	5.757979876
O	9.034073385	15.091181017	5.832570083
O	14.646059447	10.901363785	5.835269040
O	7.775013410	9.117966746	12.629546622
H	7.952334418	8.535713048	13.396121469
O	12.066122984	10.567633343	14.321566828
H	14.856434804	11.714063721	12.007719293
O	13.172534888	9.113928132	12.624347542
O	10.813444638	9.201526056	12.648297577
S	11.983723441	9.253051847	13.745332287
O	11.995526529	8.073458853	14.557434662
O	-0.698451249	9.118961830	12.560450655
H	-0.515943852	8.611074192	13.373829320
O	0.859646963	15.174223459	13.843904872
O	-0.621927078	13.710622901	12.555107408
O	1.793648181	13.314266312	12.554772164
S	0.695065885	14.366253325	12.666906987
O	9.119224821	15.158967911	13.831303936
O	7.871379070	13.479983341	12.559333342
O	10.285303700	13.387676831	12.561554423
S	9.104603402	14.338414443	12.651343862
H	15.098954123	14.771941403	11.990986100
H	4.405565526	15.096818460	11.192575734
H	12.613803572	14.878747502	11.243123172
O	0.868730813	7.111941092	13.835914899
O	-0.556177874	5.579325133	12.550390906
O	1.874217294	5.320431251	12.491778843
S	0.724098504	6.289879924	12.661574492
O	9.269995022	7.106101315	13.842920702
O	7.862522265	5.571591234	12.552187609
O	10.295135171	5.305937831	12.541169401
S	9.141563231	6.287513610	12.664640395
H	6.748878575	6.625012354	11.926062530
H	4.228326416	6.833647799	11.266282217
H	12.614919729	6.765187408	11.168454709
H	15.129843707	6.681925475	11.940855635
O	7.701125629	1.124641814	12.629331454
H	7.840517844	0.558656522	13.411790160
O	3.498779769	0.162609754	14.563770253
O	11.988938480	-0.079936834	14.471378702
O	-0.703303123	1.123706552	12.626414749
H	-0.564912477	0.627800441	13.454296068
O	3.504800961	2.647004826	14.236585367
H	6.444823714	3.782825636	11.968813351
O	4.692974706	1.142561507	12.630631746
O	2.329649370	1.210191544	12.573586620
S	3.501271106	1.307752930	13.705339209
O	11.995998547	2.416737146	14.463259738
H	14.847489452	3.717624948	11.973969360
O	13.154819219	1.130188413	12.631458661
O	10.776852412	1.201830749	12.651584509
S	11.952665996	1.164923784	13.757622384
O	4.587131641	10.974058235	13.913879235
O	2.729494439	9.514228707	13.044327988

O	4.936313954	8.947284865	12.615282817
O	4.128904989	8.796847567	14.974791288
S	4.058679528	9.572926523	13.778912002
H	5.163167405	13.539040583	13.506834760
Zr	6.307516789	12.144591883	13.191006947
C	7.463555561	11.362243102	14.901552179
H	7.235978189	10.282072895	14.967854301
H	8.524463326	11.428714478	14.586447807
C	7.269364186	12.020255435	16.289398837
C	8.284135852	11.414054907	17.277991459
H	9.315710394	11.573390374	16.932139788
H	8.181647594	11.874805057	18.273305201
H	8.127342857	10.330906810	17.386066304
C	5.842650612	11.763376079	16.810026313
H	5.608939616	10.689089786	16.826380466
H	5.735922562	12.152990223	17.833348997
H	5.082614332	12.260228916	16.189518971
C	7.507052178	13.538380542	16.190545255
H	6.786744047	14.023306638	15.508035196
H	7.378443207	14.016808798	17.172967349
H	8.519401149	13.766729272	15.834361305

AlS/ZrH$_2$

Al	0.735428046	4.991430541	8.413384859
Al	4.845432555	7.120335252	7.062488569
O	3.414597423	7.353955326	11.117903251
O	7.716250573	4.998542191	9.604340202
O	3.363259846	6.952458219	8.319890416
O	7.712060961	5.079871636	6.958799031
Al	4.850724415	3.256657737	9.922394321
Al	0.694520554	1.169965410	11.374991934
O	7.638371698	0.959909769	7.274343185
O	3.573513521	3.157048075	8.479655109
O	7.575371932	1.229677963	10.094518996
O	3.496122187	3.068824818	11.118379170
Al	7.781859220	3.165840563	9.912168551
Al	3.525956347	1.174053917	11.104920137
O	4.909546143	0.869359227	7.366841265
O	0.703634564	3.150766071	8.552967577
O	5.054194931	1.354619271	10.077449501
O	0.691808925	2.994761388	11.201359683
Al	3.499907644	4.988848960	8.278696043
Al	7.705500570	7.160149425	7.083300138
O	0.853537005	7.266495882	11.352434860
O	4.835263511	5.072097956	9.684947498
O	0.707922427	6.935704856	8.329845003
O	4.900774286	5.232509546	7.045038958
Al	6.276696880	1.066384379	8.650776977
Al	6.263207748	0.847468726	11.489444661
Al	6.307130944	6.027153723	9.771725619
O	6.237451038	7.103729241	11.211358234
O	6.317197315	7.193690005	8.403212918
Al	2.171278753	7.075948850	9.786050189
Al	2.068034647	7.046719486	6.954810065
Al	2.145155275	2.107473743	8.440954395
O	1.941490235	0.969873070	7.050737744

O	2.027916407	0.967391166	9.919999365
Al	2.078766966	4.071014715	11.187301712
O	2.090336091	5.221153061	9.766916442
O	2.040441881	5.154399253	7.128032865
Al	6.159470270	4.018033299	7.122160749
O	6.310736704	2.982541423	8.578822589
O	6.319249131	3.218602521	11.185289680
H	0.966190588	1.105092710	6.920955410
H	4.973196950	1.454977329	6.591347188
H	7.260247755	1.414841555	6.487765224
H	6.300544553	6.494829988	5.162458628
H	3.508879350	6.359525280	5.232666598
H	0.611801737	7.704617247	5.057996617
H	5.798580971	3.101839521	4.925523540
H	8.260462677	4.866016560	6.175074235
O	6.306816245	7.187691280	5.836686816
O	3.492352823	7.180998856	5.751503650
O	0.622288896	7.031217572	5.754431005
O	6.234973442	2.902492334	5.764175887
Al	9.144536376	4.983049617	8.411286113
Al	13.252169308	7.116173405	7.066129259
O	11.845613102	7.338486412	11.035328688
O	16.123877767	4.993629386	9.612393376
O	11.773707414	6.947612740	8.314313282
O	16.125102330	5.080345180	6.960485380
Al	13.243983221	3.238931360	9.897381415
Al	9.038044320	1.181380918	11.351666460
O	16.046033847	0.961200778	7.208397245
O	11.986593042	3.153421725	8.482588191
O	15.985750956	1.228673931	10.083040544
O	11.911143336	3.059344868	11.123090896
Al	16.222255074	3.151381814	9.889328402
Al	11.935306949	1.154350871	11.147232342
O	13.324674533	0.866167865	7.358978915
O	9.112143930	3.148720578	8.560670950
O	13.461330336	1.295563839	10.075959012
O	9.110441431	2.995488552	11.205312092
Al	11.904885786	4.998122097	8.286504931
Al	16.116696787	7.163815972	7.087819197
O	9.263178243	7.252489032	11.358645535
O	13.262217719	5.057295211	9.696652829
O	9.122188581	6.933940013	8.327885995
O	13.311832132	5.229237360	7.048820413
Al	14.709309086	1.067401550	8.615791875
Al	14.694955836	1.051536312	11.480891758
Al	14.720650442	6.041998875	9.795082818
O	14.636984403	7.120080442	11.256687869
O	14.726045239	7.194193292	8.411470862
Al	10.541482063	7.082335742	9.779306983
Al	10.480702458	7.045655613	6.953081061
Al	10.547044337	2.116259548	8.486255399
O	10.354888442	0.963804382	7.119739503
O	10.436498705	0.957516605	9.984415339
Al	10.490519910	4.080583654	11.208361824
O	10.503025136	5.209289307	9.768756548
O	10.452828003	5.150721651	7.128364234

Al	14.574854162	4.023811869	7.121684969
O	14.721161835	2.976839147	8.579532469
O	14.727147865	3.138294941	11.199222525
H	9.375911324	1.092639385	6.995327659
H	13.360961355	1.467123017	6.593716557
H	15.653050383	1.429072309	6.435308279
H	14.714115797	6.495151338	5.166169851
H	11.921246957	6.363260055	5.224773983
H	9.024957847	7.707668843	5.059547954
H	14.213178892	3.107211546	4.925513509
H	16.678436769	4.868149419	6.179567470
O	14.718752319	7.188373615	5.840041470
O	11.905941117	7.181089327	5.749038125
O	9.036273764	7.030757399	5.752637169
O	14.647043478	2.903962572	5.764701124
Al	0.748181363	13.068725453	8.462530081
Al	4.852554732	15.163107507	7.112326331
O	3.489683458	15.474488036	11.042675549
O	7.708588448	13.060768260	9.687212973
O	3.366087019	15.004420031	8.331058719
O	7.718321732	13.148543244	7.041055148
Al	4.866949615	11.267615160	9.950897260
Al	0.551632090	9.167352062	11.189719508
O	7.632826061	9.029814969	7.205532465
O	3.576797821	11.209632699	8.558893787
O	7.631556260	9.281918010	10.081778360
O	3.508172901	11.063008309	11.187375926
Al	7.790313124	11.226476421	9.965625765
Al	3.556905207	9.184695401	11.195531037
O	4.909758584	8.950992446	7.357377526
O	0.691973498	11.218114280	8.571005603
O	5.057600464	9.291583755	10.076369189
O	0.706116426	10.991403533	11.210598196
Al	3.524247663	13.051900586	8.364942039
Al	7.703906585	15.233086929	7.176065592
O	0.850317807	15.328126915	11.352905371
O	4.897574297	13.069765362	9.757120547
O	0.714748023	14.992386592	8.329522929
O	4.899913272	13.260286547	7.116127698
Al	6.301704829	9.124392309	8.605612281
Al	6.360104784	9.028992304	11.407090171
Al	6.307547386	14.162598408	9.941689524
O	6.085515998	15.238179830	11.270543577
O	6.318269517	15.254548249	8.467855863
Al	2.198618710	15.163180552	9.826335305
Al	2.081950398	15.073470996	6.963869884
Al	2.142199818	10.195209563	8.479013356
O	1.883595851	9.042829168	7.124709243
O	2.037510148	9.025256882	9.997975984
Al	2.081421128	12.120611166	11.253910796
O	2.100473075	13.263375204	9.830761939
O	2.064202331	13.166534157	7.193720186
Al	6.179917448	12.091285413	7.208998062
O	6.316958957	11.007123759	8.639870268
O	6.307381479	11.146858347	11.292837542
H	0.898745348	9.175893994	7.023644885

H	4.971525348	9.555156843	6.594371170
H	7.245962333	9.519180380	6.440272978
H	6.325511802	14.582468863	5.206911189
H	3.539802148	14.313691847	5.298909939
H	0.647716203	15.746807486	5.051872921
H	5.838440906	11.216417088	4.995704441
H	8.270202350	12.939402401	6.258354887
O	6.313223965	15.249016038	5.906830244
O	3.503681863	15.168962575	5.759563524
O	0.626994507	15.087551046	5.760986183
O	6.236611215	10.972371988	5.841981216
Al	9.146175021	13.047488913	8.493599006
Al	13.268665374	15.159678933	7.080083060
O	11.838074978	15.427867856	11.047655245
O	16.129912387	13.065980638	9.676987250
O	11.815933017	14.998694022	8.331244726
O	16.125903638	13.142279817	6.971672398
Al	13.246230503	11.285767956	9.955449648
Al	9.115772532	9.215461643	11.388227631
O	16.043549936	9.029322327	7.209968713
O	11.985108124	11.211278062	8.555628989
O	15.966619660	9.280707886	10.046424632
O	11.918062082	11.075502588	11.190436921
Al	16.203916070	11.241504499	9.909550351
Al	11.945285691	9.176323238	11.138293451
O	13.319830124	8.950288151	7.358071674
O	9.111383414	11.214690254	8.634522617
O	13.463943837	9.347579875	10.078717412
O	9.111140479	11.050687101	11.283919087
Al	11.922788149	13.044633558	8.344704260
Al	16.127834237	15.236409514	7.096695478
O	9.184609840	15.248104005	11.368358644
O	13.303008016	13.087172402	9.765363414
O	9.175533817	14.997079980	8.397926609
O	13.318919893	13.247514175	7.111511924
Al	14.693681083	9.133032595	8.603205972
Al	14.664240726	9.068293300	11.488482841
Al	14.725689139	14.132437952	9.839834653
O	14.577253357	15.245685459	11.274715026
O	14.732621217	15.255119498	8.424692120
Al	10.566589068	15.149222847	9.786945066
Al	10.502662048	15.073773726	6.984886474
Al	10.542572757	10.182821264	8.493833431
O	10.297012233	9.044528716	7.122257345
O	10.449758959	9.029536490	9.977469868
Al	10.512545247	12.120123174	11.255405981
O	10.520262505	13.255750746	9.832700194
O	10.455335330	13.168242748	7.200803523
Al	14.599004860	12.072705723	7.161150801
O	14.724475364	11.043019616	8.634386369
O	14.734331444	11.135360590	11.258297526
H	9.312810718	9.176212509	7.017865427
H	13.382766648	9.547458816	6.589234923
H	15.659401305	9.514469410	6.439539250
H	14.732637875	14.565513264	5.162318428
H	11.929513297	14.328431853	5.272905762

H	9.021214371	15.752585597	5.125006179
H	14.317330084	11.128469049	4.957496039
H	16.711166275	12.914716076	6.219128271
O	14.719952005	15.248625744	5.846284842
O	11.910772905	15.170010365	5.758373958
O	9.034396619	15.091100698	5.832418886
O	14.646305123	10.900959229	5.837654059
O	7.722883836	9.122075887	12.630322569
H	7.873070957	8.590385779	13.436007985
O	12.066844057	10.567827691	14.321618221
H	14.846407978	11.713933955	12.036024567
O	13.169040130	9.113205155	12.626240871
O	10.808187907	9.201851392	12.649848977
S	11.981089874	9.253195771	13.749326881
O	11.994643584	8.072158198	14.558363061
O	-0.696703902	9.117180580	12.569042606
H	-0.511450497	8.617258193	13.386626264
O	0.800128350	15.232676094	13.834168164
O	-0.622406356	13.710917841	12.554362608
O	1.798416180	13.365521969	12.559048272
S	0.679371538	14.394771700	12.668096616
O	9.115236237	15.154769132	13.836564228
O	7.866588808	13.475498114	12.561337510
O	10.284128728	13.387556994	12.561298588
S	9.105328188	14.336401744	12.656259097
H	15.089488997	14.770819586	11.994973493
H	4.400704354	15.092949795	11.191970607
H	12.612877344	14.879067590	11.246855479
O	0.871064962	7.111828543	13.836910490
O	-0.550505091	5.574471661	12.554341956
O	1.876947749	5.319971369	12.492019435
S	0.728066383	6.289557630	12.662997976
O	9.270427691	7.106676319	13.845200459
O	7.861513779	5.572591379	12.551910289
O	10.293905955	5.307118368	12.540795929
S	9.140537410	6.289556993	12.668809411
H	6.756794110	6.619526983	11.920766447
H	4.220131852	6.829025534	11.266715752
H	12.608685246	6.762531935	11.193210498
H	15.146494851	6.640477865	11.970433196
O	7.700009399	1.124743869	12.628823954
H	7.838246308	0.566892728	13.417024296
O	3.497816900	0.162380047	14.563075074
O	11.988604231	-0.079914595	14.470954381
O	-0.705071930	1.126533655	12.624482965
H	-0.558175358	0.618691977	13.444715474
O	3.504848311	2.646836472	14.236386146
H	6.444624394	3.781139507	11.969201630
O	4.693504103	1.142264801	12.629967106
O	2.330031725	1.210396119	12.572666415
S	3.500480080	1.307838188	13.704924969
O	11.995647600	2.417242588	14.462597765
H	14.845182742	3.715260017	11.976188056
O	13.153396689	1.130366151	12.630770934
O	10.774294092	1.201800043	12.651239030
S	11.951863064	1.164998220	13.758224600

O	4.598804839	10.973766222	13.913145621
O	2.726163362	9.517937897	13.044598669
O	4.920242274	8.953124881	12.587426351
O	4.130804337	8.795039229	14.966604809
S	4.058955131	9.571176515	13.772236123
H	5.080162752	13.534111470	13.354901678
Zr	6.259536603	12.165634662	13.091743260
H	7.246432843	11.582529588	14.525278531

H_2

H	10.479096141	3.155846212	18.632680369
H	10.479095928	3.155846082	17.882048429

dodecane

C	7.055973588	11.407335705	14.995174799
H	6.333347654	11.586563543	15.805296736
H	7.007979479	10.337560808	14.742764902
C	8.469928043	11.810990696	15.417939310
H	8.498909834	12.892990104	15.630421914
H	9.167925766	11.649205677	14.579268915
C	8.973325677	11.044762006	16.644568459
H	8.277443142	11.207949586	17.486325927
H	8.941894713	9.961083095	16.434360071
C	10.389241262	11.435887146	17.074867364
H	10.420026814	12.519094418	17.286893381
H	11.084287501	11.274149572	16.232285138
C	10.892097422	10.666938946	18.299450907
H	10.861333264	9.583713394	18.087824633
H	10.197288451	10.828935438	19.142105109
C	12.308050315	11.058230369	18.729860535
H	13.003415388	10.896257936	17.887674175
H	12.338246269	12.141516691	18.941340161
C	12.810006818	10.290116233	19.955187174
H	12.786076417	9.207071098	19.741730116
H	12.110657806	10.446823696	20.795126093
C	14.221797179	10.688224309	20.393283267
H	14.921716931	10.535622397	19.553091656
H	14.244158712	11.770439301	20.611131243
C	14.723009189	9.915577386	21.616095413
H	14.704655594	8.833731700	21.395933299
H	14.019503724	10.064001040	22.454135049
H	6.717128928	11.972578313	14.115659879
C	16.131207001	10.316881569	22.062177360
H	16.835660654	10.171496857	21.224296943
H	16.149398816	11.398015117	22.286216319
C	16.632199761	9.540069931	23.283054552
H	16.619573873	8.460951688	23.054882406
H	15.923376826	9.680772088	24.116418686
C	18.036139537	9.956020628	23.726834889
H	18.067379373	11.022861760	23.994420402
H	18.770004656	9.797571285	22.922433480
H	18.372599201	9.383044158	24.602358229

Propylene

C	6.687972193	11.388906622	14.909063676

C	7.105488037	12.467473167	15.580238440
H	6.986878770	11.211199189	13.875763611
H	6.033785118	10.643123329	15.367560748
H	7.759946598	13.177195403	15.065006016
C	6.745767279	12.791220859	17.009007158
H	5.656461822	12.860688703	17.143976250
H	7.107866086	12.014567407	17.698920765
H	7.180920698	13.745647637	17.330588570

Propane

C	10.938394548	5.937519803	17.387775194
H	10.391280908	6.690852643	17.974346244
C	11.841200073	5.089818154	18.287179684
C	11.054314880	4.323211472	19.352743305
H	10.323028808	3.643435293	18.889993070
H	11.713256418	3.719136965	19.992542798
H	10.495347022	5.011908100	20.004019838
H	10.189645002	5.312085530	16.878526071
H	12.587998365	5.737179541	18.774661184
H	12.413386403	4.378671748	17.669751706
H	11.512155702	6.468046913	16.614532671

Nonane

C	10.364726821	11.467836451	17.122949348
H	10.311223407	12.538107185	17.372987577
H	11.044643020	11.365833015	16.263781521
C	10.857152709	10.646778836	18.316619088
H	10.871842438	9.576662803	18.049545966
H	10.141993025	10.740713872	19.151060288
C	12.249231358	11.066290863	18.796237604
H	12.960995027	10.987717926	17.955511511
H	12.229644506	12.135037918	19.073555092
C	12.775765880	10.248724923	19.978558048
H	12.799409142	9.179697148	19.703352315
H	12.069375676	10.327450230	20.823441614
C	14.169145023	10.685081215	20.438675325
H	14.871116055	10.610187043	19.589609051
H	14.142299991	11.754462114	20.712419335
C	14.717075497	9.876948787	21.617711856
H	14.747197000	8.807335289	21.345515179
H	14.018251222	9.952404098	22.469179400
C	16.110856470	10.327126892	22.063273756
H	16.807292484	10.253079250	21.209493619
H	16.078728548	11.397731706	22.331897310
C	16.674265173	9.529278304	23.242627342
H	16.708502831	8.460067756	22.973776794
H	15.979770336	9.604765726	24.096224790
C	18.067218539	9.997728397	23.669245676
H	18.054030646	11.055298345	23.972749669
H	18.787482900	9.902565644	22.842817037
H	18.452969670	9.412884318	24.516084442
H	9.364351941	11.151577156	16.795787588

H_2 elimination TS

Al	0.736741471	4.991083632	8.412702184

Al	4.846363894	7.121131012	7.062717214
O	3.417704601	7.354035249	11.120463567
O	7.716074976	4.999176063	9.604833581
O	3.363626263	6.952249279	8.320084468
O	7.711960677	5.079778558	6.958927665
Al	4.851268061	3.257023036	9.922002544
Al	0.698945342	1.166025357	11.376535747
O	7.638526593	0.959181258	7.274625825
O	3.573320805	3.157538814	8.479925750
O	7.577501508	1.233893312	10.093277669
O	3.496162550	3.068603666	11.118615082
Al	7.781003732	3.165971453	9.911098659
Al	3.528028891	1.172205783	11.103811801
O	4.909674356	0.870004708	7.367281212
O	0.704325606	3.150239865	8.553092813
O	5.054411731	1.357194409	10.077493701
O	0.692142943	2.993382302	11.201934681
Al	3.501242260	4.988784905	8.278224921
Al	7.704231161	7.161187591	7.084107994
O	0.853819200	7.266211286	11.351457006
O	4.836442105	5.073206244	9.685543362
O	0.707504522	6.935271457	8.328970816
O	4.900823058	5.232451941	7.044607994
Al	6.276502072	1.062335379	8.652400529
Al	6.265438553	0.849869493	11.488842537
Al	6.308275189	6.031942704	9.776159444
O	6.237629551	7.113380233	11.214329278
O	6.317396402	7.193618509	8.403496025
Al	2.174339983	7.075676865	9.785332987
Al	2.068339400	7.047350125	6.954946908
Al	2.146133051	2.105973883	8.440983002
O	1.941809017	0.969902316	7.050608977
O	2.027994600	0.966031299	9.921019012
Al	2.079027558	4.070419426	11.186586857
O	2.090411924	5.221621054	9.767162555
O	2.041054599	5.154416270	7.127858071
Al	6.159008948	4.017209247	7.123298591
O	6.310880271	2.982551607	8.579536383
O	6.319382761	3.218501430	11.186630810
H	0.966618728	1.104441101	6.920834885
H	4.977221004	1.453810373	6.590857917
H	7.259040436	1.411882016	6.487715174
H	6.300162219	6.490393748	5.166170430
H	3.509344020	6.357776761	5.235044011
H	0.614053184	7.706355423	5.058674096
H	5.788197324	3.099652461	4.930088308
H	8.258433749	4.866451647	6.173778924
O	6.306853011	7.187258449	5.836305091
O	3.492523494	7.180765538	5.751502346
O	0.622781898	7.031170372	5.753431672
O	6.234867793	2.902902378	5.763955346
Al	9.142901893	4.982215644	8.409805854
Al	13.252924483	7.115071216	7.063205798
O	11.844483627	7.338096837	11.034850846
O	16.125025486	4.991741575	9.611129645
O	11.774968587	6.946159488	8.313023701

O	16.125192171	5.079951668	6.959835578
Al	13.244092018	3.236395634	9.896626637
Al	9.034043465	1.179905202	11.349427022
O	16.046162085	0.960948670	7.208510574
O	11.986251128	3.151851024	8.482272588
O	15.986713511	1.226886542	10.083951322
O	11.911573476	3.058206257	11.123492953
Al	16.223874922	3.149146701	9.889235004
Al	11.934970885	1.152428316	11.146439039
O	13.324624199	0.866355564	7.358947909
O	9.112249117	3.148849537	8.560622635
O	13.461783522	1.295123661	10.076085983
O	9.110276471	2.995289341	11.206034930
Al	11.904426737	4.994849740	8.283594663
Al	16.117910181	7.164086785	7.085095422
O	9.264765072	7.250191458	11.355929493
O	13.260719230	5.054249846	9.694924811
O	9.123182186	6.933322256	8.327972363
O	13.312171001	5.227705015	7.048304641
Al	14.709841128	1.065940857	8.614591624
Al	14.696239494	1.048445048	11.479222538
Al	14.720570059	6.035230233	9.776806199
O	14.641096730	7.127034298	11.218727178
O	14.725308744	7.192884490	8.404086613
Al	10.545740448	7.078257753	9.771989032
Al	10.480181240	7.045794970	6.951593024
Al	10.545930918	2.115153877	8.485759020
O	10.354474213	0.963785855	7.119520382
O	10.435959535	0.957406810	9.985696081
Al	10.490871551	4.077916691	11.206410684
O	10.503754757	5.205123415	9.767708575
O	10.452650124	5.150539186	7.127706387
Al	14.575798984	4.022250979	7.120376721
O	14.721390045	2.976112742	8.579242864
O	14.727450214	3.137675404	11.198897024
H	9.374870010	1.091064162	6.995902234
H	13.359384300	1.467441599	6.593872347
H	15.653154449	1.427406020	6.434748667
H	14.715306908	6.497356133	5.161415203
H	11.920010149	6.363013272	5.223462817
H	9.022843278	7.709390592	5.061450572
H	14.210339264	3.106305569	4.925838016
H	16.679138153	4.866888597	6.179547405
O	14.718834469	7.187838482	5.838053058
O	11.905815165	7.180646157	5.748093992
O	9.035915929	7.030424379	5.752524481
O	14.647060378	2.903637871	5.763655800
Al	0.755550766	13.068659351	8.459699884
Al	4.854509489	15.162905152	7.116211344
O	3.491213986	15.474256581	11.043746066
O	7.708111502	13.063700594	9.683006442
O	3.367082524	15.005486183	8.332820787
O	7.718033097	13.148603023	7.040659939
Al	4.880562099	11.277035853	9.973302833
Al	0.548416949	9.164576178	11.173570252
O	7.632935825	9.029379958	7.205415978

O	3.580310121	11.209014034	8.561391212
O	7.635219410	9.280079677	10.078517196
O	3.508145370	11.050708695	11.186599583
Al	7.775097453	11.238172686	9.976971725
Al	3.562887635	9.183755416	11.185420390
O	4.909563586	8.950470210	7.357629768
O	0.695230561	11.217975644	8.566362103
O	5.059088176	9.287142979	10.079269381
O	0.703751955	10.984000115	11.204409381
Al	3.533806679	13.048920646	8.367436109
Al	7.701574311	15.230728549	7.180098806
O	0.854357998	15.318435798	11.357502065
O	4.894940831	13.070980200	9.749700191
O	0.716186909	14.990423116	8.328398475
O	4.901791496	13.263220723	7.113053422
Al	6.299611651	9.125977251	8.608176723
Al	6.369317862	9.072546586	11.414477886
Al	6.302970937	14.156046174	9.960146785
O	6.089571043	15.247671173	11.273091321
O	6.317669762	15.253300507	8.472326638
Al	2.203020062	15.157563877	9.828234106
Al	2.085786821	15.071886027	6.964680346
Al	2.148906429	10.196549493	8.475487407
O	1.883760276	9.042200531	7.124084390
O	2.038784692	9.021725622	9.994889526
Al	2.089215882	12.104275082	11.241376158
O	2.101308839	13.257944847	9.829575895
O	2.077618295	13.165905245	7.193686490
Al	6.178202445	12.090626362	7.213070389
O	6.316933706	11.002980003	8.637830819
O	6.315549350	11.137151978	11.287361232
H	0.898982506	9.176657111	7.025112563
H	4.972194950	9.557232296	6.596650059
H	7.245461376	9.520916438	6.441740175
H	6.323192130	14.572768817	5.217064147
H	3.543393382	14.309933600	5.306570433
H	0.651447840	15.745724483	5.051662750
H	5.836288230	11.218833081	4.997764622
H	8.266938109	12.940853734	6.255663060
O	6.313489141	15.249349517	5.907426694
O	3.504544412	15.169241743	5.759745566
O	0.627762326	15.087140216	5.761238780
O	6.236541387	10.972783798	5.842496277
Al	9.142121410	13.046306310	8.492038170
Al	13.268316375	15.159851239	7.079486955
O	11.837682158	15.427801294	11.046868474
O	16.131330233	13.065758644	9.674402567
O	11.816021142	14.999395728	8.330499615
O	16.127075564	13.142436909	6.969875654
Al	13.245590217	11.286052945	9.945668413
Al	9.114288669	9.214859992	11.344294379
O	16.043834679	9.029673679	7.205894291
O	11.983985399	11.212377946	8.553513133
O	15.969891678	9.278734523	10.017461954
O	11.920054011	11.073588337	11.187749508
Al	16.207525210	11.240714278	9.894989011

Al	11.945550861	9.176929380	11.130921716
O	13.318660129	8.950007579	7.355647434
O	9.109503896	11.215558221	8.636569469
O	13.461827066	9.346165622	10.073141452
O	9.116138768	11.048661985	11.281965813
Al	11.921250811	13.047490487	8.343522789
Al	16.129548575	15.235417705	7.096584546
O	9.183053319	15.245867534	11.369502952
O	13.301674410	13.088227719	9.764382985
O	9.176675259	14.997692595	8.397886706
O	13.318811775	13.248303486	7.110800575
Al	14.693778883	9.134029417	8.593574839
Al	14.666707995	9.068811717	11.469903888
Al	14.726719079	14.131385942	9.838080212
O	14.579117800	15.244986525	11.274458064
O	14.732751029	15.255099638	8.423578097
Al	10.564062418	15.148949759	9.786477093
Al	10.501297424	15.073760122	6.984364825
Al	10.539370093	10.188969458	8.482862214
O	10.295194472	9.043990268	7.119260833
O	10.452879818	9.026400199	9.957328389
Al	10.515992156	12.120885452	11.246387727
O	10.520888661	13.257846094	9.830219293
O	10.454833444	13.167996383	7.200198746
Al	14.599457595	12.074564850	7.157035105
O	14.723374795	11.043188896	8.626916349
O	14.737861158	11.136145311	11.223176730
H	9.309894314	9.173575955	7.016865830
H	13.380433496	9.547173849	6.586649264
H	15.661806959	9.514055452	6.434005045
H	14.732746743	14.567542104	5.160538794
H	11.928524970	14.327670987	5.273992466
H	9.016480020	15.753939737	5.127247941
H	14.319243089	11.128399120	4.954303802
H	16.709872390	12.916954198	6.214823700
O	14.720149786	15.248856580	5.846250131
O	11.910663445	15.170098342	5.758013826
O	9.033618856	15.090980369	5.833070303
O	14.646058390	10.901166623	5.835349111
O	7.770250327	9.125298428	12.635860195
H	7.953853941	8.503026852	13.371109153
O	12.068146735	10.565619645	14.320817967
H	14.853626479	11.712650999	12.002348913
O	13.173938185	9.112811339	12.622731485
O	10.815106892	9.201310879	12.648437968
S	11.986267227	9.251413286	13.741063143
O	11.995899408	8.073719357	14.557338501
O	-0.699552485	9.117463843	12.559568831
H	-0.515057058	8.603086807	13.368608450
O	0.859079171	15.174099216	13.843876753
O	-0.622572269	13.710766498	12.555392690
O	1.792915428	13.314653828	12.555757186
S	0.694732225	14.366026119	12.666244984
O	9.038790031	15.084975286	13.836046858
O	7.878958446	13.403851038	12.478930820
O	10.289368414	13.385645667	12.557606648

S	9.076721683	14.299545342	12.630850119
H	15.100704129	14.771433089	11.989979257
H	4.395667246	15.083026959	11.207104089
H	12.609414103	14.875372758	11.247523249
O	0.869397669	7.112265341	13.835872226
O	-0.553458690	5.577094791	12.552427911
O	1.875317277	5.320176965	12.492006810
S	0.726216750	6.289925540	12.660917014
O	9.268425911	7.107148195	13.842166880
O	7.862909306	5.571496360	12.552369415
O	10.295380960	5.307302656	12.540975849
S	9.141778942	6.287320426	12.662841037
H	6.744261471	6.627863916	11.927466364
H	4.231943520	6.838097420	11.257586435
H	12.617087392	6.768249817	11.168145519
H	15.140279528	6.648251781	11.939944865
O	7.702227571	1.123569014	12.632127298
H	7.835488022	0.528538330	13.395050800
O	3.498664064	0.162734259	14.563904040
O	11.987849989	-0.080323364	14.471575492
O	-0.703247094	1.123691051	12.626291860
H	-0.563638875	0.624008970	13.451722110
O	3.504540535	2.646450505	14.237011101
H	6.444786048	3.783015337	11.968931638
O	4.692141280	1.142760406	12.631241908
O	2.329639887	1.210290680	12.573789888
S	3.501523183	1.307511925	13.704096807
O	11.995957009	2.416170334	14.463644782
H	14.846138285	3.717013433	11.973924400
O	13.153586027	1.130130174	12.631779852
O	10.774677711	1.200236797	12.653882708
S	11.951565745	1.164536171	13.756999350
O	4.587036513	10.967728563	13.921421979
O	2.727329423	9.513605638	13.046794053
O	4.937080141	8.949553548	12.618969212
O	4.128873527	8.795714241	14.977129383
S	4.052597395	9.562537653	13.774722468
H	4.943662031	13.326188948	12.807011749
Zr	6.253635941	12.151003050	13.200799198
C	7.324864350	11.618593768	15.305491245
H	6.866066072	10.636967500	15.052964099
C	7.007741985	11.882729540	16.786446348
H	7.403161358	12.876650331	17.061597544
H	7.570424084	11.152896644	17.394959457
C	5.517319873	11.803599280	17.126890092
H	4.955036613	12.515407875	16.498677577
H	5.134936752	10.802233605	16.867545614
H	6.704687827	12.812260241	14.941350708
H	6.102842392	13.592553718	14.452121098
C	8.822827542	11.573624686	15.012915354
H	9.316698252	10.834178960	15.662039700
H	9.053015883	11.282596333	13.979314746
H	9.296307884	12.546200269	15.201729898
C	5.228137784	12.100113124	18.599338135
H	5.784997631	11.387212997	19.232854284
H	5.618964438	13.101309584	18.851585076

C	3.739822118	12.035793919	18.951423841
H	3.349838617	11.032330930	18.708558853
H	3.183708407	12.741284425	18.310674788
C	3.455751412	12.351141055	20.421809364
H	4.024494813	11.652103350	21.060500346
H	3.841328512	13.358546919	20.658313473
C	1.973090596	12.279715090	20.794982669
H	1.402493666	12.985423857	20.167047427
H	1.582595926	11.276112774	20.552593656
C	1.707361555	12.583945604	22.271612858
H	2.093633214	13.590529050	22.510825166
H	2.287619584	11.881933878	22.896744310
C	0.229730118	12.503480817	22.661888379
H	-0.158483932	11.499158069	22.417628875
H	-0.351444833	13.208993385	22.043071068
C	-0.028104136	12.798123411	24.142452191
H	0.557841540	12.093745424	24.757465458
H	0.358328521	13.803098187	24.382999834
C	-1.506621731	12.710171295	24.527448070
H	-1.909221980	11.706131290	24.326129633
H	-2.109755545	13.426097643	23.949595990
H	-1.663811701	12.925797594	25.594333245

AlS/ZrH-*sec*-dodecyl

Al	0.736871185	4.992889995	8.414031376
Al	4.845919989	7.121858717	7.064362120
O	3.420605619	7.354080919	11.121510655
O	7.716251062	4.998981450	9.603870138
O	3.364075063	6.952434087	8.320302227
O	7.711783594	5.079611750	6.958686075
Al	4.850513239	3.257743350	9.922241737
Al	0.696886615	1.166049191	11.374787385
O	7.638471957	0.960024981	7.274174118
O	3.573465354	3.157484076	8.479981186
O	7.576338818	1.231761493	10.094422125
O	3.495846497	3.068521023	11.118695317
Al	7.780684143	3.166883743	9.912121548
Al	3.526963612	1.172845301	11.103177352
O	4.909620864	0.873705309	7.366598060
O	0.703948911	3.150996316	8.553278268
O	5.054515487	1.356559254	10.077356322
O	0.692353942	2.993385254	11.201878827
Al	3.500533427	4.989527903	8.278944498
Al	7.702304658	7.161702082	7.084046455
O	0.854249995	7.266837484	11.350357407
O	4.836514197	5.072898815	9.685217441
O	0.707374434	6.935503822	8.328341650
O	4.900671768	5.232713971	7.044726141
Al	6.277402772	1.065242533	8.651273974
Al	6.264075206	0.848783904	11.487937175
Al	6.308107633	6.032028680	9.776048251
O	6.238500711	7.111285947	11.213148609
O	6.317593268	7.194276357	8.403445010
Al	2.176787566	7.077963009	9.788600222
Al	2.068212709	7.047523752	6.955703645
Al	2.145347851	2.107139237	8.441038063

O	1.941606920	0.970201601	7.050854504
O	2.027645105	0.966447459	9.920962624
Al	2.078585378	4.071856364	11.187572351
O	2.091209249	5.222639980	9.767281888
O	2.041086446	5.154363128	7.128064971
Al	6.158060015	4.017770664	7.123330207
O	6.311070186	2.982640422	8.579519541
O	6.319241428	3.218532707	11.186449714
H	0.966318928	1.104705906	6.921141536
H	4.977978938	1.458611220	6.590862872
H	7.260369193	1.414250694	6.487346048
H	6.298340009	6.491947298	5.165118930
H	3.509876253	6.357760460	5.236040830
H	0.614653712	7.705459609	5.057678296
H	5.791462623	3.100569759	4.928818008
H	8.256527428	4.867811130	6.171851834
O	6.306480288	7.187659927	5.836380469
O	3.492802523	7.181014125	5.752041542
O	0.623322535	7.030845609	5.752930588
O	6.234822481	2.902846014	5.764241918
Al	9.141648317	4.975565963	8.405140687
Al	13.251342403	7.112802945	7.058472591
O	11.843700437	7.335256533	10.966513392
O	16.124386254	4.993443411	9.611649245
O	11.772372803	6.944079000	8.302322330
O	16.125546065	5.079713142	6.959665296
Al	13.245788531	3.240330359	9.897233811
Al	9.037373914	1.180181432	11.344689608
O	16.046400576	0.961337005	7.208476292
O	11.987779367	3.153069632	8.482291960
O	15.987290114	1.227299152	10.084165455
O	11.912673257	3.059244038	11.123074391
Al	16.223502732	3.151221454	9.889864319
Al	11.937552796	1.154545913	11.145873906
O	13.324579617	0.871734447	7.358779999
O	9.111293250	3.146365932	8.559988415
O	13.462776516	1.295466602	10.076098294
O	9.110178105	2.994464109	11.205750224
Al	11.903918040	4.996455973	8.282617404
Al	16.117938024	7.161187482	7.082850084
O	9.266266802	7.252380112	11.354003154
O	13.263695579	5.060305023	9.697785368
O	9.122432646	6.931834472	8.326875323
O	13.311828732	5.226058326	7.047844212
Al	14.710842593	1.066902591	8.615671504
Al	14.696482554	1.048296869	11.479719631
Al	14.723804758	6.045119505	9.779626177
O	14.645204894	7.164500957	11.205236862
O	14.726360016	7.192889113	8.399861640
Al	10.519625637	7.074599568	9.747094056
Al	10.482256867	7.042974735	6.942223730
Al	10.548132800	2.116487710	8.485187316
O	10.354288041	0.964424452	7.119238438
O	10.438028686	0.959511563	9.983018031
Al	10.490639736	4.077016753	11.205278469
O	10.501446185	5.193908828	9.761784482

O	10.450577788	5.148776313	7.124977210
Al	14.576450918	4.022764742	7.120883199
O	14.721630889	2.976910083	8.579978435
O	14.727927288	3.137969972	11.199067122
H	9.374789827	1.093320964	6.996266794
H	13.364314360	1.473662372	6.594310610
H	15.654162926	1.428393302	6.434680333
H	14.718172849	6.498198270	5.156966301
H	11.921272925	6.365142255	5.209673710
H	9.020796263	7.709085196	5.060698389
H	14.212176872	3.107735976	4.925658248
H	16.680571851	4.864839580	6.180646106
O	14.719193055	7.186331869	5.836033050
O	11.905335357	7.179510651	5.739112344
O	9.034423803	7.030139864	5.751817147
O	14.647093603	2.903535840	5.764055001
Al	0.755144521	13.068715548	8.458205901
Al	4.852511062	15.170944316	7.113857612
O	3.490174480	15.474528692	11.043826838
O	7.709041903	13.065042413	9.688500977
O	3.366002388	15.007424330	8.333831202
O	7.717859659	13.149788021	7.040903690
Al	4.876230922	11.272143412	9.966309639
Al	0.549895639	9.164574690	11.174496429
O	7.632264755	9.030280964	7.206141885
O	3.580822588	11.211151553	8.559773377
O	7.634599753	9.282896305	10.077981585
O	3.510232520	11.055907882	11.186778121
Al	7.781888909	11.235789820	9.973512336
Al	3.567731727	9.183206533	11.204000661
O	4.910143721	8.951852595	7.358632795
O	0.697797863	11.218182303	8.564786018
O	5.060510921	9.290898722	10.080813147
O	0.701920127	10.984270526	11.202748732
Al	3.532096006	13.053742746	8.368760171
Al	7.702260010	15.232306686	7.176890126
O	0.854361798	15.318961858	11.357129163
O	4.896808825	13.071345102	9.755262486
O	0.715736518	14.990521671	8.328316903
O	4.903819029	13.282444859	7.115924358
Al	6.300255131	9.130592535	8.611166486
Al	6.376446659	9.066123921	11.420906071
Al	6.304795210	14.161912705	9.948888064
O	6.085505254	15.243850311	11.271313839
O	6.318457240	15.256355555	8.470177531
Al	2.200267981	15.158467523	9.828964302
Al	2.084942945	15.073223836	6.964852164
Al	2.150509099	10.194009231	8.478501744
O	1.885433697	9.042351333	7.124417286
O	2.041437592	9.022654268	9.996616356
Al	2.084693592	12.102716193	11.241135081
O	2.101376699	13.258405467	9.829087967
O	2.078747145	13.167184735	7.193212611
Al	6.174293614	12.099430462	7.208701141
O	6.316788775	11.020070634	8.640033456
O	6.315693387	11.139428253	11.296644672

H	0.901036856	9.177268935	7.024561336
H	4.970546191	9.557800960	6.596951208
H	7.245065528	9.522323142	6.442690520
H	6.321430159	14.576275579	5.214210488
H	3.543718915	14.310218026	5.308624831
H	0.651805903	15.745993182	5.051451038
H	5.844267784	11.216016667	4.993530787
H	8.270832232	12.940518552	6.259112586
O	6.313735921	15.251111252	5.906165693
O	3.503690264	15.170696134	5.759318896
O	0.627924207	15.087443780	5.761075067
O	6.235527351	10.974174818	5.843675632
Al	9.143376411	13.050609458	8.493154050
Al	13.268462814	15.168777729	7.076236975
O	11.839177618	15.428750463	11.047292266
O	16.131050525	13.064757221	9.673586998
O	11.815988020	15.002433085	8.329171795
O	16.127798169	13.144398494	6.966809594
Al	13.245767890	11.278133339	9.892900619
Al	9.124401392	9.222526504	11.353426298
O	16.045744120	9.028920964	7.201353345
O	11.989736145	11.215867123	8.485792578
O	15.972436010	9.283550196	10.008752185
O	11.914374514	11.058596369	11.116591859
Al	16.208492900	11.239207037	9.884648669
Al	11.946739329	9.157983234	11.071428409
O	13.318113348	8.946578747	7.343491615
O	9.110253312	11.218618585	8.634312888
O	13.464663366	9.348111110	10.017475274
O	9.114100956	11.054653758	11.281693316
Al	11.920746465	13.053932985	8.329382930
Al	16.128881069	15.235848249	7.095934578
O	9.191724812	15.257155638	11.363413996
O	13.300244241	13.084064654	9.756816335
O	9.175051808	14.999475146	8.397938119
O	13.320876412	13.267830496	7.102783915
Al	14.697649249	9.134138330	8.571997597
Al	14.669868670	9.091299007	11.441567217
Al	14.724681870	14.129083299	9.834376180
O	14.578678580	15.243501728	11.272714174
O	14.732844737	15.256882137	8.422896753
Al	10.568241557	15.151958684	9.783788165
Al	10.500284771	15.079166770	6.982558631
Al	10.540615078	10.195744360	8.464250315
O	10.292287243	9.041484010	7.113578898
O	10.443818064	9.027052088	9.929443725
Al	10.519249154	12.117787352	11.240462151
O	10.522178591	13.261025726	9.827778413
O	10.451536515	13.176682279	7.197001777
Al	14.593797664	12.084298825	7.132731386
O	14.727214201	11.044394036	8.588061825
O	14.729869367	11.132341166	11.205535191
H	9.306607221	9.168354261	7.013762744
H	13.369493926	9.540841997	6.572166121
H	15.666302861	9.515523878	6.429421930
H	14.729611395	14.572433520	5.157125713

H	11.929188645	14.328068375	5.277007715
H	9.014572142	15.753966409	5.124491551
H	14.338903363	11.120051955	4.931044389
H	16.713554281	12.923698663	6.212560246
O	14.720933460	15.251305521	5.845109573
O	11.909529924	15.172801515	5.756843384
O	9.033158592	15.092217055	5.831479778
O	14.645978638	10.903643199	5.821799621
O	7.777726845	9.123591748	12.630271992
H	7.946319453	8.534192722	13.392956420
O	12.147354755	10.805453924	14.005380040
H	14.829861412	11.712506417	11.984155570
O	13.166297735	9.045350568	12.560577186
O	10.816672219	9.205416881	12.622675969
S	12.009597099	9.414990002	13.659744206
O	11.997303107	8.388643752	14.654963755
O	-0.702266214	9.120636384	12.554373130
H	-0.526745258	8.604940586	13.364051677
O	0.859150461	15.174235471	13.843693676
O	-0.622638693	13.710829799	12.555194265
O	1.792954703	13.314773344	12.555147523
S	0.694410542	14.365715791	12.666527843
O	9.186805509	15.161912997	13.837882541
O	7.864893315	13.541613084	12.563889973
O	10.288080826	13.382389656	12.553456565
S	9.125328120	14.351427670	12.651636610
H	15.100197143	14.769226687	11.987970540
H	4.396551216	15.086058610	11.202467194
H	12.615862640	14.881284371	11.243421254
O	0.868373576	7.112082950	13.835701473
O	-0.556796405	5.580152793	12.549997080
O	1.873920459	5.320422620	12.491780805
S	0.723700825	6.290373071	12.660992381
O	9.273078530	7.106720146	13.842008088
O	7.864961169	5.572399160	12.551934872
O	10.298081568	5.309100972	12.534911149
S	9.143235290	6.290457871	12.661375699
H	6.746141588	6.627278208	11.926999608
H	4.232776709	6.836240549	11.263018158
H	12.591215408	6.743176896	11.133109147
H	15.128944867	6.690481714	11.937094886
O	7.702479500	1.124190179	12.628998871
H	7.844605301	0.567111903	13.416302184
O	3.499658992	0.162550879	14.563613046
O	11.990819750	-0.079333114	14.472219475
O	-0.703037994	1.123513393	12.626187228
H	-0.563384988	0.624033084	13.451754226
O	3.504810945	2.646199628	14.237093300
H	6.444418186	3.784086394	11.968120053
O	4.693400561	1.142279213	12.631087231
O	2.330272646	1.209984795	12.574706932
S	3.500816781	1.307788619	13.703638233
O	11.996756734	2.416718174	14.464084833
H	14.848886057	3.718697687	11.972822760
O	13.156812077	1.130212844	12.632088847
O	10.781930457	1.202265547	12.651645934

S	11.955266230	1.165064145	13.757281282
O	4.589946229	10.975889036	13.916216415
O	2.739721502	9.510548837	13.041700979
O	4.963250492	8.951303504	12.625178011
O	4.130298467	8.797560087	14.975689191
S	4.069284052	9.573309652	13.778598377
H	4.972098257	13.496532262	13.147056362
Zr	6.260644745	12.203931422	13.137184527
C	8.736353709	11.569279227	15.259459965
H	9.292758378	12.409867156	14.817903201
C	7.237188699	11.758967717	15.066959606
H	6.687068495	10.899085874	15.484148172
C	6.639764726	13.069735711	15.548364249
H	5.899783563	13.551925716	14.811568875
H	7.405258015	13.844006234	15.691982744
C	9.143203256	11.450098238	16.738652569
H	8.602502142	10.603858181	17.196910045
H	8.807274255	12.355107513	17.273796383
H	9.068814641	10.668779797	14.717755491
C	10.649924132	11.274334088	16.931606929
H	10.972307794	10.333296056	16.459007589
H	11.180297901	12.075558391	16.390260079
H	6.012744655	12.991964753	16.445247317
C	11.092849794	11.276959980	18.395802006
H	10.765218558	12.214912380	18.878347756
H	10.584338420	10.460285462	18.938179835
C	12.608592091	11.128222464	18.555949669
H	13.110581268	11.945642789	18.009791699
H	12.935054392	10.194256912	18.066761018
C	13.076679635	11.130629457	20.013306951
H	12.586495717	10.302897711	20.555751467
H	12.735253163	12.059106757	20.504393408
C	14.594623773	11.007929300	20.164674118
H	14.937204819	10.085085369	19.665233851
H	15.081438876	11.839357690	19.625912504
C	15.078220538	11.001059876	21.616880217
H	14.605146116	10.162724567	22.159084995
H	14.736081262	11.921200843	22.123765000
C	16.599951064	10.890623012	21.742433585
H	16.937923586	9.973652098	21.231423708
H	17.067582993	11.727237653	21.196891252
C	17.089556558	10.883099874	23.191556402
H	16.662023767	10.035536899	23.748817363
H	16.793197530	11.805693596	23.713733833
H	18.184522243	10.803074597	23.250079721

β-alkyl transfer TS

Al	0.738723066	4.999025147	8.412636507
Al	4.847666105	7.127859343	7.066290792
O	3.423823975	7.361346787	11.114390340
O	7.710752633	4.997143296	9.605751801
O	3.364759845	6.954849865	8.319809105
O	7.710193296	5.078587288	6.960127595
Al	4.829994597	3.240735876	9.889012839
Al	0.668068901	1.220055075	11.352858208
O	7.638042652	0.960734047	7.272706725

O	3.581649831	3.161150539	8.416597434
O	7.567862800	1.220951936	10.089755449
O	3.438539416	3.057782849	11.031439762
Al	7.778205891	3.160812152	9.906693732
Al	3.489393329	1.165458278	11.106915440
O	4.909602248	0.873958387	7.363702040
O	0.704043499	3.156165304	8.552436485
O	5.011866979	1.308371513	10.072232196
O	0.637209983	3.055542458	11.204443287
Al	3.499069072	4.997642666	8.271676687
Al	7.700450115	7.168170451	7.086277356
O	0.858190339	7.287873154	11.338519397
O	4.843693085	5.066568772	9.688376439
O	0.705937570	6.937657924	8.326078918
O	4.902624613	5.238028049	7.044233307
Al	6.273615193	1.052628877	8.644706562
Al	6.240581866	0.875699107	11.462342142
Al	6.308193133	6.048822716	9.787479839
O	6.232606412	7.176298399	11.204275414
O	6.318855159	7.204631368	8.407719075
Al	2.170757807	7.087152770	9.788885191
Al	2.068571527	7.052258937	6.954447654
Al	2.147064230	2.117557011	8.432713946
O	1.939593139	0.971321029	7.049855669
O	2.019305295	0.971109332	9.914756608
Al	2.053044713	4.093559930	11.188946774
O	2.091055015	5.231733474	9.763120220
O	2.038598624	5.158823853	7.124861313
Al	6.153050905	4.017860190	7.127512953
O	6.306429249	2.974845277	8.587134603
O	6.305780124	3.147649258	11.188005694
H	0.963531458	1.104874046	6.923213884
H	4.971214640	1.466535885	6.593368234
H	7.258685744	1.414069017	6.485740890
H	6.296739575	6.483957024	5.174635100
H	3.512097831	6.356552058	5.239534138
H	0.614897781	7.707033851	5.055992967
H	5.782390684	3.099792335	4.935609718
H	8.252521378	4.865334035	6.172105472
O	6.306915067	7.188371419	5.836749699
O	3.493767699	7.181956172	5.751981677
O	0.622862723	7.032762091	5.751671358
O	6.234937761	2.903577614	5.766530132
Al	9.138750237	4.982605571	8.401679977
Al	13.246794983	7.115338187	7.023552543
O	11.846354101	7.341246093	10.959767782
O	16.118741157	5.004087683	9.605940200
O	11.774213486	6.949883289	8.256007737
O	16.125989736	5.080709193	6.958286093
Al	13.244848947	3.247232287	9.893672341
Al	9.020975507	1.173258121	11.368218882
O	16.045961090	0.963144686	7.207874514
O	11.984739619	3.155046039	8.482224510
O	15.981481651	1.274239051	10.080814191
O	11.910435856	3.062643357	11.121224403
Al	16.205710095	3.176665461	9.877187911

Al	11.930031429	1.174730236	11.147199219
O	13.324436808	0.878682732	7.357766467
O	9.110605259	3.148609622	8.560968009
O	13.460652413	1.300981528	10.074908207
O	9.104894200	2.989370214	11.205483052
Al	11.903390308	5.000147770	8.270025392
Al	16.118567211	7.163044691	7.083605799
O	9.259904518	7.252731785	11.288673342
O	13.258214453	5.068476946	9.691087446
O	9.122796642	6.933581198	8.320378797
O	13.312662706	5.228055071	7.042030939
Al	14.710843252	1.074125581	8.614966198
Al	14.689409863	1.059977590	11.479898820
Al	14.717648056	6.058285112	9.772953688
O	14.643773449	7.169523327	11.204174860
O	14.722640345	7.206227992	8.393795567
Al	10.527043408	7.089111002	9.725375327
Al	10.482907471	7.041452650	6.914295891
Al	10.545850274	2.117267932	8.484891715
O	10.352839138	0.966397312	7.118949613
O	10.429486528	0.962782420	9.985323711
Al	10.485695136	4.075256025	11.176121983
O	10.504885092	5.211379839	9.748885270
O	10.447831597	5.146949197	7.118380130
Al	14.577924382	4.027397561	7.113477492
O	14.718553758	2.983500461	8.572999127
O	14.719241611	3.144666651	11.199495377
H	9.372539817	1.094312971	6.996074990
H	13.370829454	1.479040568	6.592068418
H	15.656560904	1.430971720	6.433284496
H	14.736163084	6.502985408	5.143319543
H	11.915433236	6.357862476	5.164684148
H	9.008941952	7.703784160	5.048618009
H	14.219460083	3.106259779	4.919791364
H	16.685464964	4.867795256	6.181720330
O	14.722878474	7.183961580	5.829444435
O	11.903265029	7.177906066	5.685469538
O	9.030322177	7.031629392	5.746124707
O	14.646949046	2.903094008	5.762214616
Al	0.748524375	13.075014203	8.461177610
Al	4.849100545	15.173754191	7.101151749
O	3.422185825	15.474126553	11.110519035
O	7.709321877	13.079116718	9.688954509
O	3.364468832	15.011089080	8.330918918
O	7.716673107	13.152417223	7.040683034
Al	4.877389283	11.298121674	9.954431424
Al	0.566585189	9.203674474	11.188118817
O	7.632438208	9.033618635	7.205269266
O	3.578967885	11.218120577	8.540848348
O	7.627766572	9.340231296	10.079025685
O	3.491491039	11.058823200	11.127539764
Al	7.774667233	11.265765429	9.985030203
Al	3.555915404	9.195234550	11.174714045
O	4.910900414	8.954319687	7.359608015
O	0.697186761	11.222021859	8.564107697
O	5.062185138	9.345020313	10.078501750

O	0.699092491	11.036528116	11.204122650
Al	3.525733251	13.057588384	8.335324251
Al	7.701783406	15.231232643	7.172880364
O	0.785947797	15.327455218	11.296411518
O	4.898284127	13.083873116	9.684007067
O	0.711619319	14.998322801	8.326696812
O	4.892189203	13.288882960	7.053795058
Al	6.302041322	9.153495798	8.611952278
Al	6.358273979	9.148303455	11.432908379
Al	6.294933656	14.168671975	9.947565248
O	6.077139599	15.249253457	11.283051049
O	6.316871645	15.256864939	8.467129163
Al	2.175005733	15.170875099	9.818684002
Al	2.080151560	15.081539559	6.960951398
Al	2.150743361	10.201498573	8.476547132
O	1.886999058	9.043434452	7.123924071
O	2.036700774	9.029840495	9.987924923
Al	2.099886933	12.140068088	11.237575347
O	2.099095384	13.292063283	9.824712671
O	2.053900356	13.176262291	7.186338534
Al	6.163774759	12.109332678	7.199238171
O	6.316674550	11.041793707	8.632060065
O	6.308078613	11.209368504	11.293452610
H	0.904024802	9.180190085	7.022145520
H	4.960963148	9.564842767	6.601020115
H	7.244719767	9.529069566	6.443536920
H	6.317450101	14.567136278	5.220332436
H	3.543779047	14.312372284	5.309515882
H	0.644989748	15.747637670	5.049189639
H	5.837707210	11.208677691	4.993124610
H	8.267140902	12.943294937	6.257318126
O	6.315565358	15.254771886	5.899800816
O	3.499794538	15.174889011	5.756068725
O	0.625990161	15.089453988	5.759269649
O	6.236045031	10.973911683	5.842045463
Al	9.141672298	13.065609344	8.493262528
Al	13.268059372	15.180860025	7.076453556
O	11.839643778	15.468835849	11.047148737
O	16.126856335	13.067948130	9.672519680
O	11.819230390	15.013413128	8.331353800
O	16.126994269	13.148243111	6.968629609
Al	13.246648935	11.286877850	9.890036885
Al	9.116996220	9.229077398	11.330133634
O	16.045610540	9.033294360	7.195914332
O	11.986956516	11.223417038	8.484666462
O	15.976775603	9.291803822	10.010798561
O	11.913684127	11.070498779	11.117397688
Al	16.208709891	11.245267843	9.881296247
Al	11.947169572	9.174967394	11.058666350
O	13.323067908	8.951407673	7.287009581
O	9.112034698	11.232442804	8.633881746
O	13.464925863	9.352676916	10.008272868
O	9.118281617	11.059786965	11.280871755
Al	11.921137287	13.069078641	8.330932831
Al	16.127494304	15.237121024	7.095895495
O	9.186430110	15.262817787	11.429477038

O	13.296319952	13.090856144	9.756777417
O	9.169817585	15.007782857	8.399522893
O	13.321620614	13.294234933	7.108225077
Al	14.689162992	9.143225480	8.554988485
Al	14.675710625	9.087554679	11.433023889
Al	14.722847595	14.133924980	9.835180831
O	14.574014427	15.248816792	11.272612542
O	14.735133521	15.266762350	8.430741547
Al	10.576902765	15.171762382	9.778795973
Al	10.500177129	15.095490517	6.982030433
Al	10.535305336	10.204441446	8.456110694
O	10.291944914	9.043427672	7.108796942
O	10.445903041	9.033470963	9.917618987
Al	10.515371093	12.128664694	11.231415445
O	10.520640187	13.296917975	9.833985451
O	10.452710894	13.213318817	7.199162372
Al	14.587866879	12.099513295	7.118578421
O	14.723787776	11.048788064	8.570486012
O	14.728659897	11.134057511	11.200761158
H	9.306284578	9.169226895	7.007131255
H	13.385487571	9.544058437	6.515731483
H	15.678844513	9.507629463	6.413485463
H	14.726576164	14.568250866	5.163901237
H	11.926194938	14.326680658	5.288485943
H	9.005060801	15.752330185	5.119282038
H	14.282023207	11.203883162	4.898446281
H	16.714032920	12.925378219	6.215920948
O	14.720841485	15.253582866	5.845496513
O	11.909303099	15.178177149	5.756195827
O	9.032869143	15.094653921	5.829733905
O	14.646729225	10.973138974	5.763026310
O	7.778497150	9.123298319	12.620713367
H	7.947186478	8.497723249	13.354849616
O	12.000255019	10.568309706	14.241881725
H	14.835038268	11.715754318	11.977026129
O	13.161922332	9.118875427	12.558899942
O	10.803449711	9.195094855	12.571358691
S	11.965853785	9.251127929	13.663992241
O	11.994984671	8.073660623	14.480073109
O	-0.701900038	9.125236479	12.552615828
H	-0.515578062	8.624978794	13.369117751
O	0.856863092	15.251183474	13.776032139
O	-0.626010190	13.715395965	12.558213684
O	1.794049102	13.375657043	12.540079014
S	0.675759539	14.403929630	12.625152751
O	9.115974347	15.087225350	13.901604534
O	7.868802019	13.472304767	12.559691907
O	10.288679194	13.379913221	12.561807372
S	9.104002228	14.320210077	12.682043009
H	15.088284328	14.774147335	11.991477906
H	4.313097808	15.056109666	11.247805665
H	12.612754434	14.920757883	11.257512302
O	0.934902734	7.173788858	13.822818790
O	-0.552037046	5.654371513	12.562977737
O	1.869022561	5.335193874	12.493012945
S	0.746379365	6.337092460	12.662083754

O	9.266909142	7.108683490	13.771731300
O	7.861863606	5.570870067	12.477924970
O	10.294327851	5.316671348	12.479091983
S	9.135956149	6.290493668	12.592343416
H	6.718825205	6.692938628	11.927440463
H	4.242395909	6.852477723	11.246664912
H	12.591090049	6.744299987	11.122147453
H	15.159855733	6.701449578	11.923722396
O	7.645286782	1.125989652	12.634528290
H	7.773001919	0.562663521	13.419596565
O	3.278063370	0.880431429	14.803679296
O	11.987710584	-0.079474486	14.471057729
O	-0.709679103	1.129504453	12.619278350
H	-0.537334337	0.607278273	13.425558157
O	3.425531675	3.137261059	13.771793215
H	6.403963674	3.713930839	11.973947920
O	4.607180796	1.209311035	12.698294692
O	2.253811593	1.290710769	12.558140044
S	3.392538448	1.700038889	13.636788791
O	11.995219963	2.417156036	14.466317789
H	14.836906294	3.730471547	11.969756824
O	13.148367859	1.133109577	12.632653386
O	10.767064601	1.205352692	12.656786440
S	11.949551862	1.166344061	13.758424609
O	4.361569532	11.052408067	13.847092578
O	2.651107811	9.443549198	12.953100610
O	4.916048336	9.026495461	12.630685617
O	3.979941041	8.878617901	14.963397332
S	3.946485709	9.622368284	13.742442408
H	4.613474537	13.199660058	12.596091645
Zr	6.122293996	12.334425532	13.152813922
C	5.441364176	14.142292603	14.657690961
C	6.467275996	13.547642537	15.382149592
H	6.243989766	13.133192155	16.364433675
H	7.493415281	13.894504672	15.252450518
C	6.969705797	11.460374389	15.057014010
H	6.218905412	11.100242199	15.772935031
H	6.990892188	10.643066434	14.263683213
H	5.689657193	14.940062916	13.950336957
C	8.365510094	11.453526015	15.670250289
H	8.403549614	12.187067054	16.491823242
H	9.119169653	11.777780981	14.934306582
C	4.007830224	14.048844900	15.123148423
H	3.305196575	14.279009525	14.320390149
H	3.775772666	13.043946606	15.502150110
H	3.829808422	14.771347143	15.932976394
C	8.749071114	10.071719481	16.221528678
H	7.921718534	9.686575711	16.842290971
H	8.857239256	9.359721482	15.386452089
C	10.045692174	10.074339022	17.034546837
H	9.928082200	10.735407213	17.911192784
H	10.851895355	10.510969972	16.425472629
C	10.459396908	8.671467034	17.489368994
H	10.468891075	8.001044536	16.615142756
H	9.700599428	8.267200479	18.182401178
C	11.837194159	8.636232313	18.152847028

H	12.577884488	9.058685972	17.452450017
H	11.841744832	9.296887899	19.038373488
C	12.286558724	7.232580393	18.564414940
H	12.290539820	6.577886746	17.675932545
H	11.547555154	6.794926923	19.259421141
C	13.670962690	7.211997721	19.217557847
H	14.403818181	7.647538352	18.517843966
H	13.663586359	7.876207997	20.099018682
C	14.125128122	5.811037407	19.630760381
H	14.172190746	5.138030543	18.762040302
H	13.425195129	5.364814622	20.353621952
H	15.121825947	5.826429229	20.094581782

AlS/ZrH-nonyl

Al	0.737183384	4.998741780	8.411894173
Al	4.847082777	7.126261440	7.064493559
O	3.423947843	7.361531601	11.114074011
O	7.711240076	4.997124099	9.605333542
O	3.365122605	6.954657665	8.318733079
O	7.710666071	5.079343888	6.959349607
Al	4.832923560	3.241644644	9.892314220
Al	0.669939277	1.211702729	11.352633418
O	7.636902509	0.961379656	7.262060345
O	3.580250519	3.161620961	8.419383039
O	7.565541217	1.219237157	10.087706882
O	3.438382169	3.063708949	11.039978648
Al	7.776374219	3.157980410	9.906481592
Al	3.485084846	1.169951989	11.084122209
O	4.908905300	0.874947539	7.356868892
O	0.703140537	3.157348672	8.551575198
O	5.016270069	1.317380670	10.057698183
O	0.636036997	3.051749264	11.204524551
Al	3.499126230	4.995928524	8.271155318
Al	7.702046449	7.165590033	7.084950251
O	0.858568490	7.280391699	11.333191857
O	4.842124062	5.067920741	9.687927151
O	0.706003422	6.937641648	8.325747537
O	4.902622260	5.238273142	7.044008350
Al	6.274389536	1.055392919	8.638189921
Al	6.227931615	0.860705492	11.469504788
Al	6.308086540	6.044587914	9.784261470
O	6.233958773	7.171873041	11.204521944
O	6.319129413	7.204724561	8.407729591
Al	2.173596541	7.085097969	9.785555338
Al	2.067790330	7.049622676	6.952632115
Al	2.144766454	2.118746828	8.429043639
O	1.938863504	0.971445859	7.047190212
O	2.013080274	0.975069162	9.905939574
Al	2.047569934	4.090480372	11.182323217
O	2.090656540	5.231986284	9.761844569
O	2.038503482	5.158500830	7.124606414
Al	6.154073382	4.019038487	7.123549795
O	6.308013198	2.977329396	8.579343383
O	6.306386201	3.151603690	11.183890995
H	0.963232467	1.106200981	6.919158109
H	4.960127561	1.475861555	6.592323846

H	7.259762386	1.427142596	6.480674751
H	6.297722478	6.487099944	5.171451010
H	3.512537469	6.359223709	5.234970143
H	0.614306728	7.704568976	5.054097698
H	5.797486774	3.099646870	4.925921994
H	8.255304713	4.865751735	6.172913199
O	6.307060985	7.188415913	5.836872297
O	3.493579472	7.182048552	5.751452310
O	0.622348792	7.031838223	5.751252045
O	6.234931021	2.903806089	5.764854745
Al	9.139359568	4.981037345	8.402878723
Al	13.246221630	7.113788528	7.023593713
O	11.846156314	7.341377628	10.959701803
O	16.118809458	5.003708460	9.606290218
O	11.774035107	6.949729846	8.256282193
O	16.125736759	5.080734801	6.958250783
Al	13.243617021	3.246248083	9.894749528
Al	9.013231150	1.172842180	11.369296687
O	16.045375257	0.963665519	7.208296587
O	11.984306560	3.154371281	8.482032198
O	15.979837433	1.273507450	10.080194494
O	11.909714906	3.062409655	11.121241332
Al	16.204959866	3.174510464	9.878013587
Al	11.928454459	1.173126665	11.149077369
O	13.323732371	0.878124445	7.358908375
O	9.110488990	3.148267546	8.560731039
O	13.459358516	1.300934888	10.074828693
O	9.103722342	2.989000609	11.205263299
Al	11.902988430	4.999140411	8.270897847
Al	16.117607084	7.161752166	7.083143303
O	9.259580237	7.253539047	11.288348991
O	13.257682673	5.067835244	9.691071012
O	9.122598301	6.933622105	8.320158273
O	13.312230325	5.227511136	7.042001745
Al	14.709630519	1.074396520	8.617794960
Al	14.687791826	1.059813958	11.481303490
Al	14.716869628	6.056690344	9.773536607
O	14.643410515	7.169181667	11.204040217
O	14.722235563	7.204088931	8.393505284
Al	10.526078675	7.088085250	9.727015866
Al	10.483347264	7.040281903	6.914722492
Al	10.544930151	2.115853746	8.486719151
O	10.349186388	0.966132221	7.119173656
O	10.427129182	0.962392413	9.985878217
Al	10.484285800	4.073942214	11.178042589
O	10.504770653	5.209936900	9.749003904
O	10.447598190	5.146856805	7.118473631
Al	14.577095255	4.026785772	7.113851366
O	14.718274861	2.983402990	8.573001279
O	14.718947862	3.144683317	11.199345866
H	9.368829957	1.097644737	6.995978523
H	13.366080957	1.478727150	6.593264554
H	15.654413879	1.432366154	6.434789709
H	14.735444247	6.504477656	5.141656700
H	11.915265870	6.359153945	5.162780466
H	9.010751754	7.702506893	5.046687309

H	14.221969319	3.105750594	4.918727558
H	16.684000169	4.867646639	6.180874718
O	14.722572816	7.183929533	5.829317814
O	11.903136818	7.177912722	5.685560959
O	9.030740055	7.031806016	5.745615870
O	14.647066172	2.902903789	5.762432713
Al	0.741333107	13.065676528	8.429877147
Al	4.851810186	15.177843633	7.077337450
O	3.427393807	15.472345646	11.046840996
O	7.706849853	13.074434705	9.687884690
O	3.359616099	15.006602616	8.317282991
O	7.715003866	13.150732759	7.035752761
Al	4.863898102	11.288088133	9.915248471
Al	0.559489368	9.181421254	11.181399184
O	7.632517121	9.033360046	7.204812042
O	3.582267183	11.214923934	8.486615866
O	7.626057274	9.340212027	10.079304084
O	3.495842786	11.073191798	11.122012966
Al	7.786685895	11.259324314	9.972730613
Al	3.554494240	9.195456931	11.178067778
O	4.911338425	8.953197604	7.359831120
O	0.697211639	11.217618890	8.561007359
O	5.061326540	9.347208260	10.077314765
O	0.696642868	11.003015901	11.200342480
Al	3.513800109	13.060270809	8.307428341
Al	7.693159228	15.233590806	7.141791638
O	0.784299905	15.327698259	11.283980840
O	4.895267007	13.079497459	9.686532629
O	0.712525693	14.995803494	8.323713124
O	4.894820116	13.292276126	7.048440001
Al	6.305672061	9.148397121	8.607534364
Al	6.360477151	9.115073237	11.429852142
Al	6.297274521	14.175361690	9.919480798
O	6.074680826	15.240593676	11.262826968
O	6.311921358	15.252003115	8.422060136
Al	2.159656335	15.163983988	9.791624368
Al	2.078953263	15.083597688	6.945930119
Al	2.149512187	10.196965995	8.464387740
O	1.882823077	9.039792002	7.120313142
O	2.041343681	9.029282250	9.984440885
Al	2.071970884	12.124438216	11.199482287
O	2.099466428	13.254592725	9.769448136
O	2.042609750	13.178127777	7.138361666
Al	6.163789052	12.110473813	7.192445929
O	6.316469918	11.041216870	8.631294741
O	6.301653294	11.204065207	11.298079461
H	0.898878496	9.174304951	7.020118593
H	4.954477116	9.566173119	6.603007862
H	7.244059478	9.527161310	6.442247242
H	6.314294668	14.559620771	5.176989123
H	3.537618187	14.322086927	5.288636929
H	0.630626840	15.751287421	5.046519225
H	5.837806698	11.203573412	4.990704656
H	8.268897317	12.932133394	6.257225347
O	6.313634598	15.255767942	5.847712884
O	3.498811981	15.178413699	5.746992338

O	0.622448067	15.092308892	5.756252089
O	6.236024250	10.972707118	5.840744415
Al	9.134988324	13.072000507	8.489655212
Al	13.260860287	15.182647210	7.078403541
O	11.837773996	15.467847442	11.045914010
O	16.129026889	13.064935216	9.669637514
O	11.791170443	15.015952418	8.331734501
O	16.125375634	13.147381638	6.967062248
Al	13.244648211	11.288767371	9.890492985
Al	9.123747719	9.227726868	11.341654644
O	16.045172456	9.032777911	7.195894034
O	11.986256092	11.224982330	8.484296617
O	15.973032706	9.287062446	10.007315967
O	11.912369692	11.071983024	11.117510530
Al	16.205669456	11.239069001	9.877900154
Al	11.946559019	9.175083723	11.060466190
O	13.322694732	8.950945180	7.286858869
O	9.112831598	11.233112639	8.630708189
O	13.463419097	9.353358259	10.008326238
O	9.116655813	11.060333468	11.279586462
Al	11.913013851	13.072582433	8.334173483
Al	16.123948789	15.240580571	7.095433813
O	9.185064269	15.262135668	11.429518948
O	13.292209587	13.093784444	9.756798006
O	9.132906029	15.006750493	8.396971602
O	13.318140025	13.298025478	7.111384034
Al	14.686588888	9.140563460	8.555289516
Al	14.671629991	9.087754589	11.435017162
Al	14.723801261	14.132282905	9.837431029
O	14.574267500	15.248164816	11.273068788
O	14.733138424	15.266298371	8.432477750
Al	10.562179476	15.173478287	9.785907837
Al	10.488826394	15.098904278	6.982954317
Al	10.537081571	10.203274973	8.456459173
O	10.292630360	9.043517232	7.108627588
O	10.445614247	9.034012149	9.917998233
Al	10.514760615	12.131331047	11.233778970
O	10.516891354	13.297467387	9.833173716
O	10.446942545	13.215643539	7.198737728
Al	14.580892277	12.099795203	7.119058361
O	14.723152157	11.048547838	8.569991452
O	14.728692328	11.134042014	11.200101336
H	9.307693452	9.169694871	7.005463665
H	13.386772572	9.542182162	6.514654091
H	15.678477390	9.507243206	6.413574618
H	14.722549587	14.572492035	5.161012416
H	11.922358026	14.326855493	5.291376521
H	9.011499278	15.748162356	5.104379976
H	14.281112638	11.201381076	4.898000639
H	16.703380279	12.932250540	6.204448298
O	14.719144547	15.253839981	5.846538514
O	11.906169653	15.179088839	5.757730176
O	9.033289025	15.095621355	5.819796377
O	14.646168366	10.973829890	5.763347684
O	7.774002610	9.126626327	12.616827975
H	7.941908736	8.541906178	13.382628312

O	12.000580191	10.568209030	14.241060250
H	14.838199274	11.715649384	11.976232272
O	13.160209969	9.118761925	12.558447365
O	10.799177096	9.195633814	12.570875108
S	11.963695413	9.250005612	13.668114583
O	11.993219558	8.072460796	14.481384998
O	-0.703388823	9.122728454	12.551344368
H	-0.522935337	8.629864582	13.373281327
O	0.860089040	15.244874514	13.765065800
O	-0.623502009	13.713267512	12.555664534
O	1.798618468	13.378697989	12.480689519
S	0.679817472	14.402559650	12.612088261
O	9.115163129	15.088513219	13.904382230
O	7.863613546	13.473032390	12.564194646
O	10.286892391	13.381006406	12.560795635
S	9.104285334	14.322102483	12.688569354
H	15.087720692	14.774793570	11.993369039
H	4.329559247	15.077790067	11.190149282
H	12.607506293	14.916594012	11.259069388
O	0.936643957	7.173013399	13.819477494
O	-0.551627164	5.652571377	12.563028060
O	1.870828512	5.330593298	12.492394599
S	0.746998561	6.332268802	12.662700698
O	9.267206231	7.109919167	13.773011250
O	7.860968372	5.572282835	12.477548209
O	10.293157518	5.317019494	12.479103563
S	9.135894883	6.291957422	12.595351958
H	6.731180696	6.685673868	11.921930593
H	4.235605583	6.844204518	11.253080636
H	12.588791758	6.743109201	11.126351221
H	15.156089029	6.701889056	11.925595281
O	7.641690364	1.124635934	12.633023465
H	7.761490137	0.563071322	13.420805134
O	3.353438312	0.480498431	14.651298647
O	11.987807905	-0.079906615	14.471166600
O	-0.709649148	1.128503148	12.617824342
H	-0.541920233	0.609440546	13.426843304
O	3.428409416	2.901496781	14.008017747
H	6.409621093	3.714585083	11.971352786
O	4.620565525	1.204847214	12.627760257
O	2.255366270	1.281297893	12.545331029
S	3.413558596	1.504504647	13.656604928
O	11.995334967	2.417350449	14.466365804
H	14.835239157	3.728593298	11.971206862
O	13.148680165	1.132942593	12.632022276
O	10.767150331	1.205262591	12.657372216
S	11.948613577	1.166162064	13.759778146
O	4.368810928	11.060707652	13.839634776
O	2.653800568	9.446797162	12.950681576
O	4.916891721	9.026612593	12.627587910
O	3.981804737	8.882632028	14.961584340
S	3.948438566	9.626730159	13.742291236
H	5.026089523	13.611294991	13.423971605
Zr	6.197479296	12.236229636	13.112426569
C	7.097268233	11.480497556	14.947959410
H	6.416009202	12.000858849	15.663334486

H	6.842345503	10.407667423	15.036958517
C	8.563113045	11.726667292	15.314481059
H	8.751224280	12.809503215	15.391027205
H	9.222506763	11.361487216	14.510405297
C	8.975346273	11.050136802	16.631603399
H	8.223556103	11.276797281	17.407142638
H	8.946777526	9.955262729	16.490894753
C	10.364251043	11.471055702	17.118404345
H	10.347833836	12.543399371	17.380036150
H	11.084827102	11.368678012	16.293570937
C	10.857371588	10.654750054	18.315987245
H	10.873934373	9.586280407	18.039114836
H	10.138142141	10.743327168	19.149722772
C	12.249479641	11.071556894	18.795750606
H	12.958921490	10.990174548	17.954261563
H	12.235518220	12.139659566	19.075318614
C	12.773953771	10.249327329	19.975329784
H	12.799000349	9.181108963	19.696573093
H	12.067871551	10.324802321	20.821901801
C	14.167068660	10.684478932	20.437501411
H	14.867236484	10.608907707	19.588923169
H	14.138997776	11.753471120	20.708795430
C	14.697526499	9.866428878	21.616188261
H	14.766994404	8.799275446	21.357133906
H	14.031352468	9.951800241	22.488501085
H	15.698598127	10.200169936	21.925018658

Nonyl hydrogenolysis TS

Al	0.733443841	4.993266789	8.410950133
Al	4.846890752	7.120821175	7.062497794
O	3.421357685	7.350990916	11.120821275
O	7.715281567	4.997786510	9.602603541
O	3.363019986	6.951053662	8.320444671
O	7.711227898	5.078844173	6.958972743
Al	4.826585011	3.258871997	9.936778006
Al	0.666011433	1.143574451	11.355314130
O	7.637270403	0.959866797	7.275779634
O	3.570556331	3.158742884	8.480315795
O	7.560893211	1.230045068	10.091305039
O	3.437829449	3.067914348	11.118879671
Al	7.773395277	3.164614431	9.909255003
Al	3.486371812	1.181221093	11.108147741
O	4.908450036	0.869798858	7.366901787
O	0.702766277	3.151383472	8.554407490
O	5.013557549	1.359115653	10.080859732
O	0.641598186	2.989036249	11.208237030
Al	3.499335727	4.989167250	8.276453279
Al	7.704964918	7.161136303	7.084125823
O	0.853949572	7.266470458	11.352349630
O	4.833113169	5.072287331	9.687799020
O	0.706558374	6.935998039	8.327925049
O	4.900850680	5.231636356	7.045523498
Al	6.267275944	1.063053662	8.654936992
Al	6.224845694	0.845294873	11.483684898
Al	6.307711683	6.029195122	9.775615064
O	6.240304712	7.110659156	11.215055220

O	6.316995305	7.192769732	8.403788337
Al	2.174582595	7.076240883	9.785929068
Al	2.067738789	7.048292425	6.954762081
Al	2.142959092	2.106418322	8.438891931
O	1.941577576	0.969848240	7.049324942
O	2.013753785	0.965511420	9.916651462
Al	2.026447864	4.069784690	11.179649575
O	2.081578428	5.223457602	9.766833474
O	2.039898893	5.154644765	7.127287392
Al	6.157908878	4.014238829	7.129582026
O	6.306591038	2.981093063	8.589337529
O	6.309945069	3.217985261	11.188989410
H	0.966430520	1.103319233	6.918656924
H	4.980938602	1.451926993	6.589539281
H	7.258751999	1.410993186	6.487709230
H	6.300835331	6.488128743	5.168127172
H	3.509124579	6.357664159	5.235291203
H	0.613278997	7.706678311	5.058552521
H	5.781072685	3.102482397	4.936271648
H	8.254298232	4.864618631	6.171734853
O	6.306908072	7.186948340	5.836297380
O	3.492619065	7.180626852	5.751774366
O	0.622513167	7.031286270	5.753076037
O	6.234694598	2.903135314	5.765821158
Al	9.142377491	4.980116835	8.405662723
Al	13.252811212	7.115147180	7.063058853
O	11.844427396	7.339988240	11.031469017
O	16.121970543	4.993977402	9.610776541
O	11.775547250	6.945237997	8.311125242
O	16.125014556	5.079742740	6.959661293
Al	13.243414953	3.235820885	9.897473133
Al	9.007795982	1.170632219	11.350755905
O	16.045930457	0.960415738	7.208671028
O	11.986930223	3.150894084	8.481792072
O	15.976893132	1.220913531	10.077750640
O	11.913502448	3.052155226	11.122144896
Al	16.207892626	3.148320188	9.880023374
Al	11.928194149	1.151214345	11.146410280
O	13.324251981	0.865037940	7.358064053
O	9.111289281	3.147319886	8.560824603
O	13.459143182	1.293446546	10.075522820
O	9.104652163	2.988539170	11.206443067
Al	11.902729469	4.993155334	8.282143957
Al	16.117413477	7.165573411	7.085221344
O	9.269877531	7.252209139	11.289912577
O	13.263783119	5.055579904	9.697819580
O	9.123703084	6.932407802	8.324875786
O	13.311651858	5.227674424	7.048303631
Al	14.705526178	1.062258676	8.614578682
Al	14.687208831	1.044360358	11.482394761
Al	14.720014649	6.044195266	9.782348814
O	14.643507027	7.163245797	11.212058372
O	14.725047860	7.194001622	8.403600900
Al	10.548853052	7.077463435	9.761615775
Al	10.479569951	7.044777896	6.948355350
Al	10.545466845	2.113345959	8.485092524

O	10.354142711	0.963497368	7.118598472
O	10.433099427	0.958701186	9.983616694
Al	10.492619602	4.059717752	11.181233582
O	10.506555071	5.190968234	9.754588984
O	10.452632038	5.150310076	7.124118197
Al	14.574889287	4.023352595	7.119569749
O	14.720400916	2.975689995	8.577729635
O	14.724112246	3.134070192	11.198976421
H	9.374340489	1.090817092	6.995481593
H	13.360716846	1.466255847	6.593165179
H	15.653062192	1.425999159	6.434455713
H	14.715534265	6.497455834	5.161210417
H	11.920786089	6.362267690	5.223506961
H	9.021093310	7.709517557	5.059892862
H	14.204734082	3.104639150	4.928026906
H	16.678166032	4.867719654	6.178536147
O	14.718760072	7.187806562	5.837937097
O	11.906242427	7.180542315	5.747125142
O	9.035317125	7.030648207	5.751002812
O	14.646874836	2.903783131	5.763486985
Al	0.755769936	13.060619952	8.453921308
Al	4.854223778	15.160684652	7.118240828
O	3.440812889	15.473230058	11.045274521
O	7.706288558	13.063934072	9.680493893
O	3.364327407	15.004805871	8.332097270
O	7.717875788	13.148793879	7.040543832
Al	4.879825685	11.276680047	9.976434497
Al	0.548147259	9.163340643	11.171527849
O	7.632801996	9.029064863	7.205353207
O	3.580677607	11.207196659	8.562277404
O	7.636416274	9.280751917	10.076909454
O	3.508006352	11.045171592	11.191322189
Al	7.776183638	11.238882618	9.973921245
Al	3.567284574	9.177967800	11.192529364
O	4.909470429	8.950003694	7.357998851
O	0.696200800	11.214770759	8.564537202
O	5.060299072	9.285806619	10.082880954
O	0.705653616	10.981605678	11.203723499
Al	3.533128128	13.047021379	8.368183294
Al	7.700710790	15.232266763	7.182101799
O	0.781784001	15.249330618	11.287961161
O	4.892451778	13.069899907	9.746593501
O	0.718302465	14.988299148	8.327341224
O	4.901118304	13.260593783	7.112392884
Al	6.299799458	9.125081539	8.608209591
Al	6.379028389	9.069044624	11.413530520
Al	6.298494535	14.154936787	9.964490962
O	6.077018681	15.249435676	11.272158614
O	6.315483850	15.253302820	8.473826091
Al	2.173120542	15.149324841	9.812361275
Al	2.087655328	15.069087710	6.960913512
Al	2.150581222	10.193777664	8.476998009
O	1.884158222	9.041697917	7.123913447
O	2.040808767	9.018761803	9.995568732
Al	2.092305462	12.100596791	11.240751289
O	2.101916070	13.246248009	9.824327383

O	2.078122971	13.164844327	7.191201602
Al	6.178810068	12.089803590	7.214889139
O	6.316658913	11.000198065	8.638350724
O	6.317163756	11.137288184	11.286557158
H	0.899443444	9.177263424	7.025351204
H	4.971625102	9.557857330	6.597812410
H	7.245176231	9.520680939	6.441694729
H	6.324119218	14.569675440	5.220989971
H	3.546445182	14.310054841	5.305083451
H	0.650611996	15.746796380	5.052421003
H	5.839433680	11.221833374	4.997957736
H	8.265911661	12.942157255	6.254789346
O	6.313097757	15.249237531	5.908414056
O	3.505171843	15.168952133	5.758871492
O	0.627595194	15.087113092	5.761001681
O	6.236773387	10.972575582	5.843070491
Al	9.141582897	13.047909183	8.491964001
Al	13.267545687	15.158503635	7.078719260
O	11.836452351	15.427290182	11.047178009
O	16.132622560	13.061790134	9.665194624
O	11.816775134	15.000039924	8.329785273
O	16.127379436	13.142602817	6.968126072
Al	13.244675506	11.287911839	9.944353086
Al	9.122818179	9.218211353	11.333975351
O	16.043784449	9.030085554	7.205423737
O	11.984904961	11.214809013	8.552080214
O	15.971266432	9.281216789	10.015261485
O	11.920051660	11.077662620	11.187032218
Al	16.209457412	11.238753340	9.892277961
Al	11.946574970	9.178335952	11.125473924
O	13.318479749	8.950015089	7.355432746
O	9.109913165	11.216580482	8.635643446
O	13.462423337	9.351243619	10.072286068
O	9.115249502	11.050536896	11.281676968
Al	11.920909619	13.049514909	8.344306479
Al	16.129101313	15.234793371	7.096386998
O	9.189320762	15.261320798	11.364445429
O	13.303034577	13.089063722	9.763987232
O	9.176152634	14.998421114	8.397328658
O	13.318827976	13.248921780	7.110302748
Al	14.693439825	9.134926537	8.593721801
Al	14.667011787	9.090956128	11.471375514
Al	14.731886604	14.126425842	9.835701936
O	14.571809178	15.239477161	11.273491141
O	14.733259088	15.254079186	8.423939221
Al	10.563055479	15.154105999	9.785471114
Al	10.500693392	15.074057461	6.984017670
Al	10.541478902	10.192284934	8.476497775
O	10.295093285	9.043925023	7.116836210
O	10.461296355	9.031561100	9.940554552
Al	10.514553937	12.123785339	11.247063503
O	10.520264091	13.263713191	9.830860697
O	10.454916150	13.168701138	7.200300519
Al	14.598740345	12.074584509	7.157155306
O	14.723278818	11.043055264	8.625622541
O	14.739555939	11.136175680	11.224026465

H	9.309692921	9.172694367	7.015667596
H	13.380147157	9.547413067	6.586688315
H	15.661713136	9.514599466	6.433682922
H	14.732053337	14.566895330	5.160908121
H	11.926852950	14.327353622	5.273810605
H	9.015598737	15.754559094	5.127702439
H	14.316646643	11.128075642	4.954742017
H	16.705956499	12.919395214	6.209222091
O	14.720170921	15.248838235	5.846032342
O	11.910398850	15.170016440	5.757481877
O	9.033198183	15.091055599	5.833010194
O	14.645994866	10.901487991	5.834987426
O	7.782633790	9.122087514	12.632778448
H	7.960411367	8.504558982	13.372426922
O	12.064943011	10.566099307	14.320561117
H	14.875266510	11.719284623	11.995873088
O	13.173041960	9.113844268	12.623233260
O	10.815228600	9.201957152	12.643741297
S	11.985164555	9.252684430	13.738611313
O	11.995415248	8.075251319	14.556375222
O	-0.698301044	9.119208085	12.559604518
H	-0.517800770	8.608773695	13.371525894
O	0.703445593	15.161152199	13.765374508
O	-0.630731469	13.563650403	12.475673839
O	1.793576604	13.316908767	12.555136400
S	0.635629671	14.308189693	12.604205166
O	9.036488119	15.085143259	13.834454098
O	7.861255601	13.457096629	12.416290202
O	10.280065369	13.386604954	12.559024836
S	9.072995597	14.312795611	12.621330669
H	15.043757092	14.748226873	12.001252948
H	4.332678418	15.061664239	11.202070963
H	12.617056673	14.880578314	11.228428082
O	0.868110953	7.111745057	13.837531061
O	-0.558107312	5.581003415	12.550595184
O	1.871094805	5.319284586	12.491555866
S	0.721596810	6.292018718	12.661719821
O	9.341562662	7.108284612	13.769665166
O	7.922851095	5.565243278	12.482893228
O	10.357913418	5.318639574	12.477095209
S	9.193235987	6.286185631	12.598993673
H	6.767549841	6.629458171	11.914729445
H	4.231019408	6.827412602	11.254345221
H	12.611581614	6.766736980	11.176175091
H	15.128637829	6.683736306	11.940044352
O	7.647606126	1.122638829	12.624471112
H	7.770432397	0.558390552	13.410331903
O	3.437211125	-0.312459321	14.243789888
O	11.987301664	-0.080285141	14.472013765
O	-0.710186673	1.061649108	12.615705542
H	-0.540215374	0.496101663	13.395293231
O	3.348895519	2.172709046	14.552527859
H	6.443061606	3.758500080	11.986937719
O	4.629791506	1.200906043	12.642074963
O	2.258015991	1.141066779	12.560647391
S	3.414472526	1.014400303	13.702028045

O	11.994994860	2.416203371	14.464300633
H	14.851993435	3.716856504	11.969892868
O	13.149344702	1.129796185	12.630915780
O	10.769737243	1.200716110	12.656633054
S	11.948149676	1.164432649	13.757433742
O	4.587544165	10.966039746	13.921270326
O	2.748150774	9.446959540	13.046864493
O	4.977985227	8.948863791	12.638224116
O	4.133854387	8.801330974	15.002717814
S	4.071258103	9.547254264	13.783896755
H	4.956315589	13.275015997	12.622186616
Zr	6.251432618	12.163993068	13.181391128
C	7.567687413	12.144577979	15.202893005
H	7.864632428	12.861734215	15.984200865
H	8.487671002	11.960894126	14.618874219
H	6.642665469	13.117620564	14.815439000
H	5.855023810	13.663974709	14.276258380
C	7.033508460	10.854772694	15.829763801
H	6.053376233	11.036061521	16.298741734
H	6.848194755	10.098033932	15.047353815
C	7.980969501	10.231136495	16.866257956
H	8.133526312	10.959319687	17.679209713
H	8.970305380	10.067785326	16.406051476
C	7.449088952	8.906327659	17.433048342
H	6.438801943	9.063003947	17.848906434
H	8.087986855	8.596526708	18.276459745
C	7.415223199	7.779096811	16.398848683
H	6.762148283	8.066423691	15.556735499
H	8.426956667	7.648752791	15.984297404
C	6.919530142	6.431842017	16.928640566
H	5.897628882	6.542297386	17.330092110
H	7.554775709	6.113641841	17.773638131
C	6.939119219	5.353975556	15.842530873
H	7.952921110	5.302593801	15.412742753
H	6.278535537	5.666200320	15.013521430
C	6.530528039	3.956229872	16.308880404
H	7.175373967	3.654055195	17.151565838
H	5.498844696	3.975852404	16.696587663
C	6.637900520	2.926231581	15.184254103
H	7.655350399	2.900291201	14.767600196
H	5.943080510	3.167427478	14.366442759
H	6.379245423	1.914704468	15.529040861

σ-bond metathesis TS

Al	0.737923974	4.993841931	8.413931260
Al	4.846523511	7.121982538	7.064112714
O	3.423594317	7.348970734	11.120405415
O	7.713552852	4.994034827	9.607566406
O	3.365108892	6.950612520	8.321056927
O	7.711680405	5.078957209	6.960076015
Al	4.845852189	3.237294994	9.916641637
Al	0.690774730	1.167640832	11.367604306
O	7.637728045	0.956933467	7.268097064
O	3.574294363	3.153679045	8.478891335
O	7.579987328	1.220395061	10.088044977
O	3.497734973	3.057535524	11.115414890

Al	7.781966382	3.159390769	9.906817474
Al	3.522573698	1.137294256	11.098684266
O	4.910405147	0.865360624	7.360768900
O	0.703838633	3.150726879	8.553183754
O	5.046735920	1.303925170	10.071712327
O	0.692457742	2.994018819	11.200991470
Al	3.502286131	4.989120778	8.282010745
Al	7.703212788	7.162717977	7.085255865
O	0.854295660	7.265169933	11.351530293
O	4.842437794	5.060231051	9.689081414
O	0.707401715	6.934884652	8.328684190
O	4.900495672	5.231400662	7.045744728
Al	6.277572337	1.053030951	8.642879595
Al	6.265591234	0.847834116	11.479178490
Al	6.307688864	6.033285418	9.781692770
O	6.236510284	7.115156530	11.213701231
O	6.317163020	7.191000011	8.404110255
Al	2.179718097	7.077791815	9.791175860
Al	2.068786064	7.048035101	6.956740545
Al	2.144137213	2.106227013	8.441301173
O	1.940762257	0.969983491	7.050585048
O	2.027409935	0.962256341	9.919678571
Al	2.083964321	4.068691460	11.188344160
O	2.092590233	5.219902479	9.767378887
O	2.042332709	5.153546279	7.128786817
Al	6.158584105	4.017623223	7.126817247
O	6.308617076	2.975162439	8.584126989
O	6.315138806	3.150544291	11.191121032
H	0.965746446	1.105954018	6.920382418
H	4.972524948	1.456519766	6.589204668
H	7.261856734	1.418023351	6.483633180
H	6.299876242	6.489670592	5.166833519
H	3.508958578	6.355698037	5.238252999
H	0.615562378	7.706705232	5.059345483
H	5.790453430	3.101827232	4.931901296
H	8.258320662	4.865577390	6.175077527
O	6.306761553	7.186920525	5.836508532
O	3.492655239	7.180406758	5.752013357
O	0.622993917	7.030990435	5.753578193
O	6.235251591	2.902962961	5.766271639
Al	9.142377807	4.982195294	8.410649573
Al	13.253270493	7.115988523	7.064551698
O	11.845329763	7.337146956	11.034879688
O	16.124227439	4.993876651	9.611704961
O	11.773769280	6.945672398	8.314414625
O	16.125366010	5.079935432	6.959977540
Al	13.245501806	3.240529695	9.897848660
Al	9.037770195	1.181421442	11.343934878
O	16.046100751	0.960412739	7.208751198
O	11.987971194	3.152885283	8.482576681
O	15.987048152	1.227467474	10.083657804
O	11.911643534	3.058082751	11.123607662
Al	16.223426739	3.153654133	9.889263845
Al	11.936098576	1.154402345	11.145076235
O	13.324745407	0.866540482	7.358662396
O	9.111598410	3.147431744	8.559687070

O	13.462494944	1.295381960	10.076344759
O	9.108891412	2.993542461	11.204945433
Al	11.904490040	4.996788002	8.284546361
Al	16.118603717	7.165003575	7.085552854
O	9.266378818	7.245319438	11.353857936
O	13.264834990	5.059414891	9.698779865
O	9.122873154	6.931993174	8.329554977
O	13.311916591	5.227916311	7.048975122
Al	14.709211963	1.067129145	8.614393556
Al	14.695926211	1.048820319	11.479641629
Al	14.723962303	6.045383199	9.783821433
O	14.643793236	7.162141442	11.213876044
O	14.725648461	7.194179514	8.404446075
Al	10.542419630	7.075529428	9.777138925
Al	10.479723938	7.046280986	6.952952051
Al	10.548000542	2.117343282	8.482989652
O	10.354963561	0.963819919	7.119196811
O	10.437331627	0.958775091	9.983082784
Al	10.490328196	4.077824402	11.209330990
O	10.504359545	5.196477381	9.766492091
O	10.452260064	5.150066833	7.127927530
Al	14.576668203	4.023958819	7.121143120
O	14.721458983	2.976632737	8.579928802
O	14.727912623	3.137486676	11.199491157
H	9.375316116	1.089765709	6.995871184
H	13.361596201	1.467872227	6.593711785
H	15.653292238	1.427502408	6.435164575
H	14.715468834	6.496707429	5.162638317
H	11.920102345	6.361831478	5.226330226
H	9.021941832	7.710990294	5.064232687
H	14.210305194	3.107602121	4.926871916
H	16.680367765	4.867242869	6.180382684
O	14.718848548	7.187956300	5.838456242
O	11.905885117	7.180709812	5.749023041
O	9.035716905	7.030048890	5.753371483
O	14.647023939	2.903728671	5.764410461
Al	0.748481440	13.060854705	8.463664858
Al	4.855078208	15.160093546	7.089711209
O	3.444837720	15.426393254	11.050220486
O	7.708271817	13.065346020	9.683767218
O	3.365110330	15.001357756	8.324104015
O	7.715403410	13.149369294	7.039800921
Al	4.875045445	11.270984610	9.964155436
Al	0.556668925	9.164969568	11.182291768
O	7.633073950	9.028698862	7.206628725
O	3.577220203	11.208238957	8.555817774
O	7.634248527	9.282484090	10.079684562
O	3.508432675	11.049329483	11.183865750
Al	7.773844024	11.236229816	9.979530688
Al	3.569522191	9.176876431	11.211796994
O	4.910228137	8.949849258	7.358924898
O	0.693401297	11.215173514	8.567474166
O	5.058830634	9.288825954	10.082032047
O	0.705365463	10.986072952	11.206533795
Al	3.527094938	13.043778314	8.337242162
Al	7.699044318	15.227867447	7.156980217

O	0.848618309	15.319492363	11.357262879
O	4.903393893	13.069684254	9.688939247
O	0.715852956	14.991749095	8.333280777
O	4.891591690	13.257835099	7.054385764
Al	6.299301800	9.122652625	8.617202221
Al	6.370690878	9.095322196	11.432128754
Al	6.302036475	14.160941174	9.935333008
O	6.087444591	15.232239270	11.275155611
O	6.318816716	15.249920375	8.450193065
Al	2.176083274	15.144302583	9.809280128
Al	2.082502309	15.072647566	6.960253147
Al	2.147276773	10.189948088	8.483737693
O	1.884497107	9.041787297	7.124691921
O	2.040294992	9.019417321	9.997000925
Al	2.094524507	12.103300398	11.245513615
O	2.098106050	13.248605848	9.824971351
O	2.049689496	13.166782276	7.187226786
Al	6.170390178	12.088395011	7.211347955
O	6.317264831	11.000576744	8.636030059
O	6.311131789	11.146226132	11.289077491
H	0.900357102	9.177529971	7.024498117
H	4.977195376	9.556265391	6.598142099
H	7.242888424	9.520273685	6.444385331
H	6.320738370	14.545078411	5.199192107
H	3.540697728	14.317901341	5.290578182
H	0.645255972	15.752388500	5.056557133
H	5.823396234	11.215719279	5.003110052
H	8.262209844	12.930745686	6.256250838
O	6.314496523	15.252133644	5.858975952
O	3.499995640	15.171157807	5.754583220
O	0.626091362	15.088209452	5.761117009
O	6.237824971	10.971220858	5.841453184
Al	9.141447070	13.047520839	8.491545809
Al	13.268868189	15.159645229	7.078697726
O	11.837562959	15.428971045	11.046803451
O	16.129621153	13.064782677	9.676473197
O	11.819030402	15.000127966	8.329064766
O	16.125287179	13.143268739	6.971227973
Al	13.246204973	11.285584619	9.949709080
Al	9.114077096	9.214549941	11.350462573
O	16.043614599	9.030001731	7.206628730
O	11.983265499	11.212382045	8.554413924
O	15.971433586	9.283988272	10.023598776
O	11.920255676	11.073425031	11.188254324
Al	16.209865822	11.237597926	9.902945615
Al	11.947103287	9.175017549	11.133252673
O	13.319183488	8.950384288	7.357031195
O	9.110032286	11.216851523	8.636716006
O	13.463392794	9.351757835	10.075207294
O	9.115189935	11.051067049	11.281445221
Al	11.921552685	13.045288802	8.342314594
Al	16.127740935	15.233885918	7.097556642
O	9.191008694	15.252623715	11.366242961
O	13.301826252	13.087627162	9.765285464
O	9.175867503	14.998471004	8.394691275
O	13.319124817	13.248132528	7.110917759

Al	14.695625549	9.134424939	8.597639438
Al	14.669373453	9.090772352	11.477579116
Al	14.726051608	14.130657611	9.839286084
O	14.578547924	15.245256473	11.274799588
O	14.733032356	15.255194203	8.424909046
Al	10.570723181	15.153253971	9.776995128
Al	10.501332341	15.072967469	6.983082236
Al	10.538229087	10.186599611	8.486722103
O	10.294456887	9.043738798	7.120404152
O	10.452973649	9.026692655	9.964518374
Al	10.514641692	12.115086136	11.236590019
O	10.521155900	13.265277623	9.829687423
O	10.455407968	13.168804269	7.199834735
Al	14.599420497	12.073283909	7.159482300
O	14.723418743	11.044857602	8.632043806
O	14.736518366	11.134400934	11.255159854
H	9.308643531	9.173590195	7.018609829
H	13.380368985	9.547602944	6.588176956
H	15.660932768	9.513580467	6.434795031
H	14.733447899	14.563747989	5.163976863
H	11.929023309	14.327569383	5.273598516
H	9.018058995	15.746300951	5.116125244
H	14.316059692	11.129854938	4.956870410
H	16.716719030	12.913671170	6.223855118
O	14.720063972	15.248784568	5.846047525
O	11.910790220	15.170020908	5.757601667
O	9.035727954	15.090932496	5.828925587
O	14.645986877	10.901350221	5.836393545
O	7.783481230	9.116890042	12.626165239
H	7.947260457	8.522688353	13.385454078
O	12.066189889	10.566636506	14.321671785
H	14.858317304	11.712580394	12.031868369
O	13.172854915	9.113245507	12.625493556
O	10.815646713	9.201265099	12.649304065
S	11.984287391	9.252054002	13.741952877
O	11.995664843	8.074161487	14.558038214
O	-0.696409210	9.118013967	12.563446876
H	-0.510232414	8.602882515	13.371633648
O	0.859296485	15.174497590	13.843251226
O	-0.624656001	13.711260844	12.556097240
O	1.790126118	13.316046736	12.556109861
S	0.693148548	14.366032627	12.664892471
O	9.109227676	15.090079467	13.839650458
O	7.873642146	13.471420483	12.482850217
O	10.291468057	13.377113679	12.553343223
S	9.105541321	14.317395786	12.624169938
H	15.100412400	14.770609122	11.990043175
H	4.338263727	15.021767062	11.205590871
H	12.612885889	14.878799556	11.240837047
O	0.866845299	7.109390453	13.836750173
O	-0.556529787	5.578659014	12.549818329
O	1.874282462	5.319080547	12.491600724
S	0.724616480	6.289161596	12.660617560
O	9.270025957	7.105131250	13.842152065
O	7.863073904	5.569732134	12.552766589
O	10.295748549	5.304705742	12.541018069

S	9.140554337	6.286632001	12.663014134
H	6.736562349	6.634125325	11.932274300
H	4.241551487	6.838313346	11.256274653
H	12.618021772	6.767715449	11.166659097
H	15.132057714	6.682061676	11.940330078
O	7.702663901	1.120725668	12.629742909
H	7.847139321	0.548731328	13.406298956
O	3.427879096	0.311381312	14.639322134
O	11.988330333	-0.079797883	14.471524472
O	-0.703560180	1.124027597	12.626282342
H	-0.559737635	0.616315157	13.446320735
O	3.504169693	2.745827548	14.154349659
H	6.421854523	3.719933669	11.973641420
O	4.678163667	1.131210622	12.640217670
O	2.322922258	1.212018916	12.569456786
S	3.477094328	1.383272879	13.691181477
O	11.995761917	2.416236046	14.463583369
H	14.849689936	3.718713830	11.972874249
O	13.153371681	1.130177154	12.632129246
O	10.774611353	1.200709304	12.653045789
S	11.952709635	1.164927958	13.756346550
O	4.445499369	10.989897713	13.919321265
O	2.721142885	9.431959011	13.028557776
O	4.964548414	8.961227135	12.635287090
O	4.127188493	8.790866792	14.982404948
S	4.037461373	9.564110645	13.783808069
H	4.652142184	13.245239423	12.883674091
Zr	6.185085152	12.268228620	13.143272761
C	6.985101364	11.645431774	15.046760847
H	6.344980605	11.200762810	15.817888283
C	6.521813093	13.360772968	15.351971835
H	7.383150592	13.747336087	14.793875409
H	6.707699014	12.953501394	16.345222478
C	6.074069156	14.894827958	15.937647887
H	5.866663932	15.521511811	15.057563662
H	6.940974376	10.702681398	14.284703811
H	5.518906804	13.325530087	14.810255754
C	8.477570180	11.576358682	15.406413116
H	9.117283748	11.778188375	14.536167040
H	8.739684292	12.312913236	16.180731321
H	8.749967119	10.582319353	15.783835901
H	6.955401055	15.296808208	16.463229520
C	4.856419252	14.831644892	16.848206833
H	4.543571134	15.859664456	17.093899867
H	4.006496243	14.404427074	16.288731693
C	5.070060705	14.047591018	18.147697221
H	5.951714786	14.448405607	18.679243420
H	5.301962553	12.989663182	17.926674419
C	3.853878161	14.089398051	19.077055652
H	2.974019722	13.695615209	18.540126082
H	3.616919684	15.141387872	19.312322505
C	4.049728599	13.307458674	20.377873460
H	4.278284592	12.253354395	20.140584909
H	4.936822742	13.696697180	20.908720920
C	2.838195267	13.361535665	21.312927537
H	1.954360752	12.970359220	20.782207575

H	2.608618656	14.414828386	21.546435935
C	3.046210932	12.579135834	22.611538821
H	3.905879288	12.970436149	23.176718259
H	3.244546832	11.516522326	22.405944993
C	1.809898097	12.654499896	23.526662218
H	1.588637103	13.703581484	23.756843797
H	0.935593855	12.262589019	22.993267315
C	1.989835212	11.875980883	24.841963744
H	2.838036615	12.264851588	25.414532167
H	2.181402304	10.815935156	24.646756804
H	1.094365138	11.950089058	25.467953904

AlS/ZrH-propyl

Al	0.736756177	4.991884657	8.413126257
Al	4.846214279	7.121006888	7.062951686
O	3.420054231	7.354030036	11.121671103
O	7.716250150	4.998821273	9.604563744
O	3.363879145	6.952258769	8.320158014
O	7.712039593	5.079780086	6.958861020
Al	4.851038335	3.257386973	9.922361394
Al	0.697237023	1.165856004	11.375578929
O	7.638403598	0.959575361	7.274302317
O	3.573559644	3.157246060	8.479860080
O	7.576127468	1.230953558	10.094118836
O	3.496102070	3.068488951	11.118757083
Al	7.781447772	3.166483614	9.911728994
Al	3.526987081	1.172718625	11.103451934
O	4.909508804	0.869633729	7.366938541
O	0.704245640	3.150480687	8.553080735
O	5.054322980	1.356284846	10.077405028
O	0.692302036	2.993234735	11.201849014
Al	3.500923444	4.989005697	8.278558895
Al	7.704798075	7.160952473	7.084143603
O	0.854003415	7.265988301	11.351255861
O	4.836150328	5.072780495	9.685322483
O	0.707575234	6.935381022	8.328854278
O	4.900854762	5.232512997	7.044752048
Al	6.276470501	1.064804989	8.650976154
Al	6.264281033	0.847781359	11.488199904
Al	6.308229073	6.030497287	9.775617042
O	6.238635775	7.109203518	11.213527281
O	6.317368388	7.193049867	8.403485301
Al	2.176451402	7.077192640	9.787248343
Al	2.068122601	7.047329452	6.955295907
Al	2.145797494	2.106688891	8.440992398
O	1.941813975	0.969807853	7.050774086
O	2.027729967	0.966175219	9.920832186
Al	2.078771292	4.071000369	11.186895179
O	2.090924184	5.222277619	9.767368633
O	2.041125997	5.154317862	7.127980694
Al	6.159379839	4.017899550	7.122960621
O	6.310926943	2.982456139	8.579278793
O	6.319418329	3.218566500	11.186051460
H	0.966537560	1.104474047	6.921109337
H	4.975527099	1.454523460	6.591114205
H	7.259709264	1.413610704	6.487561290

H	6.300268750	6.491568862	5.165375011
H	3.509204401	6.358114547	5.234865714
H	0.613752030	7.705670349	5.058156974
H	5.791474143	3.100518018	4.928821401
H	8.259477860	4.866457854	6.174347913
O	6.306881559	7.187474091	5.836488049
O	3.492453283	7.180873353	5.751666593
O	0.622743464	7.031129452	5.753502846
O	6.234952707	2.902726424	5.764139865
Al	9.143459373	4.982195957	8.410393318
Al	13.252577610	7.115141191	7.063759510
O	11.845670541	7.337541569	11.035183685
O	16.124997557	4.991915799	9.611150552
O	11.774083158	6.946350030	8.314059961
O	16.125200808	5.079898532	6.959938855
Al	13.244165772	3.236873228	9.896304904
Al	9.037716324	1.179864491	11.346077403
O	16.046228957	0.960919297	7.208504591
O	11.986820707	3.152122828	8.481806548
O	15.986902528	1.227034375	10.084109383
O	11.912246671	3.058701686	11.123167059
Al	16.223659546	3.149776399	9.889300651
Al	11.936713238	1.153909628	11.146536595
O	13.324728973	0.866079023	7.358828141
O	9.111882591	3.148414195	8.560398052
O	13.462367923	1.295179285	10.075831026
O	9.110445428	2.994628029	11.205657038
Al	11.904161862	4.995504042	8.284612694
Al	16.117712278	7.164078596	7.085228761
O	9.265309542	7.248732045	11.356008581
O	13.261136093	5.054153376	9.694756623
O	9.122863463	6.933282352	8.328362669
O	13.311950241	5.227949680	7.048376261
Al	14.709679074	1.066083652	8.614972171
Al	14.695919579	1.049799204	11.480404872
Al	14.720402161	6.035583782	9.777307173
O	14.641313124	7.126702656	11.219252542
O	14.725232742	7.192930412	8.404219705
Al	10.543177840	7.077741670	9.777442472
Al	10.480209788	7.045749164	6.953048727
Al	10.546742894	2.116236184	8.485148649
O	10.354669077	0.963756481	7.119279385
O	10.437838668	0.959141999	9.982909870
Al	10.491223920	4.078050928	11.208435808
O	10.503788562	5.202634634	9.767569802
O	10.452563545	5.150535185	7.127743854
Al	14.575499864	4.022565825	7.120485476
O	14.721493919	2.976087357	8.579119107
O	14.727601739	3.137692638	11.198867889
H	9.375450284	1.091935220	6.995107778
H	13.359841611	1.467392653	6.593920905
H	15.653231695	1.427863305	6.434981695
H	14.714889816	6.497055892	5.161958522
H	11.919669808	6.362708580	5.224553941
H	9.023843991	7.709141877	5.061358658
H	14.210991942	3.106517847	4.925694891

H	16.679055150	4.867023154	6.179578320
O	14.718757500	7.187941580	5.838170425
O	11.905595678	7.180722895	5.748573177
O	9.036112026	7.030516965	5.752769659
O	14.647017147	2.903636877	5.763822964
Al	0.754296013	13.067955730	8.459014603
Al	4.853818383	15.162756719	7.114267394
O	3.490838581	15.473930157	11.043898039
O	7.708483863	13.062283345	9.687632546
O	3.367774670	15.005026262	8.333103910
O	7.718202347	13.148436298	7.041318733
Al	4.875891419	11.268728476	9.962751661
Al	0.549001636	9.163087136	11.174469351
O	7.632966950	9.029329607	7.205852289
O	3.579479956	11.208999537	8.560523958
O	7.635255343	9.280959465	10.079374869
O	3.510937405	11.055916647	11.187401354
Al	7.782636118	11.230324012	9.971550838
Al	3.567143086	9.182973655	11.202664332
O	4.909871107	8.950601788	7.358352544
O	0.695594916	11.217715084	8.566746394
O	5.059971680	9.288440372	10.081784968
O	0.703010769	10.982975895	11.204263476
Al	3.530986442	13.049703966	8.367695291
Al	7.703031331	15.232004201	7.177522276
O	0.854513401	15.318583280	11.357061698
O	4.897517550	13.069469540	9.755487145
O	0.716421563	14.990466302	8.328776090
O	4.901872518	13.261240277	7.115249975
Al	6.301142448	9.123157283	8.610618774
Al	6.376333656	9.055686501	11.418102351
Al	6.305652458	14.160553109	9.948934061
O	6.084289959	15.243238871	11.270095558
O	6.318263263	15.254190660	8.469626561
Al	2.201282182	15.157922858	9.828826584
Al	2.085044586	15.072436167	6.965041204
Al	2.148075844	10.193672515	8.478144525
O	1.883984557	9.042246128	7.124319044
O	2.041053361	9.022362858	9.997029314
Al	2.084708856	12.102488704	11.240752728
O	2.101596488	13.257074093	9.829045420
O	2.076944775	13.166345173	7.193383220
Al	6.179360065	12.089540287	7.211497134
O	6.317240802	11.003166058	8.639126173
O	6.313546030	11.140743677	11.290984445
H	0.899252483	9.176836366	7.024719341
H	4.972947702	9.556697714	6.596973115
H	7.244763419	9.519384704	6.441750038
H	6.324546990	14.575329457	5.213724055
H	3.541260758	14.310535555	5.304899377
H	0.650681223	15.746617176	5.052448465
H	5.836003303	11.217162110	4.997241838
H	8.270378339	12.938518607	6.259081035
O	6.313424423	15.248964596	5.906930551
O	3.504363020	15.169051498	5.759693059
O	0.627705964	15.087153610	5.761265436

O	6.236583733	10.972336797	5.842139715
Al	9.144428472	13.047554686	8.493850286
Al	13.268608048	15.159738891	7.079292842
O	11.839208157	15.428089230	11.047477093
O	16.131735842	13.065421981	9.674350533
O	11.816745351	14.999611050	8.330287280
O	16.127011501	13.142418162	6.970084952
Al	13.245028103	11.286172230	9.947052822
Al	9.123110211	9.217671812	11.365868130
O	16.043791814	9.029641160	7.205974418
O	11.983765741	11.212231610	8.553967663
O	15.969993763	9.278710072	10.017823519
O	11.919542810	11.075534005	11.188172696
Al	16.206485843	11.239473703	9.895109254
Al	11.947836895	9.176584503	11.134532867
O	13.318681242	8.950007176	7.355852482
O	9.110560544	11.215502971	8.635497982
O	13.461987629	9.346631657	10.073503941
O	9.113170721	11.053058957	11.282675840
Al	11.921926793	13.047164918	8.344055457
Al	16.129111030	15.235588587	7.096697577
O	9.191820353	15.257261653	11.364203663
O	13.302121918	13.088583262	9.764086587
O	9.175909650	14.997796730	8.397662481
O	13.318784258	13.248343756	7.110692307
Al	14.693511069	9.133700574	8.594329571
Al	14.666369681	9.068531281	11.471408758
Al	14.727442371	14.131090266	9.838217925
O	14.578974367	15.244996806	11.274187999
O	14.733029817	15.255024744	8.423665421
Al	10.566623562	15.151787568	9.786143553
Al	10.502035085	15.074126865	6.984370688
Al	10.540364305	10.185650590	8.489066700
O	10.295816435	9.044226537	7.121161626
O	10.452316982	9.028636093	9.970600151
Al	10.513876124	12.121558383	11.248810252
O	10.520779610	13.260189241	9.831085838
O	10.454947194	13.168670743	7.200545898
Al	14.598938042	12.074082543	7.157324828
O	14.723404849	11.043209997	8.627108912
O	14.737923955	11.136450394	11.223919355
H	9.310814017	9.174601452	7.018071704
H	13.380087213	9.547016915	6.586760896
H	15.661416740	9.513484992	6.434024606
H	14.732707744	14.566968175	5.160926611
H	11.928422078	14.327918978	5.273487064
H	9.018582349	15.752839535	5.125426737
H	14.317994417	11.128973849	4.954679325
H	16.708795491	12.917136895	6.214237810
O	14.720205096	15.248823398	5.846132208
O	11.910684721	15.170154033	5.757838269
O	9.033989388	15.091052705	5.832434924
O	14.645978708	10.901362802	5.835206349
O	7.774208386	9.122448480	12.630716953
H	7.942005121	8.544511214	13.402077332
O	12.067043344	10.566838013	14.320866022

H	14.853578440	11.712652704	12.003401507
O	13.173535760	9.113107067	12.622942919
O	10.814241345	9.201235831	12.648377074
S	11.984550625	9.252316259	13.744406052
O	11.995872195	8.073087905	14.557366659
O	-0.699339763	9.117205810	12.559654428
H	-0.515410796	8.605017225	13.370236262
O	0.859325066	15.174159677	13.843710909
O	-0.622234008	13.710560994	12.555241697
O	1.793357750	13.314414191	12.555022772
S	0.694690369	14.365798171	12.666646856
O	9.185371003	15.162693444	13.839679259
O	7.863523964	13.542623663	12.562474104
O	10.285986236	13.382643718	12.557679656
S	9.122720835	14.351794669	12.654906804
H	15.098325618	14.771626631	11.991136715
H	4.400570578	15.090066518	11.198910094
H	12.616583515	14.880518735	11.240488399
O	0.869394501	7.112373954	13.836036007
O	-0.553302922	5.576884879	12.552400725
O	1.875445680	5.320112002	12.492102513
S	0.726244665	6.289913990	12.661691297
O	9.270162236	7.106008556	13.843254419
O	7.862703283	5.571520500	12.552326734
O	10.295138750	5.305880730	12.541060263
S	9.141751091	6.287925967	12.664757596
H	6.749969215	6.624252468	11.925544013
H	4.230029560	6.833224685	11.264403610
H	12.616268317	6.766163426	11.172702763
H	15.140902799	6.647823435	11.940235075
O	7.702005476	1.124143962	12.629030859
H	7.842800064	0.568248862	13.417516767
O	3.499350649	0.162614270	14.563803674
O	11.990677609	-0.079242180	14.471960661
O	-0.702989818	1.123702598	12.626279593
H	-0.564313825	0.626166648	13.453122054
O	3.504809302	2.646310080	14.236966703
H	6.445722557	3.783943501	11.967714812
O	4.693146560	1.142267011	12.630964683
O	2.330091651	1.209981087	12.574415251
S	3.501006251	1.307659391	13.703965570
O	11.996566485	2.417024640	14.463794305
H	14.845642159	3.717111919	11.973982991
O	13.156458705	1.130528727	12.631617087
O	10.780538428	1.202305408	12.651661413
S	11.954575062	1.165152041	13.757718063
O	4.595556835	10.976371432	13.918251122
O	2.742968364	9.511357551	13.043105390
O	4.965635769	8.951121938	12.626651384
O	4.131009391	8.798144612	14.977973633
S	4.072510953	9.571524879	13.779446863
H	5.050185122	13.517436522	13.250789918
Zr	6.282628667	12.173983713	13.136807223
C	7.411080217	11.659493981	14.954547833
H	7.111555384	10.703401935	15.404208723
C	6.778576993	12.870911105	15.602781208

H	6.074816475	13.432593036	14.872095755
H	7.525367599	13.650129264	15.809990692
H	8.500509440	11.698361183	14.833144888
C	5.891325121	12.591197092	16.819906527
H	5.146038095	11.818606045	16.587512545
H	6.506421384	12.227898143	17.654022062
H	5.354416886	13.492772310	17.143332499

propyl hydrogenolysis TS

Al	0.736692245	4.991311890	8.412863438
Al	4.846396118	7.121345149	7.062771374
O	3.419448556	7.354573044	11.121752098
O	7.716126870	4.999073267	9.604670655
O	3.363726268	6.952328116	8.320288720
O	7.712032061	5.079757515	6.958912379
Al	4.851153521	3.257239308	9.921902604
Al	0.698615803	1.165827750	11.376653205
O	7.638546979	0.959206093	7.274483391
O	3.573415861	3.157485716	8.479945535
O	7.577509803	1.233530994	10.093392578
O	3.496272950	3.068554262	11.118630822
Al	7.781259622	3.166316736	9.911019200
Al	3.527918528	1.172353046	11.103666930
O	4.909600412	0.870172672	7.367055865
O	0.704328620	3.150338422	8.553073287
O	5.054603138	1.356694916	10.077414901
O	0.692178094	2.993319092	11.201939969
Al	3.501183504	4.988980806	8.278270571
Al	7.704448055	7.161266377	7.083980537
O	0.854152200	7.266174595	11.351337500
O	4.836468241	5.073197217	9.685459745
O	0.707480888	6.935301395	8.328981311
O	4.900856626	5.232541817	7.044738602
Al	6.276650782	1.063336292	8.651547115
Al	6.265599271	0.850489478	11.487971612
Al	6.308470686	6.031767135	9.776108155
O	6.238293373	7.112512838	11.214033902
O	6.317446043	7.193378789	8.403369996
Al	2.174829219	7.076277575	9.786115351
Al	2.068261950	7.047496115	6.955129824
Al	2.146010185	2.106215403	8.440997735
O	1.941807971	0.969955949	7.050613061
O	2.028015097	0.966057004	9.920962614
Al	2.078923926	4.070518774	11.186697453
O	2.090694558	5.222018231	9.767410061
O	2.041077111	5.154446941	7.127883663
Al	6.159226279	4.017615999	7.123044715
O	6.310895276	2.982619514	8.579279760
O	6.319431405	3.218284671	11.186547372
H	0.966589937	1.104428178	6.921092436
H	4.976828153	1.454147751	6.590707599
H	7.259363966	1.412174361	6.487538622
H	6.300230301	6.490810163	5.165827271
H	3.509257859	6.357704120	5.235346978
H	0.613872511	7.706461814	5.058793839
H	5.789269435	3.100239759	4.929635057

H	8.258876132	4.866406046	6.174033612
O	6.306878823	7.187336439	5.836281434
O	3.492460815	7.180791507	5.751609744
O	0.622770174	7.031148725	5.753423926
O	6.234888342	2.902864524	5.763932534
Al	9.143153913	4.982437209	8.409989756
Al	13.252726222	7.115162478	7.063459439
O	11.845070708	7.337668085	11.034966457
O	16.125002775	4.991745900	9.611151705
O	11.774655889	6.946331840	8.313414794
O	16.125181984	5.079923228	6.959918178
Al	13.244057453	3.236244060	9.896621990
Al	9.035071880	1.181219987	11.349582699
O	16.046210112	0.960841901	7.208526379
O	11.986219232	3.151886827	8.482234314
O	15.986703270	1.226950990	10.083954914
O	11.911550173	3.058124497	11.123443408
Al	16.223776104	3.149283343	9.889322051
Al	11.935045612	1.152350092	11.146511681
O	13.324587760	0.866060323	7.358964937
O	9.112159516	3.148736263	8.560550121
O	13.461787308	1.295014402	10.076064462
O	9.110257665	2.995571888	11.205919746
Al	11.904253160	4.995198392	8.284046613
Al	16.117779866	7.164056465	7.085144119
O	9.264837048	7.249638082	11.356277685
O	13.260852234	5.053808919	9.694949246
O	9.123028746	6.933389786	8.328068300
O	13.312067073	5.227728175	7.048386639
Al	14.709874330	1.065870852	8.614655330
Al	14.696138402	1.048538850	11.479489333
Al	14.720390377	6.035276428	9.777092041
O	14.641190838	7.126825852	11.218963383
O	14.725258243	7.192868682	8.404113393
Al	10.544610564	7.078832283	9.773912040
Al	10.480166591	7.046006958	6.952158503
Al	10.545960252	2.115280209	8.485819177
O	10.354546131	0.963837169	7.119525244
O	10.435892814	0.957188122	9.985654102
Al	10.491112017	4.078290975	11.206661863
O	10.503738337	5.205059795	9.767721209
O	10.452605465	5.150579858	7.127685390
Al	14.575625659	4.022340873	7.120498204
O	14.721388082	2.976060592	8.579263736
O	14.727459937	3.137580282	11.198925825
H	9.375031641	1.091258077	6.995903700
H	13.359296893	1.467153020	6.593914961
H	15.653037113	1.427209735	6.434791956
H	14.715083051	6.497010706	5.161828053
H	11.919835807	6.362546173	5.224268195
H	9.023196532	7.709737760	5.061755211
H	14.210073530	3.106390570	4.926007558
H	16.679046862	4.866955791	6.179584063
O	14.718787749	7.187836205	5.838099318
O	11.905752970	7.180634251	5.748209045
O	9.036004666	7.030479424	5.752557574

O	14.647026043	2.903651907	5.763684587
Al	0.755308332	13.068261608	8.459562275
Al	4.854093040	15.163198044	7.115557026
O	3.490387109	15.474529175	11.043279511
O	7.707797075	13.063559666	9.682901594
O	3.367091757	15.005512547	8.332723541
O	7.718046769	13.148645506	7.040659730
Al	4.880114403	11.274906780	9.968836166
Al	0.548409807	9.164291954	11.173037363
O	7.633056551	9.029238820	7.205350241
O	3.579961390	11.208955872	8.560875311
O	7.636038026	9.280365292	10.078566936
O	3.509290910	11.050344016	11.185796568
Al	7.777493773	11.236237349	9.976485065
Al	3.566334889	9.183147836	11.188133967
O	4.909735265	8.950439034	7.357835737
O	0.695307432	11.217861821	8.566556536
O	5.059756691	9.286494350	10.080259696
O	0.704375136	10.983995149	11.204504486
Al	3.533288077	13.049188966	8.366804765
Al	7.702048247	15.230745123	7.179367575
O	0.854234689	15.318523355	11.357328777
O	4.895429212	13.070670767	9.747948003
O	0.716324783	14.990398831	8.328620579
O	4.901907210	13.263445497	7.112713349
Al	6.300786059	9.125022701	8.608714317
Al	6.375978554	9.069980385	11.414516133
Al	6.302829848	14.157521304	9.955152003
O	6.091615406	15.246091286	11.273053637
O	6.317743439	15.254057564	8.471490393
Al	2.201809488	15.157507516	9.828006063
Al	2.085595211	15.071888171	6.964648324
Al	2.148623333	10.195802329	8.476185947
O	1.883703334	9.042186124	7.124085679
O	2.039603089	9.021478293	9.995376785
Al	2.089939693	12.104701689	11.241504413
O	2.101443268	13.257623045	9.829351417
O	2.077333172	13.166148561	7.193495893
Al	6.178197062	12.090261538	7.212982414
O	6.316970078	11.001749846	8.637093902
O	6.313423340	11.138365119	11.284074597
H	0.898887792	9.176583135	7.025255585
H	4.972674852	9.557267178	6.596950095
H	7.245342538	9.520506100	6.441653964
H	6.323074440	14.572247124	5.217351285
H	3.543117147	14.309732109	5.306771991
H	0.651174210	15.746525045	5.052392775
H	5.836102679	11.218854013	4.997767170
H	8.267157088	12.940292900	6.256008960
O	6.313506983	15.249340687	5.907214255
O	3.504396745	15.169224943	5.759613392
O	0.627680210	15.087133686	5.761238459
O	6.236495237	10.972701233	5.842364298
Al	9.142601724	13.045715408	8.492376217
Al	13.268406845	15.159538924	7.079596408
O	11.837596477	15.427403536	11.047026609

O	16.131461108	13.065760408	9.674389928
O	11.816110526	14.999222659	8.330555033
O	16.127058388	13.142475983	6.969956718
Al	13.245414409	11.285867656	9.946143062
Al	9.119777936	9.218435634	11.354315967
O	16.043822374	9.029637212	7.205905818
O	11.983977861	11.212025692	8.553658811
O	15.969982557	9.278857597	10.017473189
O	11.920516186	11.073535420	11.188138444
Al	16.207436916	11.240762148	9.895472812
Al	11.947115991	9.176290181	11.132649001
O	13.318742822	8.949998241	7.355713268
O	9.109626583	11.215323782	8.636037683
O	13.462169888	9.346150241	10.073259938
O	9.115417761	11.049727252	11.281697532
Al	11.921308221	13.046786819	8.343842709
Al	16.129417370	15.235388128	7.096798647
O	9.183581978	15.245335459	11.370273310
O	13.301832034	13.088291866	9.764370685
O	9.176680355	14.997639328	8.397952090
O	13.318747273	13.248163002	7.110770069
Al	14.693614498	9.133990077	8.593802952
Al	14.666387566	9.068723647	11.470672752
Al	14.726913622	14.131280343	9.838279876
O	14.579042972	15.245029652	11.274482508
O	14.732835994	15.255042625	8.423725785
Al	10.564870549	15.148266555	9.785941756
Al	10.501429083	15.073574438	6.984423066
Al	10.539359280	10.187578994	8.485949702
O	10.295461762	9.044008843	7.120207614
O	10.453232647	9.026976609	9.963317355
Al	10.515222884	12.118893879	11.249183519
O	10.520762679	13.256794336	9.831226103
O	10.454877017	13.168196233	7.200431262
Al	14.599285109	12.074382045	7.157278103
O	14.723430868	11.043181899	8.626979388
O	14.738026380	11.136192537	11.223485268
H	9.310228293	9.173912179	7.017648488
H	13.380255229	9.547305780	6.586820857
H	15.661725287	9.513795268	6.433958919
H	14.732615647	14.566890378	5.161158370
H	11.928327054	14.327363085	5.274349818
H	9.017096423	15.754268660	5.127332187
H	14.317974882	11.128413017	4.954674067
H	16.709672355	12.917261379	6.214705797
O	14.720144610	15.248850378	5.846230311
O	11.910639754	15.170040301	5.757983942
O	9.033807136	15.090997987	5.832888929
O	14.646024475	10.901251640	5.835274162
O	7.774632732	9.124890799	12.636644303
H	7.955563322	8.502770384	13.372449889
O	12.066912907	10.566332981	14.320851578
H	14.853314240	11.712323489	12.003019277
O	13.173926685	9.112834706	12.623005576
O	10.814863746	9.201037040	12.648179732
S	11.985074819	9.251812737	13.742694299

O	11.995918383	8.073297161	14.557255559
O	-0.699370275	9.117613635	12.559572256
H	-0.515716800	8.604177609	13.369353616
O	0.859016639	15.174234703	13.843768609
O	-0.622832024	13.710788388	12.555275635
O	1.792580289	13.314734275	12.555701957
S	0.694354571	14.366044859	12.666420470
O	9.037732531	15.084808398	13.837130560
O	7.874816841	13.407636053	12.479375557
O	10.286188428	13.383736071	12.560338319
S	9.076485484	14.300956288	12.631560494
H	15.100177077	14.771373724	11.990296442
H	4.391651874	15.078910134	11.208235141
H	12.610116656	14.875469266	11.246304345
O	0.869652503	7.112461659	13.835791389
O	-0.553323548	5.577082270	12.552490622
O	1.875443627	5.320179618	12.491995247
S	0.726244042	6.289892921	12.661180390
O	9.268715982	7.106424261	13.842194842
O	7.862874932	5.571315020	12.552217627
O	10.295535213	5.306589860	12.540964236
S	9.141932310	6.287083466	12.662711336
H	6.746125431	6.626685211	11.926613805
H	4.232763792	6.837018697	11.258853711
H	12.616311986	6.766552493	11.170094749
H	15.140625304	6.648165797	11.940087771
O	7.702396246	1.123730841	12.631704271
H	7.837032212	0.532075848	13.396724506
O	3.498899839	0.162658763	14.563636794
O	11.987671476	-0.080266353	14.471415283
O	-0.703304089	1.123689935	12.626314659
H	-0.564079401	0.624916203	13.452366195
O	3.504655198	2.646433012	14.236927097
H	6.445162643	3.782852029	11.968813855
O	4.692680455	1.142467140	12.631034975
O	2.329890032	1.210187155	12.573746304
S	3.501495111	1.307579445	13.704056995
O	11.995785441	2.416394279	14.463345423
H	14.845824306	3.716857238	11.974043449
O	13.153332848	1.130048939	12.631620861
O	10.773953126	1.200303076	12.653470181
S	11.951468699	1.164648243	13.757185039
O	4.594808759	10.970425829	13.922825463
O	2.738693852	9.511622028	13.045378383
O	4.962222256	8.951083964	12.628568090
O	4.130866466	8.796664086	14.979723430
S	4.065020018	9.561147599	13.776103082
H	4.861696366	13.328459947	12.707493894
Zr	6.199308958	12.204130708	13.153040635
C	7.355333080	11.860966539	15.221393747
H	7.141019353	10.790883581	15.020019520
C	7.165567841	12.115861233	16.725204879
H	7.386173292	13.173526336	16.942267576
H	7.911634165	11.525978090	17.280702268
C	5.754456767	11.764694954	17.200933077
H	4.996344673	12.375964320	16.689735419

H	5.517523843	10.710058756	16.998558716
H	6.542984189	12.955919388	14.883482843
H	5.868262673	13.656636247	14.369863094
H	8.392496649	12.057287933	14.904258447
H	5.647081470	11.935214678	18.280407985

Supplementary References

1. T. D. Kühne, M. Iannuzzi, M. Del Ben, V. V. Rybkin, P. Seewald, F. Stein, T. Laino, R. Z. Khaliullin, O. Schütt, F. Schiffmann, D. Golze, J. Wilhelm, S. Chulkov, M. H. Bani-Hashemian, V. Weber, U. Borštnik, M. Taillefumier, A. S. Jakobovits, A. Lazzaro, H. Pabst, T. Müller, R. Schade, M. Guidon, S. Andermatt, N. Holmberg, G. K. Schenter, A. Hehn, A. Bussy, F. Belleflamme, G. Tabacchi, A. Glöß, M. Lass, I. Bethune, C. J. Mundy, C. Plessl, M. Watkins, J. VandeVondele, M. Krack, J. Hutter, CP2K: An electronic structure and molecular dynamics software package -Quickstep: Efficient and accurate electronic structure calculations. *J. Chem. Phys.* **152**, 194103 (2020).
2. J. VandeVondele, J. Hutter, Gaussian basis sets for accurate calculations on molecular systems in gas and condensed phases. *J. Chem. Phys.* **127**, 1–9 (2007).
3. S. Goedecker, M. Teter, J. Hutter, Separable Dual-Space Gaussian Pseudopotentials. *Phys. Rev. B.* **54**, 1703–1710 (1996).
4. J. P. Perdew, K. Burke, M. Ernzerhof, Generalized Gradient Approximation Made Simple. *Phys. Rev. Lett.* **77**, 3865–3868 (1996).
5. S. Grimme, J. Antony, S. Ehrlich, H. Krieg, A consistent and accurate ab initio parametrization of density functional dispersion correction (DFT-D) for the 94 elements H-Pu. *J. Chem. Phys.* **132**, 1–19 (2010).
6. Chemcraft - graphical software for visualization of quantum chemistry computations. https://www.chemcraftprog.com.
7. M. J. Frisch, G. W. Trucks, H. B. Schlegel, G. E. Scuseria, M. A. Robb, J. R. Cheeseman, G. Scalmani, V. Barone, G. A. Petersson, H. Nakatsuji, X. Li, M. Caricato, A. V Marenich, J. Bloino, B. G. Janesko, R. Gomperts, B. Mennucci, H. P. Hratchian, J. V Ortiz, A. F. Izmaylov, J. L. Sonnenberg, D. Williams-Young, F. Ding, F. Lipparini, F. Egidi, J. Goings, B. Peng, A. Petrone, T. Henderson, D. Ranasinghe, V. G. Zakrzewski, J. Gao, N. Rega, G. Zheng, W. Liang, M. Hada, M. Ehara, K. Toyota, R. Fukuda, J. Hasegawa, M. Ishida, T. Nakajima, Y. Honda, O. Kitao, H. Nakai, T. Vreven, K. Throssell, J. A. Montgomery Jr., J. E. Peralta, F. Ogliaro, M. J. Bearpark, J. J. Heyd, E. N. Brothers, K. N. Kudin, V. N. Staroverov, T. A. Keith, R. Kobayashi, J. Normand, K. Raghavachari, A. P. Rendell, J. C. Burant, S. S. Iyengar, J. Tomasi, M. Cossi, J. M. Millam, M. Klene, C. Adamo, R. Cammi, J. W. Ochterski, R. L. Martin, K. Morokuma, O. Farkas, J. B. Foresman, D. J. Fox, *Gaussian 16 Revision B.01* (2016).
8. Zlochower, I.A.; Green, G.M., The limiting oxygen concentration and flammability limits of gases and gas mixtures. *J. Loss. Prev. Process. Ind.* 22, 499–505 (2009).
