# Supplementary material for: Rapid atom-efficient polyolefin plastics hydrogenolysis mediated by a well-defined single-site electrophilic/cationic organo-zirconium catalyst
Source: Nat Commun. 2022 Nov 23;13:7187. doi: 10.1038/s41467-022-34707-6 (PMC9684440; doi:10.1038/s41467-022-34707-6)
Supplement: Supplementary file 2 — Description of Additional Supplementary Files [file 41467_2022_34707_MOESM2_ESM.pdf]

Description of Additional Supplementary Files

File Name: Supplementary Movie 1
Description: Catalytic hydrogenolysis of 1.93 mL of hexadecane over 0.178 mg of AlS/ZrNp$_2$ (0.05 mol% Zr) at 150°C/2.5 atm H_2 (350 mL vessel) to volatile alkanes in 18 min (Table 1 Entry 7)".
